# Myeloid‐Driven Immune Suppression Subverts Neutralizing Antibodies and T Cell Immunity in Severe COVID‐19

**DOI:** 10.1002/jmv.70335

**Published:** 2025-04-04

**Authors:** Cong Lai, Su Lu, Yilin Yang, Xiaoyu You, Feixiang Xu, Xinran Deng, Lulu Lan, Yuesheng Guo, Zhongshu Kuang, Yue Luo, Li Yuan, Lu Meng, Xueling Wu, Zhenju Song, Ning Jiang

**Affiliations:** ^1^ Department of Emergency Medicine School of Life Sciences, Zhongshan Hospital Fudan University Shanghai China; ^2^ Institute of Infection and Health Fudan University Shanghai China; ^3^ Institute of Emergency Rescue and Critical Care Fudan University Shanghai China; ^4^ Department of Respiratory Medicine Shanghai Jiaotong University School of Medicine, Renji Hospital Shanghai China; ^5^ Shanghai Institute of Infectious Disease and Biosecurity Fudan University Shanghai China

**Keywords:** BCR, COVID‐19, high‐resolution single‐cell transcriptomics peripheral immune responses, humoral immune

## Abstract

The objective of this study was to better understand immune failure mechanisms during *severe acute respiratory syndrome coronavirus 2*, SARS‐CoV‐2 infection, which are critical for developing targeted vaccines and effective treatments. We collected 34 cases representing different disease severities and performed high‐quality single‐cell TCR/BCR sequencing to analyze the peripheral immune cell profiles. Additionally, we assessed antibody‐neutralizing activity through in vitro experiments. Our integrated multiomics analysis uncovers a profound immune paradox in severe COVID‐19: hyperinflammation coexists with immunosuppression, driven by distinct yet interconnected dysregulatory mechanisms. Severe patients develop robust humoral immunity, evidenced by clonally expanded plasma cells producing neutralizing antibodies (e.g., IGHG1‐dominated responses) and antigen‐specific T cell activation. However, these protective responses are counteracted by myeloid‐driven immunosuppression, particularly *CD14*+ *HMGB2+* monocytes exhibiting metabolic reprogramming and HLA‐DR downregulation, coupled with progressive T cell exhaustion characterized by IFN‐γ/TNF‐α hyperactivation and impaired antigen presentation. Importantly, prolonged viral persistence in severe cases arises from a failure to coordinate humoral and cellular immunity—antibody‐mediated neutralization cannot compensate for defective cytotoxic T cell function and monocyte‐mediated immune suppression. These findings highlight the necessity for therapeutic strategies that simultaneously enhance antibody effector functions (e.g., Fc optimization), restore exhausted T cells, and reverse myeloid suppression. They also highlight the importance of vaccines designed to elicit balanced B cell memory and durable T cell responses, which are critical to preventing severe disease progression. By addressing the dual challenges of hyperinflammation and immunosuppression, such approaches could restore immune coordination and improve outcomes in severe COVID‐19.

## 1 Introduction

The COVID‐19 pandemic, triggered by severe acute respiratory syndrome coronavirus 2, Family Coronaviridae, subfamily Orthocoronavirinae, genus *Betacoronavirus*, species *severe acute respiratory syndrome‐related coronavirus*, has posed significant challenges to global health [1]. As the epidemic has progressed, it has become increasingly clear that the immune system plays a critical role in determining the outcome of COVID‐19. The disease spectrum ranges from asymptomatic cases to severe pneumonia and acute respiratory distress syndrome (ARDS), highlighting the complexity of the host immune response [2]. Patients with prolonged viral shedding were mostly high‐risk populations who are usually elderly or with comorbidities, and were prone to severe cases [3, 4]. Few studies have evaluated prolonged viral clearance, though it has the important meaning of preventing transmission, guiding antiviral treatment, and impacting the patient's health. Meanwhile, the severe and critical cases mainly burden the healthcare system, which was attributed to the elderly with incomplete vaccination. According to multiple associated studies, increased proinflammatory cytokines including IL‐6 were detected in COVID‐19 patients, and might initiate viral sepsis and ARDS [5]. One prominent feature of SARS‐CoV‐2 severe infection is lymphopenia and can be reversed when patients recover [6].

Upon SARS‐CoV‐2 infection, the innate immune response is the first line of defense, involving macrophages, dendritic cells (DC), and monocytes that recognize and respond to the virus through pattern recognition receptors [7, 8]. These cells initiate inflammation and produce cytokines to recruit and activate other immune cells. However, in COVID‐19, the excessive activation of this response can lead to a cytokine storm, contributing to severe disease and poor outcomes [9]. Following this, the generation of memory T and B cells is a hallmark of the adaptive immune response, which is crucial for a quick reaction to further SARS‐CoV‐2 infection [10, 11, 12, 13, 14].

Through the T‐cell antigen receptor (TCR), T cells are able to identify antigens, while B cells use antibodies to recognize intact antigens. B‐cell antigen receptors (BCRs) are receptors found on the cell membrane or produced as antibodies by B cells as well. The variable (V), diversity (D), and joining (J) segments of CDR3 gene (V(D)J) recombination marked diversity among TCRs and BCRs. In previous studies of SARS‐CoV‐2, the changes of clonotypic T cell expansion are controversial during the disease recovery, which may be influenced by different proportions of specific T cell subtypes. Specific memory T clonotypic cells were expanded or preserved in different degrees, possibly playing a role in the elimination of SARS‐CoV‐2 [15, 16]. In contrast to TCR analysis, higher B cell clonality consistently remained in COVID‐19 patients compared with healthy controls, supporting the concept that B cells were subjected to unique clonal VDJ rearrangements during infection. It was found that COVID‐19 and healthy controls shared very few B cell clonotypes in single‐cell analysis, especially in the plasma cells, which might be associated with strong antibody response [16].

Recent advancements in high‐resolution single‐cell transcriptomics (scRNA‐seq) have provided unprecedented insights into the immune cell landscape of COVID‐19 patients [17]. Moreover, the application of TCR and BCR sequencing (TCR/BCR‐seq) has further provided insights into the clonal diversity and antigen specificity of T and B cells and how the immune system is mobilized against the virus and how it evolves during the course of infection [18, 19].

As the pandemic continues to evolve, understanding these immune cell changes will remain crucial. In this study, scRNA‐seq and TCR/BCR‐seq data were obtained from individuals, including hospitalized COVID‐19 patients and healthy controls. The disease's immunological underpinnings were profiled, which would enhance our ability to predict, prevent, and treat COVID‐19 and similar infectious diseases.

## 2 Materials and Methods

### 2.1 Study Design and Participants

This study included 34 COVID‐19 patients and 6 healthy donors, with an average age of 79 years, from Zhongshan Hospital, Fudan University. Virus clearance was defined by two consecutive negative RT‐PCR tests. The diagnosis and clinical severity were classified according to the ninth version of the Chinese Clinical Guidance for COVID‐19 Pneumonia Diagnosis and Treatment [20], categorizing cases as asymptomatic, mild, moderate, severe, or critical: (1) Mild short (patients with asymptomatic, mild, moderate diagnosis and viral shedding time ≤ 10 days), (2) Mild long (patients with severe and critical diagnosis and viral shedding time > 10 days), (3) Severe short (patients with asymptomatic, mild, moderate diagnosis and viral shedding time ≤ 10 days), (4) Severe long (patients with severe and critical diagnosis and viral shedding time > 10 days). The inclusion criteria were diagnosed with COVID‐19 Pneumonia. The exclusion criteria were patients (1) with tumors, (2) with rheumatic system diseases, (3) patients who have used immunosuppressants, (4) patients who have undergone stem cell or solid organ transplantation, (5) patients who have undergone biological therapy, (6) and patients who have been vaccinated within 1 year with vaccines other than the COVID‐19 vaccine. All participants voluntarily donated blood samples.

Demographic data, vaccination status, comorbidities, clinical manifestations, prognosis, lab results, and treatments were provided to the study investigators, which was approved by the Ethical Committee of Zhongshan Hospital, Fudan University (B2022‐244R).

### 2.2 Peripheral Blood Mononuclear Cells (PBMCs) Isolation and Data Processing

Peripheral blood was collected, and PBMCs were isolated and cryopreserved for single‐cell RNA sequencing (scRNA‐seq) using the 10× Chromium platform and Illumina NovaSeq. 6000. The data were aligned to the GRCh38 genome with quality control to ensure a median of 6860 cells per sample. Unsupervised clustering and batch effect correction were performed using Seurat and Harmony, excluding low‐quality samples from the analysis [21].

### 2.3 Cell Subset Annotations and Basic Analysis

Following COMBAT Consortium guidelines [22], six immune cell types and their major subsets were classified, with no significant cell cycle effects. Correlation analysis between clinical conditions and cell annotations was performed using the Kruskal–Wallis test, and comparisons across patient phenotypes were made with Student's *t‐*test, visualized in heatmaps and boxplots (*p < 0.05, **p < 0.01, ***p < 0.001, ****p < 0.0001).

### 2.4 Immune Repertoire V(D)J Analysis

BCR/TCR sequences were assembled using Cell Ranger (v.6.0.2) against GRCh38, with low‐quality samples excluded. Data were analyzed in R with scRepertoire and alakazam [23, 24]. Clonotypes with frequencies > 1 were aligned to SARS‐CoV‐2 sequences from IEDB and compared using Clustal Omega [25, 26].

### 2.5 Production of Monoclonal Antibodies and Characterization

BCR heavy and light chain DNA products were cloned into IgG1 and IgK/L vectors, transfected into CHO cells, and the secreted antibodies purified and verified by western blot and ELISA [27]. ELISA assessed antibody–antigen interactions, and GraphPad Prism was used to determine EC50 values and generate magnitude–breadth plots.

### 2.6 Structure Docking Prediction Analysis

Eighteen antibody sequences targeting B cell subpopulations were modeled using structural data from PDB (7zxu) and AlphaFold2 [28, 29] predictions, processed and visualized in PyMOL [30].

### 2.7 Cytokine Analysis

After performing cytokine detection with the MSD (Meso Scale Discovery) immunoassay platform (Meso Scale Diagnostics LLC), cytokine levels were quantified, and data quality was validated. The data were then categorized into experimental groups and analyzed using the R statistical software. The ggplot2 package was utilized to visualize the data. Significance testing between groups was performed using Student's *t‐*test.

## 3 Results

### 3.1 Altered Peripheral Blood Immune Cell Composition in COVID‐19 Patients With Different Disease States

**Figure 1:**
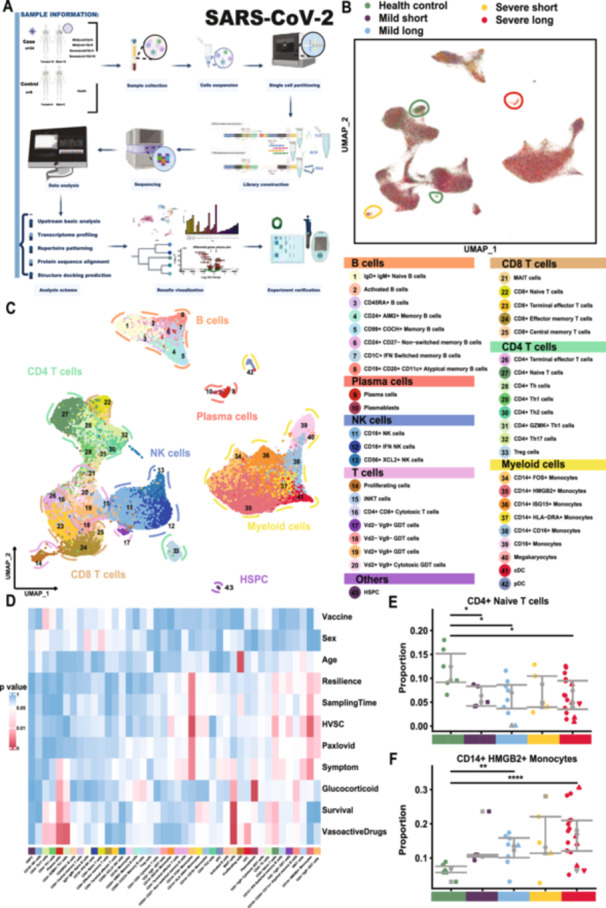
Peripheral blood single‐cell atlas of SARS‐CoV‐2 infected individuals and healthy controls. (A) Overview of the experimental design and analysis workflow. (B) Visualization of cell cluster distributions across different groups, with specific subpopulations highlighted by distinct colors. (C) UMAP plot annotated with cell subpopulations, derived from functional annotation and cluster‐specific high‐expression genes across five major cell types. See Supporting Information S1: Figure 1 for details. (D) Heatmap showing the significance of cell subpopulation distributions across different clinical manifestations, analyzed using the Kruskal–Wallis test. (E) Composition analysis of *CD4*+ Naïve T cells across groups. Green represents healthy controls; purple, mild short; blue, mild long; yellow, severe short; and red, severe long. Different shapes indicate clinical statuses: circles represent unvaccinated survivors, squares vaccinated survivors, upward triangles unvaccinated deceased, and downward triangles vaccinated deceased. Statistical significance was determined using Student's *t‐*test (*p < 0.05, **p < 0.01, ****p < 0.0001). (F) Composition analysis of *CD14*+ *HMGB2+* monocytes across groups.

To investigate the characteristics of the peripheral immune response to Omicron progression, 34 hospitalized convalescent COVID‐19 patients were recruited. According to the clinical severity and recovery speed, patients were divided into 4 groups as following: (1) Mild short: patients with asymptomatic, mild, moderate diagnosis, and viral shedding time ≤ 10 days (*n* = 5); (2) Mild long: patients with asymptomatic, mild, moderate diagnosis and viral shedding time > 10 days (*n* = 8); (3) Severe short: patients with severe and critical diagnosis and viral shedding time ≤ 10 days (*n* = 5); (4) Severe long: patients with severe and critical diagnosis and viral shedding time > 10 days (*n* = 16), while 6 healthy volunteers were also enrolled as health control (HC). A total of 271 828 PBMCs were collected for scRNA‐seq and VDJ immune repertoire sequencing based on 10X Genomics platform (Figure 1A). The median age of the patients was 83.5 years, with 18 men and 16 women (Supporting Information S26: Table 1). After stringent quality control and computational doublet removal, we obtained a combined transcriptome profile with good concordance, with 191 823 cells obtained in the further analysis (QC criteria: minimum of 200 genes and < 10% mitochondrial reads per cell; Supporting Information S1: Figure 1A–F).

**Figure 2:**
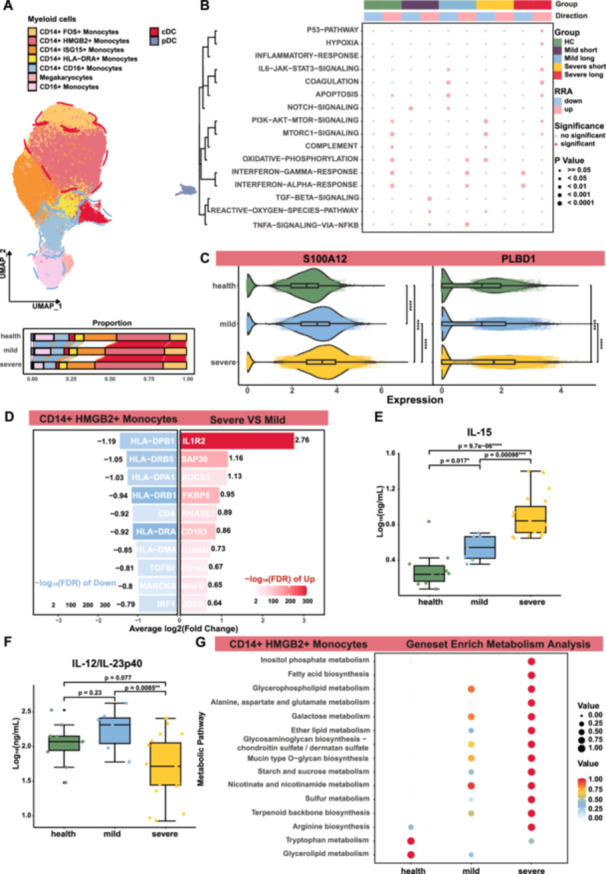
Immunomodulatory dynamics of *CD14*+ *HMGB2+* monocytes in severe cases. (A) UMAP distribution of myeloid cell subpopulations. Subgroups decreasing in severity are outlined with blue dashed lines, while red indicates an increase in proportion, as shown in the bar chart below. (B) Gene Set Enrichment Analysis (GSEA) bubble chart for different groups. RRA methodology integrates various tools such as AUCell, UCell, singscore, ssgsea, JASMINE, viper, GSVApy, zscore, AddModuleScore, and wmean. Blue bubbles indicate downregulated gene enrichments, and pink indicates upregulated. Brighter fill colors signify significance, with bubble size representing the level of statistical significance. (C) Violin plot of gene expression in *CD14*+ *HMGB2+* monocytes. Expression levels are normalized. Statistical tests are performed using Student's *t‐*test (*p < 0.05, **p < 0.01, ***p < 0.001, ****p < 0.0001). (D) Bar chart of top 10 differential genes in *CD14*+ *HMGB2+* monocytes between severe and mild cases. Darker shades represent higher −log10(FDR) values, with red indicating upregulated genes in severe cases and blue indicating downregulation. (E) Box plot of cytokine IL‐15 levels across different severity levels. The *y*‐axis shows log10 normalized concentrations (ng/mL). Different shapes indicate clinical statuses: circles for unvaccinated survivors, squares for vaccinated survivors, upward triangles for unvaccinated deceased, and downward triangles for vaccinated deceased. Comparisons between groups were made using Student's *t‐*test. (F) Box plot of cytokine IL12/IL23p40 across different severity levels. (G) Bubble chart of gene enrichment in metabolic pathways for *CD14*+ *HMGB2+* monocytes, with dot size and color intensity representing normalized scores.

**Figure 3:**
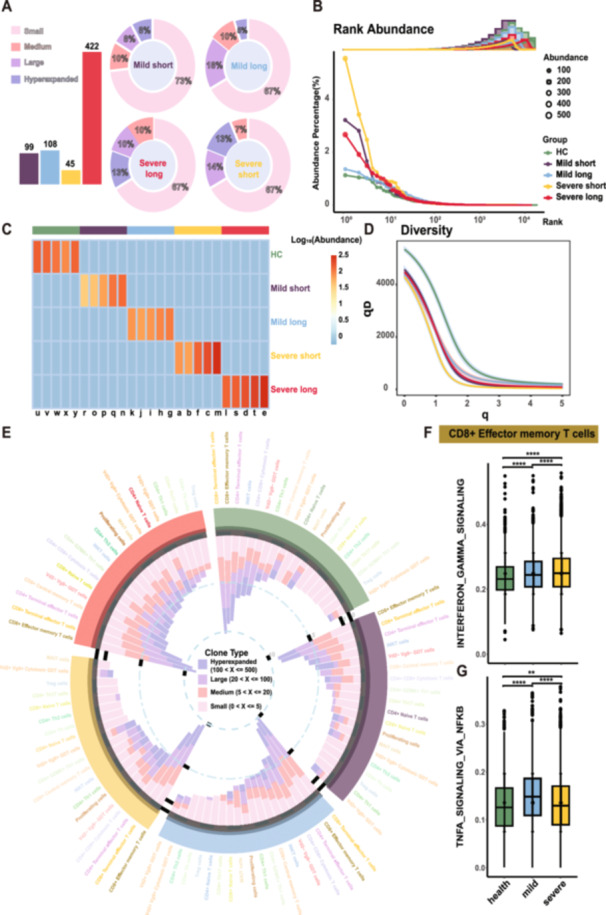
Distribution of SARS‐CoV‐2‐specific TCR clones and immune gene set scoring. (A) Proportional distribution of clonotypes and intergroup statistics of SARS‐CoV‐2‐specific TCRs across different disease states. TCR data, derived from the IEDB database, matches the TRB chain CDR3 amino acid sequences. Clonotypes are categorized as follows: Small (0 < size ≤ 5), Medium (5 < size ≤ 20), Large (20 < size ≤ 100), and Hyperexpanded (> 100 ≤ size ≤ 500, with those exceeding 500 also included in Hyperexpanded). (B) TCR abundance on T lymphocytes across different disease states. Dot size represents the absolute number of clonotypes, with different colors indicating different symptom groups. The bar and curve represent their density distribution. (C) Heatmap of the top 5 most abundant clonotypes in each group. Clone size is log10 normalized. Clonotype abundance is analyzed using combined TRA and TRB CDR3 sequences. (D) Diversity assessment of clonotypes across different groups. Diversity indices range from *q* = 0 to *q* = 5 with a step increment of 0.1 and a 95% confidence interval. (E) Bar chart distribution of TCR clonotypes across different cell types in each group. The height of the bars represents the number of each clonotype, normalized using log10. (F and G) Box plot showing the scoring results of the IFN‐γ and TNFa gene sets in CD8+ effector memory T cells across different disease groups. Statistical significance was assessed using Student's *t‐*test (**p < 0.01, ****p < 0.0001).

**Figure 4:**
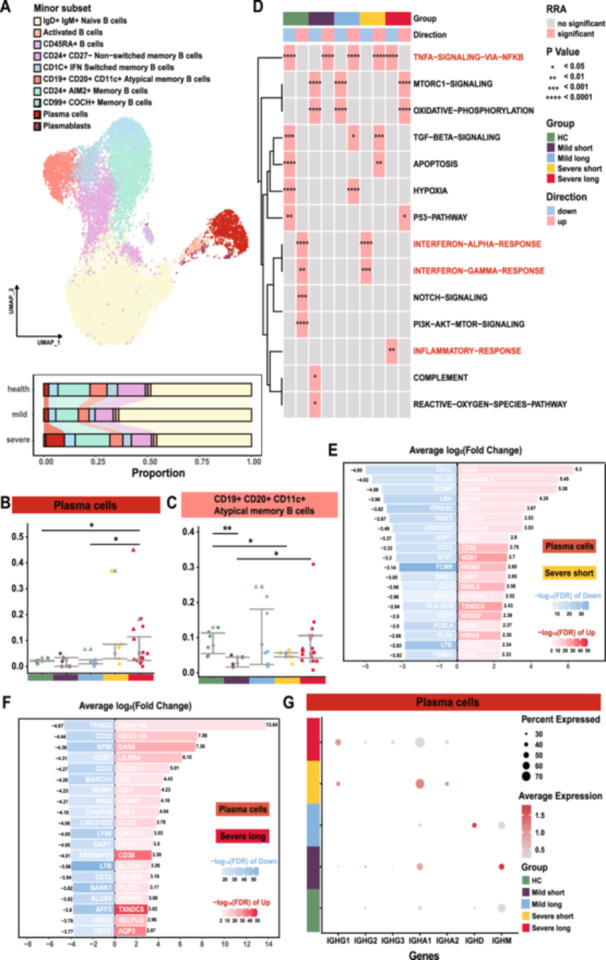
Changes in B cell subpopulations and gene expression related to humoral immunity. (A) UMAP plot showing the distribution of B cell subpopulations and a bar chart of cell proportions across different disease severities. (B) Box plot of plasma cell proportions across groups. Green represents healthy controls; purple, mild short; blue, mild long; yellow, severe short; and red, severe long. Different shapes indicate clinical statuses: circles for unvaccinated survivors, squares for vaccinated survivors, upward triangles for unvaccinated deceased, and downward triangles for vaccinated deceased. Statistical significance was assessed using Student's *t‐*test (*p < 0.05, **p < 0.01, ***p < 0.001, ****p < 0.0001). (C) Box plot showing the relative proportions of atypical memory B cells (CD19+ CD20+ CD11c+) across different groups. (D) Heatmap of gene set scoring for B cells across groups, utilizing RRA methodology which integrates tools like AUCell, UCell, singscore, ssgsea, JASMINE, viper, GSVApy, zscore, AddModuleScore, and wmean. Blue bubbles represent downregulated gene enrichments, and pink indicates upregulated, with brightness indicating significance and bubble size reflecting the level of statistical significance. (E) Bar chart displaying the top 10 differential genes in plasma cells from the severe short group compared to healthy controls. Darker shades represent higher −log10(FDR) values; red indicates upregulated genes in severe cases, and blue shows downregulation. (F) Bar chart displaying the top 10 differential genes in plasma cells from the severe long group compared to healthy controls. Darker shades indicate higher −log10(FDR) values; color coding and shape symbols are consistent with previous charts for continuity. (G) Bubble chart showing the expression of IGHC constant region genes in plasma cells across different groups. Bubble size represents the proportion of cells expressing the gene, and color intensity reflects the level of expression.

The distribution of major PBMC cell types (myeloid cells, T cells, NK cells, B cells, plasma cells) was visualized using the UMAP algorithm (Figure 1B). The preference of each cluster in different patient groups was illustrated: proliferative cells marked by *MKI67* and plasma cells characterized by *MZB1* were more enriched in severe COVID‐19 patients during the disease progression stage (highlighted with yellow and red lines, respectively), while CD8+ naïve T cells, expressing *CD8A*, *LEF1*, and *TCF7*, and Vd2‐ Vg9‐ gamma‐delta T cells (GDT), expressing *KLRC2* and *CMC1*, were predominantly found in HC (highlighted with green lines) (Figure 1B,C and Supporting Information S2–S4: Figures 2, 3 and 4B).

**Figure 5:**
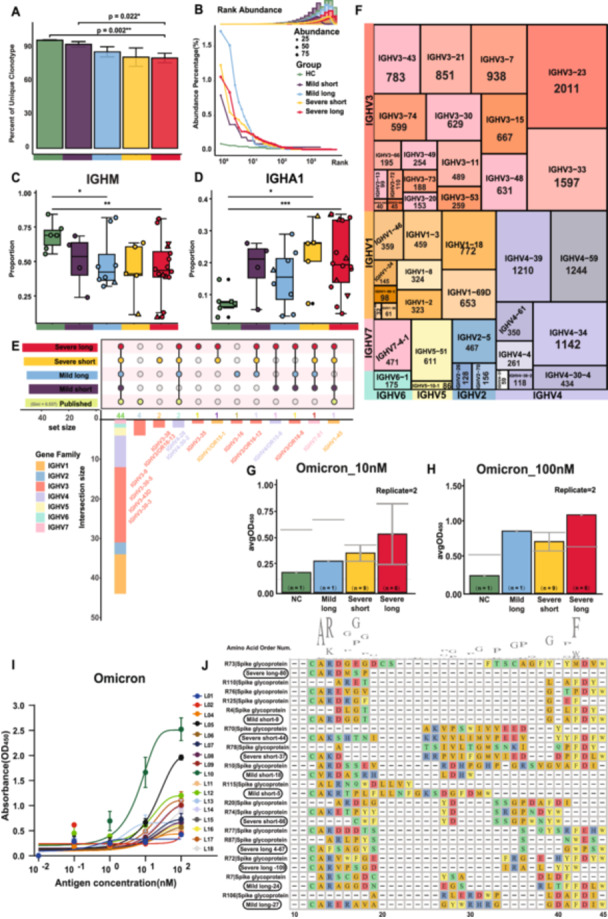
BCR analysis and in vitro antibody binding experiments demonstrating effective humoral immunity. (A) Bar chart showing the proportion of unique BCR clonotypes across different groups, analyzed using Student's *t‐*test (*p < 0.05, **p < 0.01, ***p < 0.001). (B) Dot plot illustrating BCR abundance on B lymphocytes across different disease states. Dot size indicates the absolute number of clonotypes, with color differentiation representing various symptom groups. The accompanying bar and curve show their density distribution. (C) Box plot of the distribution of the BCR heavy chain constant region gene IGHM across groups, with statistical significance assessed using Student's *t‐*test. Different shapes indicate clinical statuses: circles for unvaccinated survivors, squares for vaccinated survivors, upward triangles for unvaccinated deceased, and downward triangles for vaccinated deceased. (D) Box plot showing the distribution of the BCR heavy chain constant region gene IGHA1 across different groups. (E) Upset plot comparing the usage of heavy chain V genes in various case groups against published data from public databases, with Gini index quantifying the skewness of V gene usage from SARS‐CoV‐2 neutralized antibodies. Different fill colors denote different heavy chain gene families. Public database data from AbDab. (F) TreeMap chart displaying the distribution of IGHV genes shared with published public databases, with numbers indicating the count of BCRs in our data set. (G) Grouped bar chart showing the absorbance from in vitro antibody binding ELISA at 10 nM Omicron antigen concentration. The *y*‐axis represents the average optical density from duplicate technical replicates. (H) Grouped bar chart showing the absorbance from in vitro antibody binding ELISA at 100 nM Omicron antigen concentration. The *y*‐axis represents the average optical density from duplicate technical replicates. (I) Fitting curve plot for antibody–antigen interaction across different concentration gradients of Omicron antigen. Each point denotes the average optical density from duplicate technical replicates, with different colors representing different antibodies. (J) Sequence alignment of heavy chain CDR3 region BCR amino acid sequences, displaying positions 10–45 of 27 sequences. “R” denotes the reference sequence, with the target protein specified after “|”. The numbers after disease groups are arbitrary and carry no further significance.

We further annotated 43 distinct cell subsets based on canonical lineage markers and identified 28 subsets that significant correlations with clinical outcomes (Figure 1D and Supporting Information S1 and S2: Figures 1G and 2A). First, subgroups stratified by age, sex, and vaccination status were analyzed. Proliferative cells showed significant associations with age (old age vs. senior elderly, *p* < 0.001; the elderly vs. senior elderly, *p* < 0.01; Supporting Information S5: Figure 5A). Additionally, *CD4*+ naïve T cells, characterized by the expression of *CD4*, *CCR7*, and *LEF1*, as well as invariant natural killer T cells (iNKT), defined by *GNLY*, *NKG7*, *GFGBP2*, *CAMK4*, and *FLT3LG*, were significantly reduced in disease groups compared to healthy controls (HC vs. Mild long, HC vs. Severe short, and HC vs. Severe long, *p* < 0.01; Figure 1E and Supporting Information S3–S5: Figures 3, 4C, and 5B,C).

Analysis of immune infiltration reveals strong intercellular correlations among myeloid cells and high interferon stimulated genes (ISGs) enrichment with genes like *IFITM3*, *SAMHD1*, *RBM47*, *MYD88*, and *PTPN6*, along with significant differences in *CD14*+ *HLA‐DRA+* monocyte expression levels between groups (Supporting Information S6: Figure 6). Myeloid cells, particularly CD14+ HMGB2+ monocytes with high expression of S100A12 and PLBD1, expanded during disease progression, consistent with prior findings of immune activation [16] (Figure 1F and Supporting Information S4: Figure 4A). Notably, CD14+ FOS+ monocytes and CD14+ HMGB2+ monocytes, antigen‐presenting cells, varied significantly across patient and resilience groups (Figure 1F and Supporting Information S5: Figure 5D–G). Th1 cells and mucosal‐associated invariant T cells (MAIT) are strongly correlated with vasoactive drug therapy and survival (Supporting Information S5: Figure 5H–K). FOXP3 regulatory T cells (Tregs) were lower in Paxlovid‐treated patients, indicating effective disease control and inflammatory response (Supporting Information S5: Figure 5L). Severe patients exhibited a substantial drop in CD4+ GZMK + Th1 cells and slower recovery associated with decreased Th2 cells (Supporting Information S5: Figure 5M,N). Atypical memory B cells, which are featured by FGR, IFI30, and TNFRSF1B, were higher in glucocorticoid hormone medication groups, suggesting a role in immune surveillance and inflammation control (Supporting Information S4 and S5: Figures 4B and 5O). Plasma cells were significantly more abundant in severe cases compared to mild cases (Supporting Information S5: Figure 5P). The plasmacytoid dendritic cell (pDCs) cluster decreased in severe patients but was elevated compared to HC (Supporting Information S5: Figure 5Q). Together, we demonstrated highly divergent immune responses across disease severity and the time course of the disease in COVID‐19 patients.

### 3.2 MDSC‐Like Immunosuppressive State of *CD14+ HMGB2+* Monocytes in Proinflammatory Environment Positive Correlated With Disease Severity

We further explored abnormal myeloid cell subsets, annotating megakaryocytes, cDCs, pDCs, and six monocyte subsets (Figure 2A). *CD14*+ *FOS+* and *CD14*+ *HMGB2+* monocytes, associated with proinflammatory states, were elevated in severe patients (Supporting Information S7: Figure 7A–D). In contrast, *CD14*+ *HLA‐DRA+* monocytes, *CD14* + CD16+ monocytes (which represent an intermediate transitional state in antiviral immune responses), CD16+ monocytes, and cDCs significantly decreased only in severe patients compared to HC (Supporting Information S7: Figure 7E–J). Additionally, compared with mild group, the proportion of *CD14*+ *HMGB2+* significantly increased, whereas CD16+ monocytes, pDCs, and cDCs significantly declined in severe patients (Supporting Information S7: Figure 7D,F,I,J). Our findings are consistent with previous studies that proved *S100A*‐high, *HLA‐DR*‐low *CD14+* monocytes (i.e., MDSCs) enriched in severe patients [31, 32].

Gene set enrichment analysis revealed upregulation of TNF‐α signaling through NF‐κB, particularly in the mild short group, and overexpression in interferon response pathways in the mild long group. Severe long cases showed an overreaction of the immune system with IL‐6 cytokine storm and hypercoagulability (Figure 2B). High expression of calprotectin (S100A8/A9) and altered metabolic pathways (arginine and tryptophan metabolism) characterized severe cases, indicating an immunosuppressive state [33]. *CD14*+ *HMGB2+* monocytes showed significant variations in *PLBD1*, *PLAC8*, *CD163*, *MAFB*, *IL1R2*, *HLA‐DRA,* and *S100A8/9/12* expression across groups (*S100A12*, *p* < 0.001; *PLBD1*, *p* < 0.001; Figure 2C; *S100A8/A9*, *PLAC8*, *CD163*, *MAFB*, *IL1R2*, *HLA‐DRA*, *p* < 0.001; Supporting Information S8: Figure 8A–G). The immunosuppressive function of MDSCs is characterized by high expression of arginase 1 Arg‐1, which can inhibit the production of SARS‐CoV‐2‐specific IFN‐γ by T cells [34, 35, 36]. Severe patients had increased IL‐1, decreased HLA‐DRA, and enriched arginine biosynthesis and tryptophan metabolism, consistent with MDSC metabolic traits (Figure 2D,G). Significant differences in cell interactions, such as those involving the *THBS1*(coding for Thrombospondin‐1)–*LRP1* (coding for low‐density lipoprotein receptor‐related protein 1) axis in severe cases, suggested complex immune interactions and potential dysregulation (Supporting Information S9 and S10: Figures 9 and 10A).

Cytokine and chemokine assays showed elevated IL‐15, IL‐17A, IL‐23, and CXCL11 (I‐TAC) in severe cases, indicating a robust inflammatory response (Figure 2E,F and Supporting Information S10: Figure 10B,C).

### 3.3 TCRs of Effector Memory T Cells and *GZMK*+ Th1 Cells Predict Cellular Exhaustion Due to Excessive Immune Suppression

To investigate whether severe individuals who may experience impaired antigen presentation due to the decremented HLA‐DR would further influence T cell priming, TCR‐seq data of 72 484 valid cells was analyzed. Clonotype proportion analysis revealed a notable TCR hyperexpansion predominantly in the severe COVID‐19 group, with a peak concentration of 13% (Figure 3A). Previous studies have established that antigen‐binding TCRs are primarily determined by the CDR3 regions of the TRA and TRB chains [37]. Our cross‐analysis with the TRB chain CDR3 amino acid sequences from the IEDB database identified that SARS‐CoV‐2‐specific TCRs were predominantly in the severe long COVID‐19 group, with 422 overlaps.

Regarding clonotype abundance, the mild COVID‐19 groups characterized by rapid recovery exhibited notable clonotype enrichment (Figure 3B). Specially, the predominant clonotype in the severe short group constituted 5.55% of the TCR repertoire, encompassing 528 cells (Supporting Information S27: Table 2). Conversely, in the severe long group, the most prevalent clonotype comprised 551 cells. In comparison, the mild subgroups displayed fewer cells for their top clonotypes, with 221 and 177 cells, respectively. Comparative analysis of the top 5 most expanded clonotypes across the groups revealed significant heterogeneity in clonotype distribution (Figure 3C and Supporting Information S11: Figure 11A–F). Additionally, diversity evaluation demonstrated a decline in diversity between the disease groups and the healthy group, indicating that all disease groups had an active immunological state and supporting focused clonal proliferation of T cells in response to the viral infection (Figure 3D). TCR diversity estimation for samples also appeared to decline as the disease progressed, suggesting more convergent clonal selection in the severe group (Supporting Information S11: Figure 11G). Significant differences in diversity were also noted, especially in the severe long group, which had a high degree of TCR heterogeneity, indicating a prolonged fight between the host immune system and the virus.

Combined with RNA‐seq data, we found that clonal expansion was mainly concentrated in CD8+ effector memory T cells and terminal effector cells, underscoring their critical role in antiviral immunity (Figure 3E). Furthermore, we identified that IFN‐γ was highly expressed in over 10% of these two cell populations. Notably, elevated IFN‐γ expression in clonally expanded CD8+ terminal effector T cells was primarily associated with the mild symptom group (Supporting Information S11 and S12: Figure 11A and 12A–C). Additionally, clonal expansion across all T lymphocyte subsets was exclusively observed in the severe long group. Medium clonotype expansion of CD8+ naïve T cells and *CD4*+ *GZMK*+ Th1 cells was specifically detected in disease groups, whereas medium expansion of Tregs was predominantly associated with the long‐positive groups (Mild long and Severe long) (Figure 3E).

Gene expression analyses highlighted increased IFN‐γ and TNF‐α in CD8+ effector memory T cells in severe cases, linked to inflammation and disease severity (Figure 3F,G and Supporting Information S13: Figure 13A). Pathway enrichment showed upregulation of immune activation pathways and downregulation of TGF‐β‐related pathways in the severe long group, indicating excessive immune activation and potential T cell exhaustion (Supporting Information S14: Figure 14). Differential gene expression analysis revealed upregulation of immune activation genes (e.g., *JUN*, *JUND*, *GIMAP1/2/4/5/7*, *TGFB1*) and downregulation of immune regulation genes in severe cases (Supporting Information S13: Figure 13B,C). For *CD4*+ *GZMK* + Th1 cells, the severe short group showed increased TNFR1‐mediated NF‐κB signaling and TLR pathways, while the severe long group exhibited downregulation of antigen cross‐presentation and IFN‐γ signaling pathways, suggesting impaired immune responses and disease progression (Supporting Information S15: Figure 15).

In summary, our findings reveal divergent immune responses across disease severity in COVID‐19 patients, with severe cases showing pronounced immune activation, dysregulation, and potential exhaustion.

### 3.4 B Cell Dynamics and Diversity in COVID‐19 Patients

To examine the cellular diversity of peripheral B cells in COVID‐19 patients, we analyzed 19 691 single B cells, identifying clusters of naïve, memory, activated B cells, plasmablasts, and plasma cells (Figure 4A and Supporting Information S16: Figure 16A–H). We found that the proportions of plasma cells significantly increased in severe COVID‐19 patients (Figure 4A). Additionally, plasma cells and plasmablasts expressing *CD38*, *MZB1*, and *JCHAIN* positively correlated with disease severity and recovery time (HC vs. Severe long and Mild long vs. Severe long, *p* < 0.05; Figure 4B and Supporting Information S4 and S16: Figures 4B and 16D).

Interestingly, CD24+ CD27− nonswitched memory B cells and CD19+ CD20+ CD11c+ atypical memory B cells were significantly reduced in rapidly recovering patients (Mild short and Severe short vs. HC, *p* < 0.05; Figure 4C and Supporting Information S4 and S16: Figures 4B and 16G). Compared to the mild group, these memory B cell subsets showed a tendency to increase (Mild short vs. Severe long, *p* < 0.05; Supporting Information S16: Figure 16G). Similarly, CD1C+ IFN‐switched memory B cells, characterized by high expression of *LILRA4*, *KLK1*, *GRN*, and *PPP1R14A*, also exhibited an upward trend (Mild short vs. Severe short, *p* < 0.05; Mild short vs. Severe long, *p* < 0.01; Supporting Information S4 and S16: Figures 4B and 16H). These findings suggest an activation of humoral immune memory.

Gene set scoring revealed that immune response and TNF‐α signaling through NF‐κB were downregulated in the severe long group, while these pathways were upregulated in other groups (Figure 4A,D and Supporting Information S16 and S17: Supplementary Figures 16I and 17A–D). Differential gene expression analysis indicated diverse patterns in plasma cells and CD19 + CD20 + CD11c+ atypical memory B cells across groups (Figure 4E,F and Supporting Information S17 and S18: Figures 17E–G and 18A,B). Severe long patients for plasma cells exhibited overexpression of pathways related to p53 signaling, lysosomes, and increased protein export and ER protein processing, potentially reflecting excessive antibody production (Supporting Information S18: Figure 18C). Conversely, BCR signaling was downregulated in severe patients and elevated in mild groups. Notably, the *IGHG1* gene was highly expressed exclusively in the severe groups, with an expression proportion exceeding 40%, while the *IGHA1* and *IGHA2* genes were highly expressed only in the long‐recovered groups (Figure 4G).

Next, we collected 28 232 BCR sequences for BCR repertoire profiling. Clonal expansion increased with disease severity, particularly in the severe long recovery group (Figure 5A and Supporting Information S19: Figure 19A–C). The mild long group displayed a tendency for greater abundance (Figure 5B). Consistent with RNA‐seq result, *IGHA1* gene expression rose in severe cases (HC vs. Severe long, *p* < 0.001; Figure 5D), while *IGHM* levels decreased with severity and longer recovery times (HC vs. Severe long, *p* < 0.01; HC vs. Mild long, *p* < 0.05; Figure 5C). High *IGHG1* levels were associated with prolonged recovery periods (Supporting Information S19: Figure 19D).

We then integrated our BCR‐seq data with reported SARS‐CoV‐2‐specific antibody data, finding that heavy chain V gene usage was concentrated in *IGHV3* and *IGHV4* families. The severe long group favored *IGHJ4* and *IGHJ6*, while the severe short group preferred *IGLJ3* and *IGLJ1* (Figure 5E,F and Supporting Information S20: Figure 20). Although SARS‐CoV‐2 neutralizing antibody levels do not always correlate with disease severity, we observed that primary clonal expansions occurred in plasma cells and various memory B cell subsets (Supporting Information S21: Figure 21A).

We produced 17 recombinant monoclonal antibodies (mAbs) from dominant BCRs and noted that the most effective antibodies, L05 (IGHV3‐49) and L10 (IGHV3‐49), exhibited stronger binding abilities, particularly in severe cases. This suggests successful antigen targeting due to extensive antigen stimulation (Figure 5G–I and Supporting Information S21: Figure 21B–F). Heavy chain CDR3 sequence similarity analysis revealed significant overlap in the CDR3 region of BCR sequences from severe cases (Supporting Information S22: Figure 22). Germline analysis indicated that, despite some evolutionary distance, L10 exhibited strong binding efficacy (Supporting Information S21: Figure 21G).

Amino acid sequence analysis of large expansion clonotypes revealed conserved residues at key positions, potentially contributing to effective binding (Figure 5J). Structural analysis of the 17 mAbs complexed with the Omicron BA.4/5 RBD identified four primary binding modes, with specific residues like glutamic acid and arginine influencing binding strength. Antibodies L01, L06, L12, and L18 demonstrated strong binding due to multiresidue interactions (Supporting Information S23: Figure 23A,B).

These findings collectively confirm that despite the severity of COVID‐19, effective humoral immunity persists, and dominant immune responses can effectively target the virus.

## 4 Discussion

Understanding how our immune system fights SARS‐CoV‐2 viruses, which is still not fully understood, will be very important as we continue to deal with new variants. Previous studies have revealed significant changes in peripheral immune cells function and distribution, which can influence the course and severity of the disease after SARS‐CoV‐2 infection. Severe infections frequently result in reduced or malfunctioning T cells, which are essential for recognizing and removing contaminated cells. In particular, there is often a decline in the quantity of *CD4*+ T cells and CD8+ T cells, which play different roles in planning and carrying out the immunological response. The capacity to develop a successful immune response against the virus may be hampered by this decline. T lymphocytes showed a decrease in proliferative cells, *CD4*+ naïve T cells, and *CD4*+ *GZMK*+ Th1 cells with increasing severity. As disease severity increased, various *CD14*‐expressing monocyte subsets showed varying degrees of elevation, including *CD14*+ *HMGB2+* monocytes, *CD14*+ *HLA‐DRA+* monocytes, *CD14*+ *FOS+* monocytes, *CD14*+ *ISG15+* monocytes, and *CD14*+ CD16+ monocyte.

During the 4 years of the COVID‐19 pandemic, the disease panorama has changed substantially due to the emergence of new, dominant virus variants. Our findings align with emerging evidence that TLR‐driven hyperinflammation and immunosuppression are central to severe COVID‐19 [38, 39]. TLR3/7 recognition of SARS‐CoV‐2 RNA activates NF‐κB and IRF pathways, triggering excessive production of IL‐6 and TNF‐α—a hallmark of cytokine storms observed in severe disease [39, 40]. This aligns with Choudhury et al. [41, 42], who demonstrated that dysregulated TLR signaling exacerbates systemic inflammation by amplifying NF‐κB‐dependent cytokine release. Notably, our observation of myeloid suppression (e.g., HLA‐DR ↓ monocytes) mirrors studies showing that persistent TLR activation induces monocyte exhaustion, paradoxically dampening antiviral responses despite ongoing inflammation [43].

Therapeutic strategies targeting these pathways are critical. For instance, NF‐κB inhibitors (e.g., dexamethasone) and IL‐6R blockers (e.g., tocilizumab) have shown efficacy in mitigating cytokine storms, corroborating our data linking IL‐6/TNF‐α hyperactivation to poor outcomes [42, 43]. Furthermore, natural compounds like curcumin and resveratrol, which suppress TLR‐NF‐κB signaling [42], may complement existing therapies by tempering hyperinflammation without exacerbating immunosuppression. However, as our study reveals, restoring immune balance requires not only cytokine modulation but also reversing myeloid dysfunction (e.g., metabolic reprogramming in *CD14* + *HMGB2*+ monocytes) and rescuing exhausted T cells—a dual approach supported by recent trials combining JAK inhibitors with PD‐1 agonists.

Regarding B lymphocytes, memory B cells expanded with disease severity. This includes *CD1C*+ IFN‐switched memory B cells and *CD24* + *AIM2* + memory B cells, which were more prevalent in severe cases compared to milder ones. Though B cells are the producers of the key member of humoral immunity, the quantity and diversity of antibodies are not always in accord with the change of B cells. Here, for the first time, we collected clonal expanded BCR, produced the recombinant antibodies, and proved the effective binding ability of these antibodies to either Wuhan or Omicron S trimers. Moreover, even in severe cases, there are higher plasma cell counts and a diversity of BCR repertoires that conform to effective humoral immune systems, which is consistent with earlier reports showing normal antibody levels in severe COVID‐19 patients. Therefore, the humoral immunity alone is obviously not sufficient for successful handling of the SARS‐CoV‐2 infection. We hypothesized that several factors could hinder effective viral control during disease progression. One significant reason could be related to the immune system's overall functionality. A hyperinflammatory response, in which the immune system becomes excessively active and maladaptive, is frequently brought on by severe COVID‐19. This cytokine storm has the potential to harm tissues and organs, interfere with regular immune responses, and decrease the effectiveness of antibodies [9, 44]. An overabundance of inflammation can hinder the antibodies' capacity to adhere to the virus and neutralize it.

Last but not least, the immune response's timing is crucial. In extreme circumstances, a delayed activation of adaptive immunity may prevent the immune system from mounting a sufficient defense. It may be difficult for the immune system to catch up and successfully control the infection if the virus has already caused severe harm by the time enough antibodies are created. Our data together demonstrate the intricate relationship that exists between SARS‐Cov‐2 and the complex host immune system. Despite no significant reduction of humoral immune response was observed in this study, it is unclear whether the changes in the host immune system, particularly the humoral immunity, can fully return to normal levels because there is no long‐term follow‐up of the patients who were discharged. It is also unclear whether these patients can effectively produce humoral immunity in the face of subsequent new coronavirus variants and whether their humoral immunity will eventually become abnormal in the face of ongoing infection by new coronavirus mutant strains.

Relatively small sample size, which affects the generalizability and statistical significance of the results, is one of the limitations of this study. Additionally, longitudinal sampling of the same patients at different infection time points was lacking, preventing dynamic observation of immune cell changes over time. Future research should consider increasing the sample size and implementing longitudinal tracking to better elucidate the dynamic changes in immune cells across different stages of infection and their impact on disease progression. With the continuous in‐depth analysis of the interaction mechanism between SARS‐CoV‐2 and the host, it will help to develop more effective therapy strategies and ultimately defeat viruses.

## Author Contributions

Cong Lai contributed to methodology development, data analysis, quality control, visualization, statistical analysis, and manuscript writing. Su Lu contributed to methodology design, sample collection, library construction, and clinical data processing. Yilin Yang participated in library construction and sequencing. Xiaoyu You performed sequence alignment and data quality control. Xinran Deng conducted cell–cell communication analysis and statistical data analysis. Feixiang Xu and Lulu Lan were responsible for sample collection and sequencing. Yuesheng Guo carried out in vitro experimental validation. Zhongshu Kuang, Yue Luo, and Li Yuan contributed to clinical records curation and methodological support. Professor Lu Meng contributed to manuscript writing, project supervision, investigation, and manuscript revision. Professor Xueling Wu oversaw project coordination, clinical data curation, and manuscript revision. Professors Zhenju Song and Ning Jiang contributed to conceptualization, study design, methodology development, investigation, funding acquisition, project supervision, and manuscript revision. All authors approved the final manuscript.

## Ethics Statement

The protocol was approved by the Medical Ethics Committee of Zhongshan Hospital, Fudan University (B2022‐244R).

## Conflicts of Interest

The authors declare no conflicts of interest.

## Supporting information


**Supporting Figure 1.** Quality control, batch effect correction, cell annotation, and major cell type proportions.

**Supporting Figure 2.** Feature plots of canonical gene markers.

**Supporting Figure 3.** Dot plot of gene markers for NK cells and T lymphocytes.

**Supporting Figure 4.** Visualization of cell annotation marker genes.

**Supporting Figure 5.** Analysis of cell subpopulation proportions under different clinical indications.

**Supporting Figure 6.** Immune cell infiltration analysis and correlation analysis of ISGs gene set scores.

**Supporting Figure 7.** Relative cell proportions of myeloid cells under varying disease severities.

**Supporting Figure 8.** Violin plots of MDSC‐like marker expression in *CD14*+ *HMGB2*+ monocytes across symptom severity groups.

**Supporting Figure 9.** Differential cell interactions among myeloid cell subpopulations across different groups.

**Supporting Figure 10.** Differential ligand‐receptor interactions across various clinical conditions.

**Supporting Figure 11.** UMAP visualization of T lymphocytes, NK cells, and TCR clonotype distributions.

**Supporting Figure 12.** Clone expanded distribution and gene expression dot plots of T lymphocytes and NK cells.

**Supporting Figure 13.** Immunogenomic analysis and gene set scoring significance for T lymphocytes and NK cells.

**Supporting Figure 14.** Pathway enrichment visualization of differential genes in CD8+ effector memory T cells across different disease groups.

**Supporting Figure 15.** Differential analysis of *CD4*+ *GZMK*+ Th1 cells across various groups.

**Supporting Figure 16.** Composition analysis and pathway enrichment scores of B cells.

**Supporting Figure 17.** Differential gene set scoring and analysis of B cells subpopulations across disease severities.

**Supporting Figure 18.** Differential gene analysis of plasma cells and CD19+ CD20+ CD11c+ atypical memory B cells.

**Supporting Figure 19.** BCR clonal status analysis and clonotype diversity assessment.

**Supporting Figure 20.** BCR VJC gene usage frequency statistical analysis.

**Supporting Figure 21.** In vitro determination of neutralizing antibody binding and antigen potency against the SARS‐CoV‐2 prototype strain.

**Supporting Figure 22.** Analysis of BCR heavy chain amino acid CDR3 sequence similarity and clonal germline evolution.

**Supporting Figure 23.** Predicted commutative crystal structures of neutralizing antibodies with SARS‐CoV‐2 Omicron RBD using AlphaFold2.

Supporting Figure Captions 250210 revised.

Supporting Materials & Methods Revised.

**Supporting Table 1.** Overview of basic information on clinical samples. Sheet named “Supplementary_Table_1A” provides details on clinical sample information, sheet named “Supplementary_Table_1B” and “Supplementary_Table_1C” presents statistics on sample quality, and sheet named “Supplementary_Table_1D” includes cell count statistics.

**Supporting Table 2.** Top 5 most abundant clonotypes information on each group.

**Supporting Table 3.** Sequence information of expressed monoclonal antibodies in vitro.

Supporting Table Captions 241125.

## Data Availability

All sequencing data have been uploaded to the China National Center for Bioinformation (CNCB) Genome Sequence Archive (GSA) database under the Accession Number: PRJCA028987.
