# Supplementary figures and images for: Myeloid‐Driven Immune Suppression Subverts Neutralizing Antibodies and T Cell Immunity in Severe COVID‐19
Source: J Med Virol. 2025 Apr 4;97(4):e70335. doi: 10.1002/jmv.70335 (PMC11969634; doi:10.1002/jmv.70335)

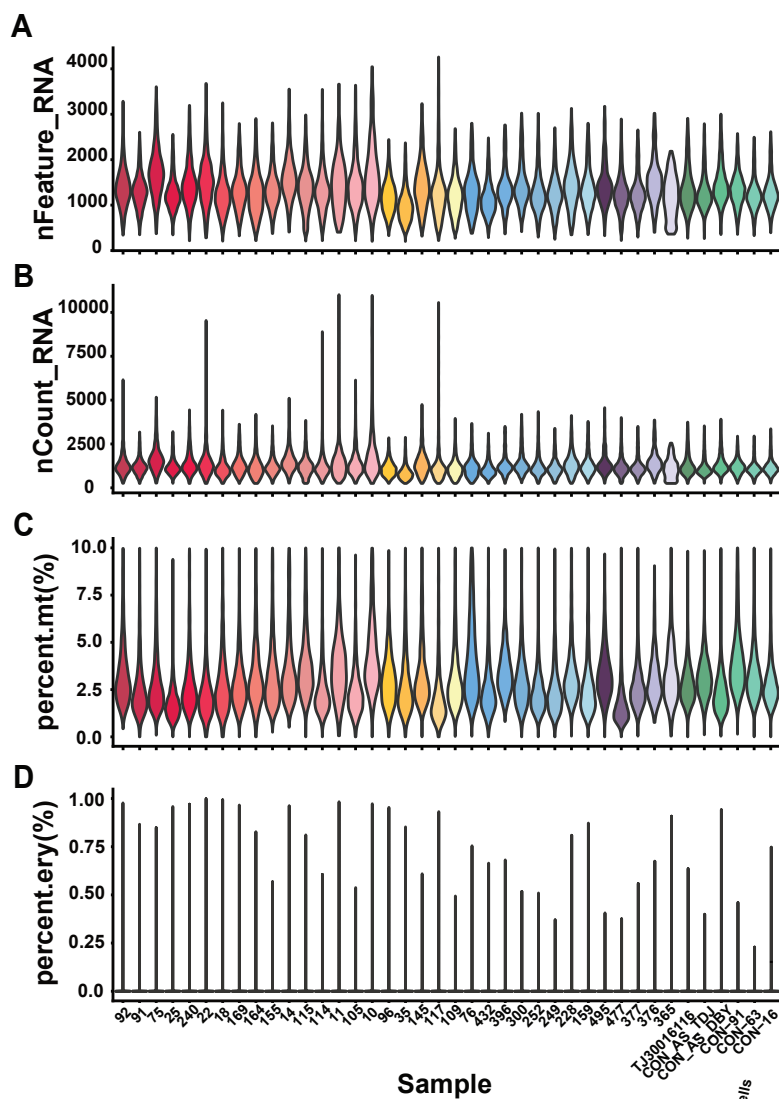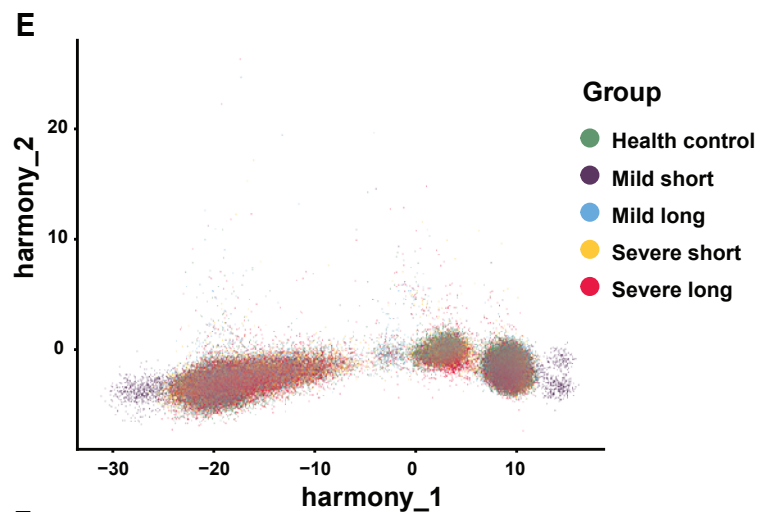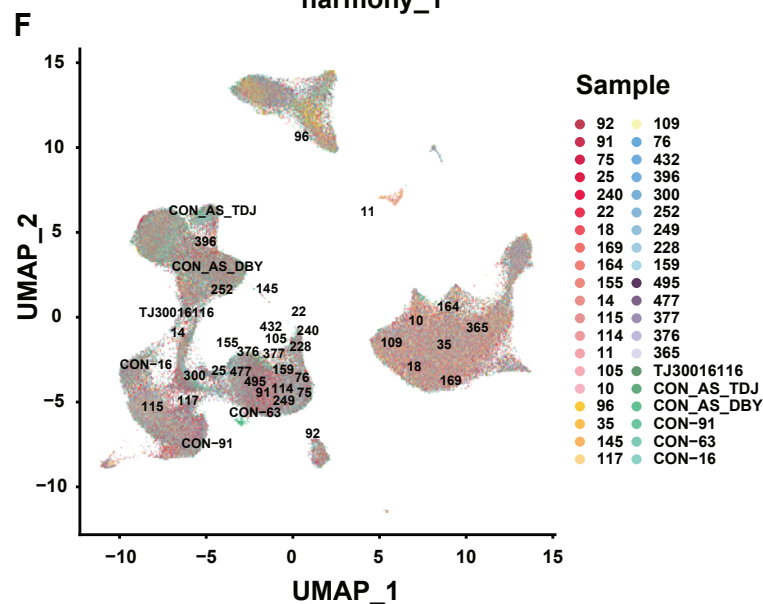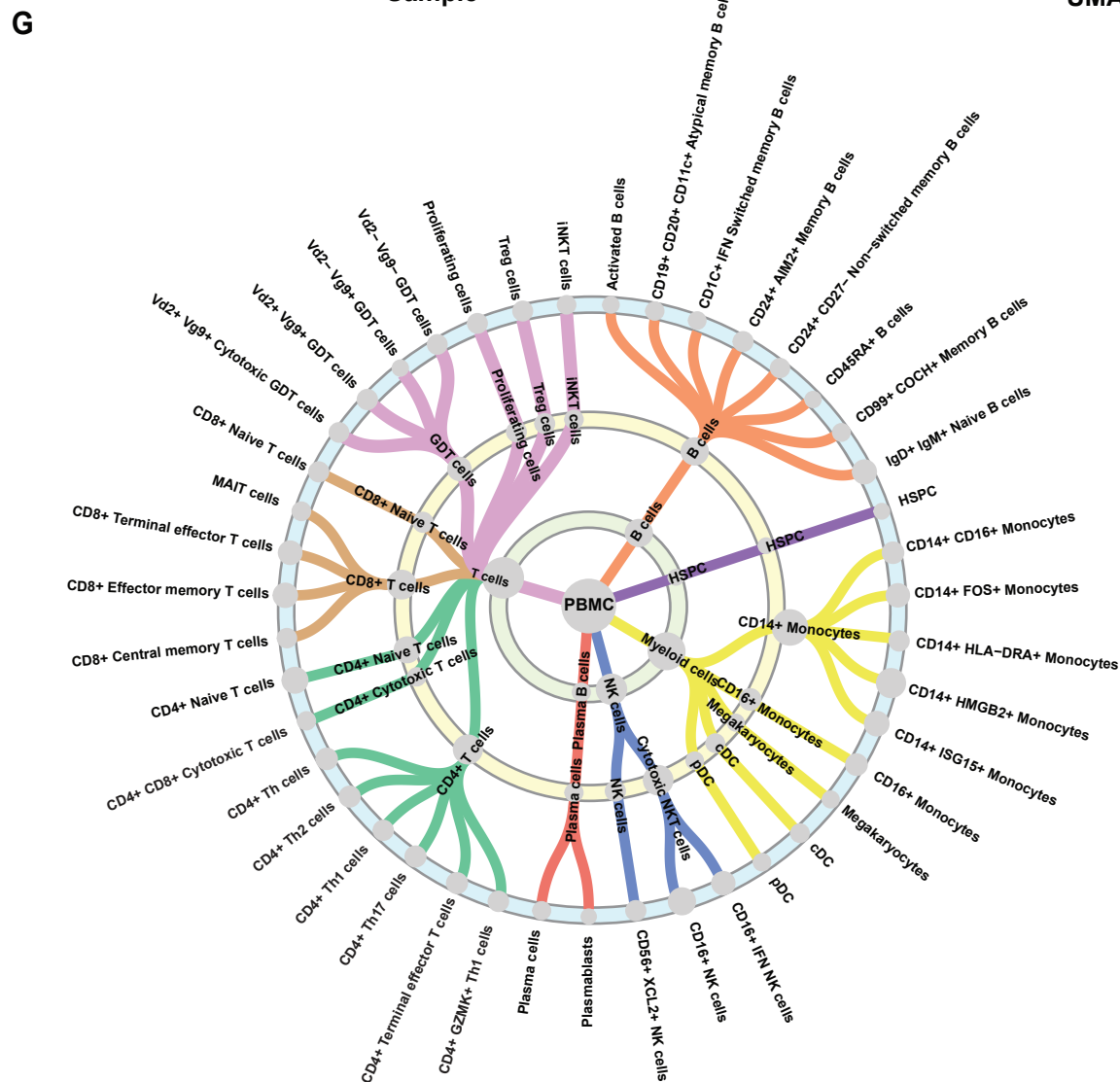

Supplement: Supplementary file 1 — Supporting Figure 1. Quality control, batch effect correction, cell annotation, and major cell type proportions. [file JMV-97-e70335-s015.pdf]

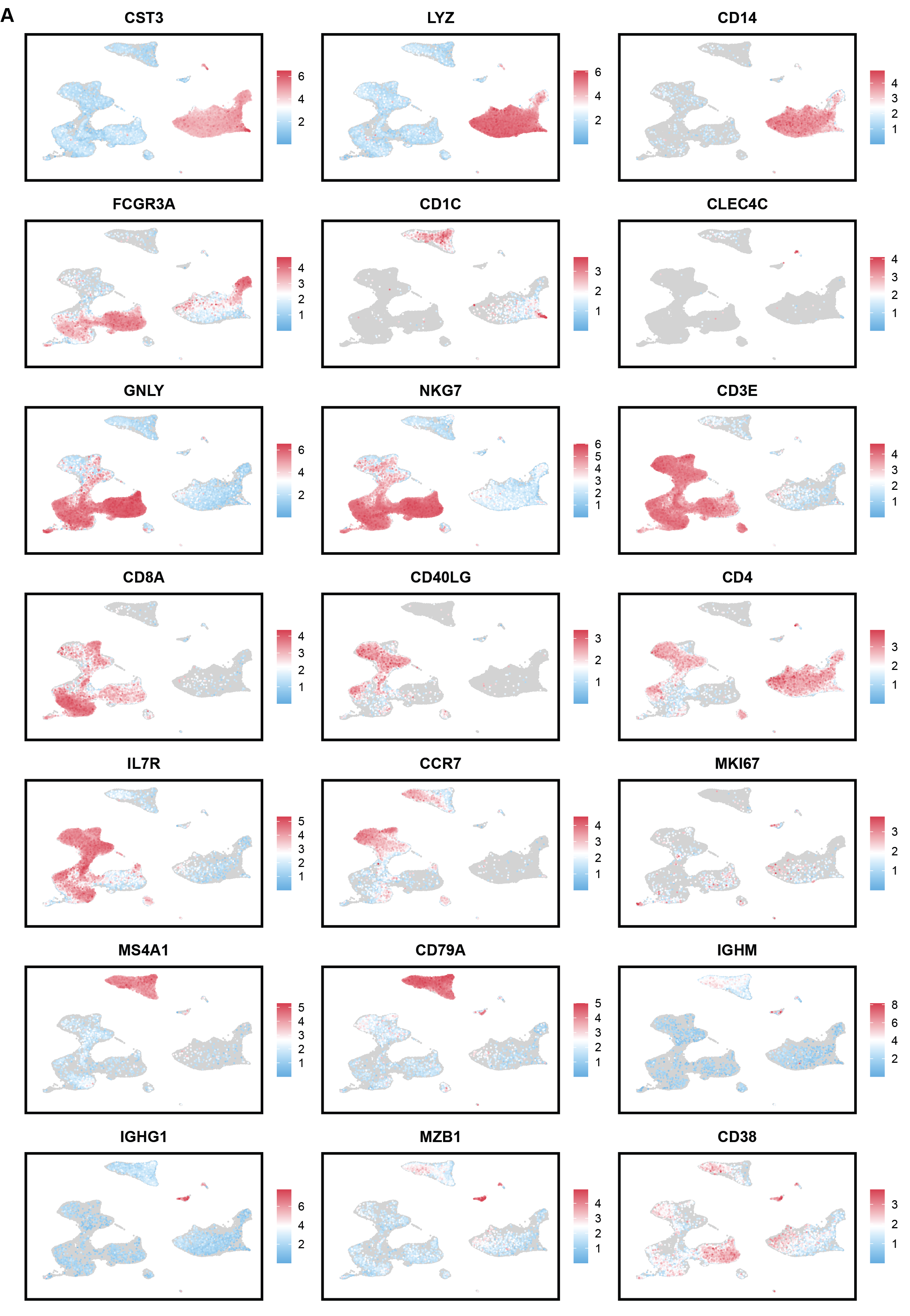

Supplement: Supplementary file 2 — Supporting Figure 2. Feature plots of canonical gene markers. [file JMV-97-e70335-s003.png]

A

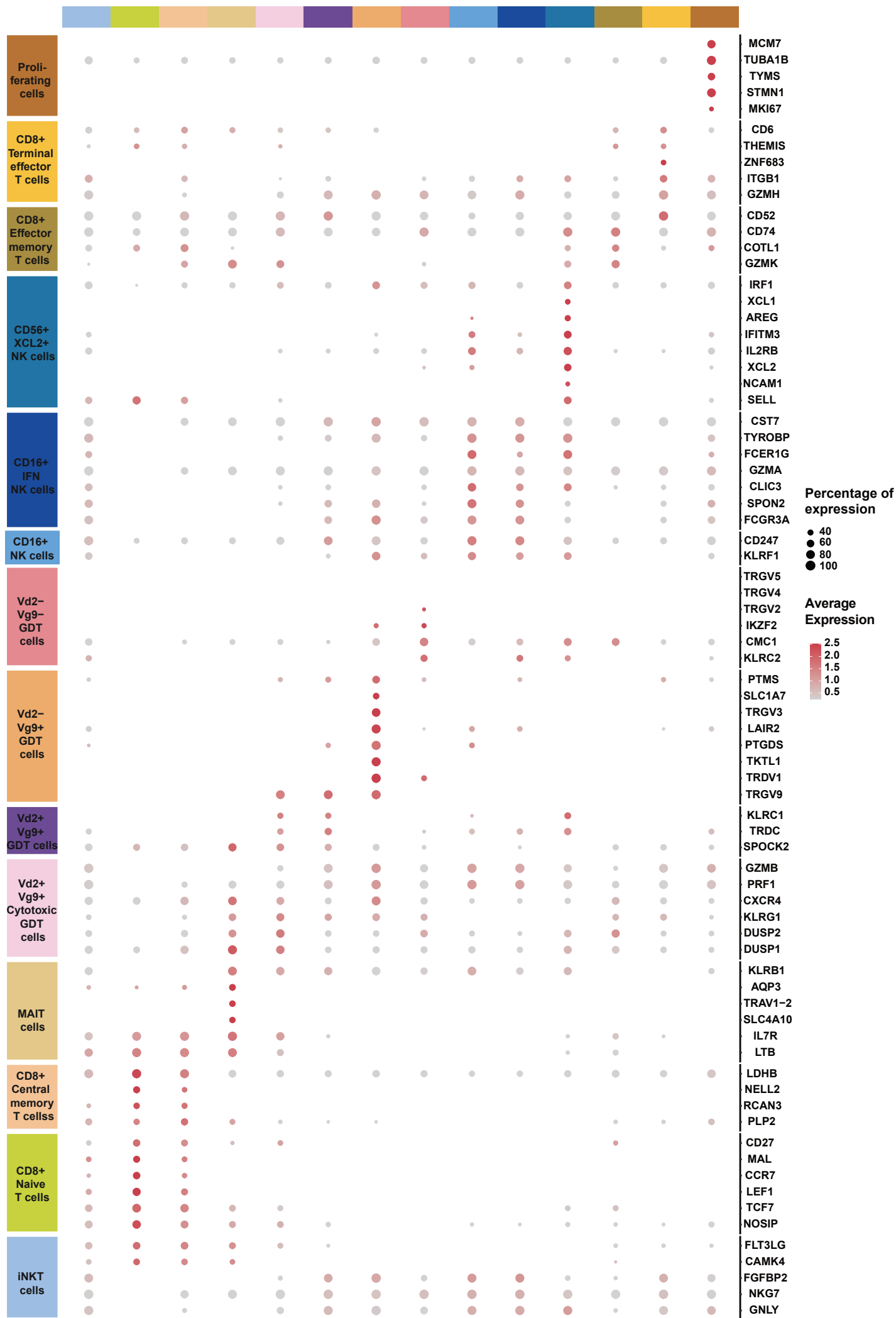

Supplement: Supplementary file 3 — Supporting Figure 3. Dot plot of gene markers for NK cells and T lymphocytes. [file JMV-97-e70335-s027.pdf]

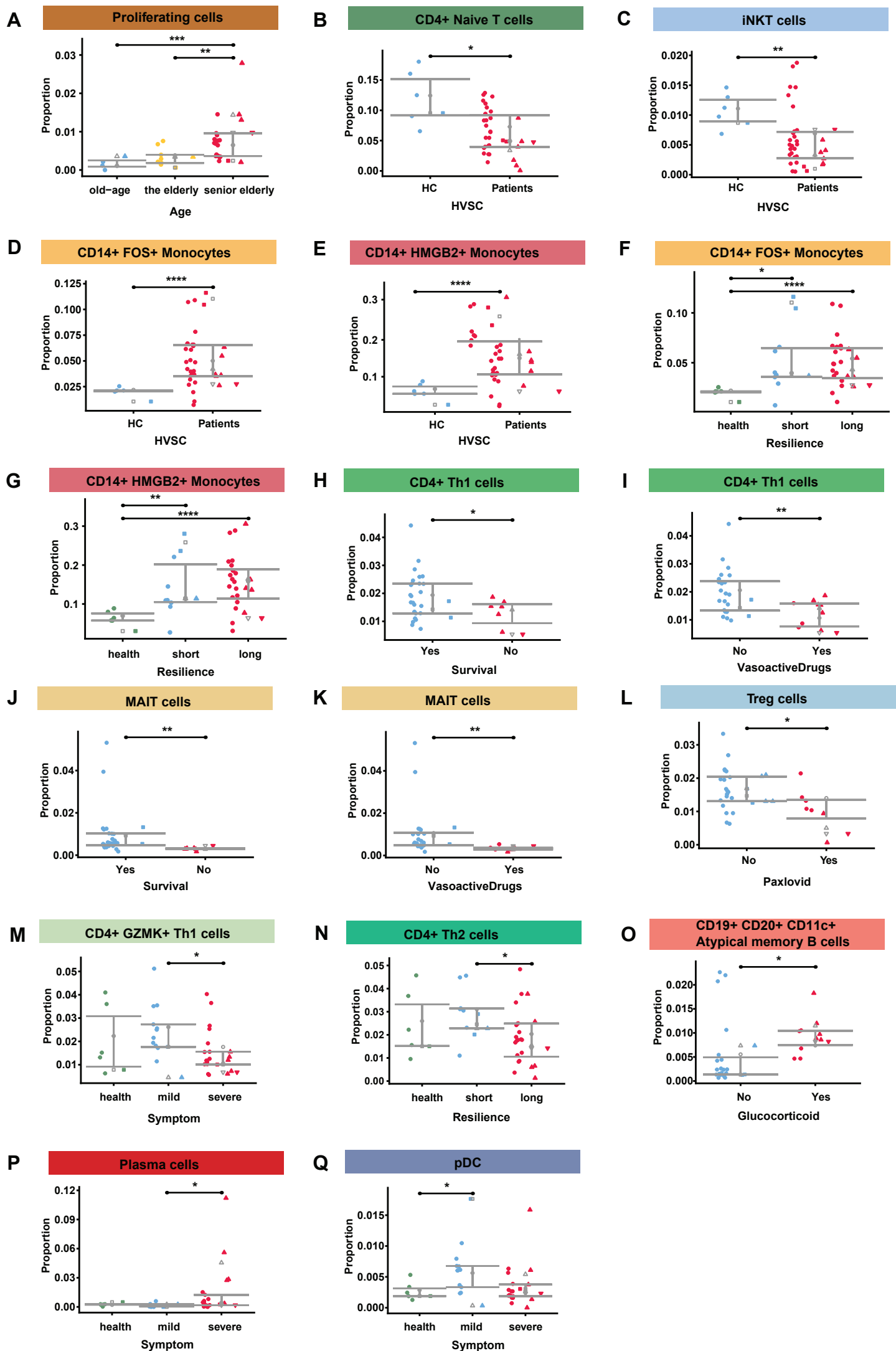

Supplement: Supplementary file 5 — Supporting Figure 5. Analysis of cell subpopulation proportions under different clinical indications. [file JMV-97-e70335-s014.pdf]

A

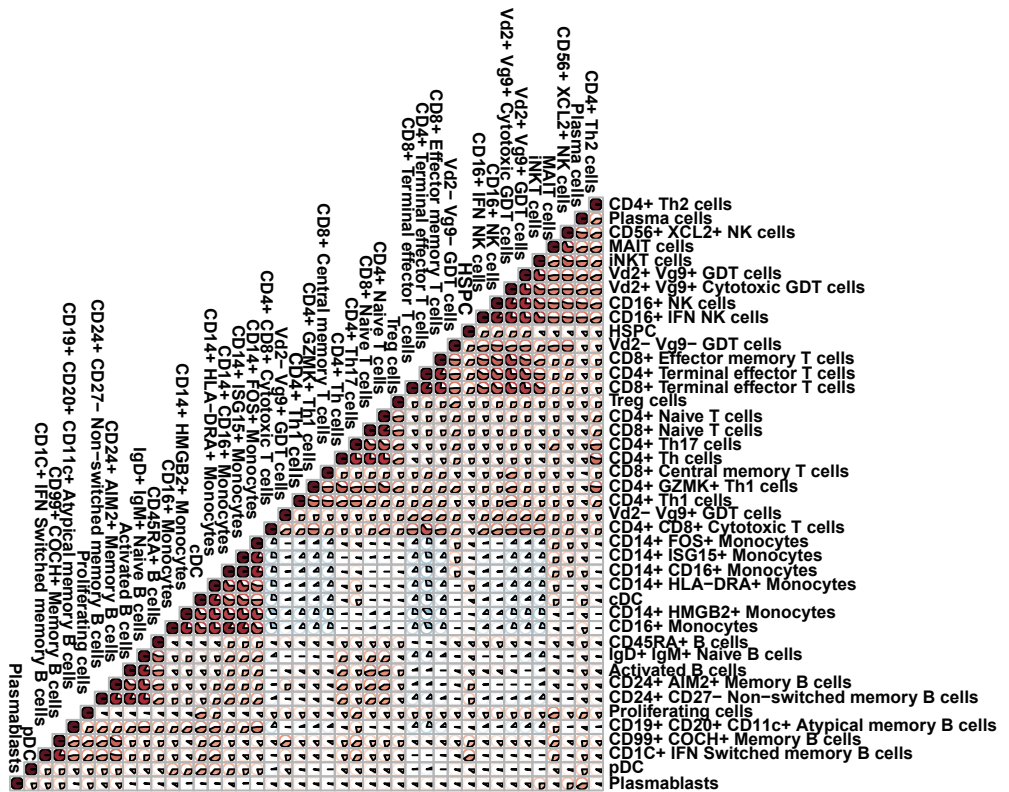

B

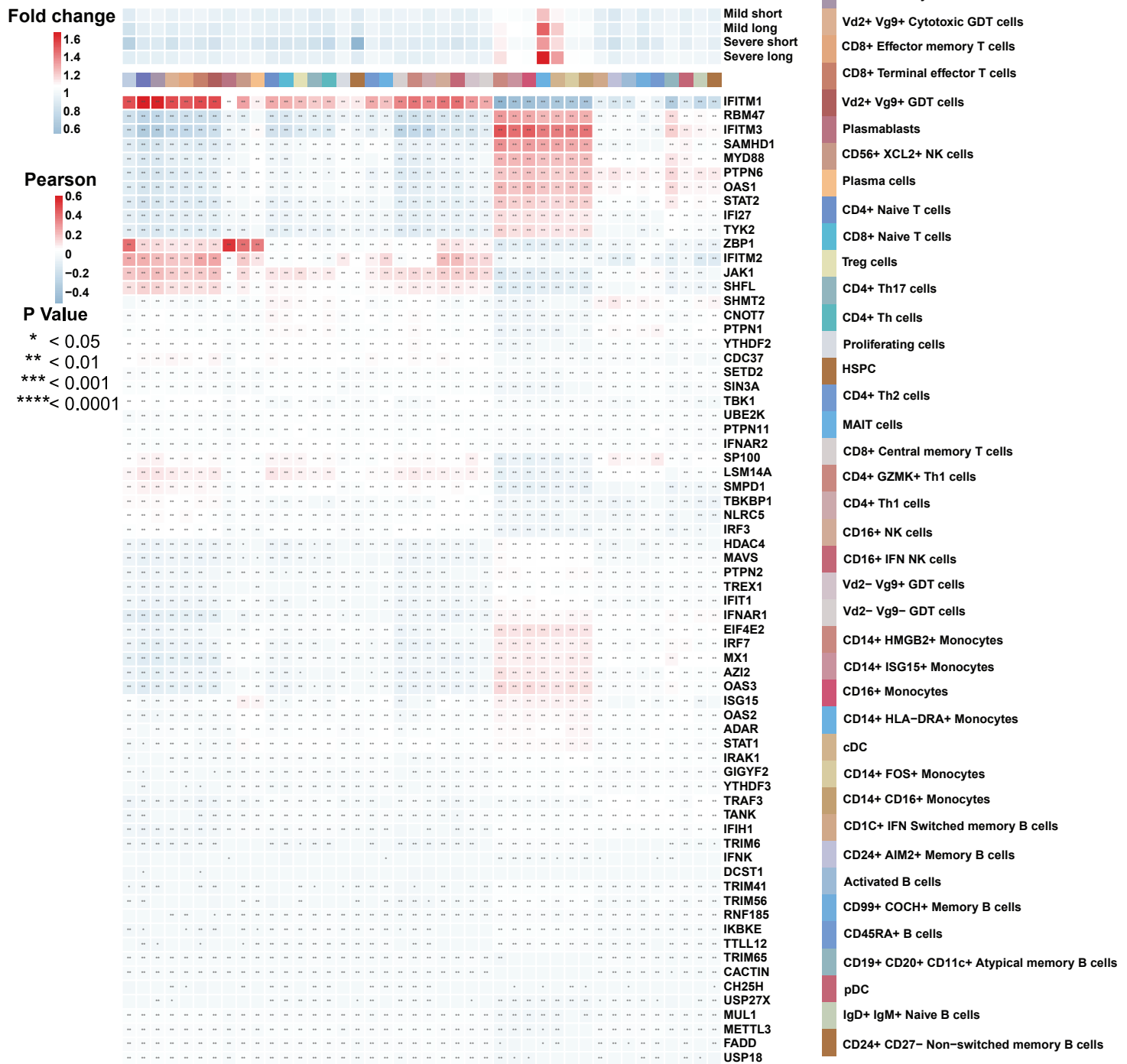

Supplement: Supplementary file 6 — Supporting Figure 6. Immune cell infiltration analysis and correlation analysis of ISGs gene set scores. [file JMV-97-e70335-s010.pdf]

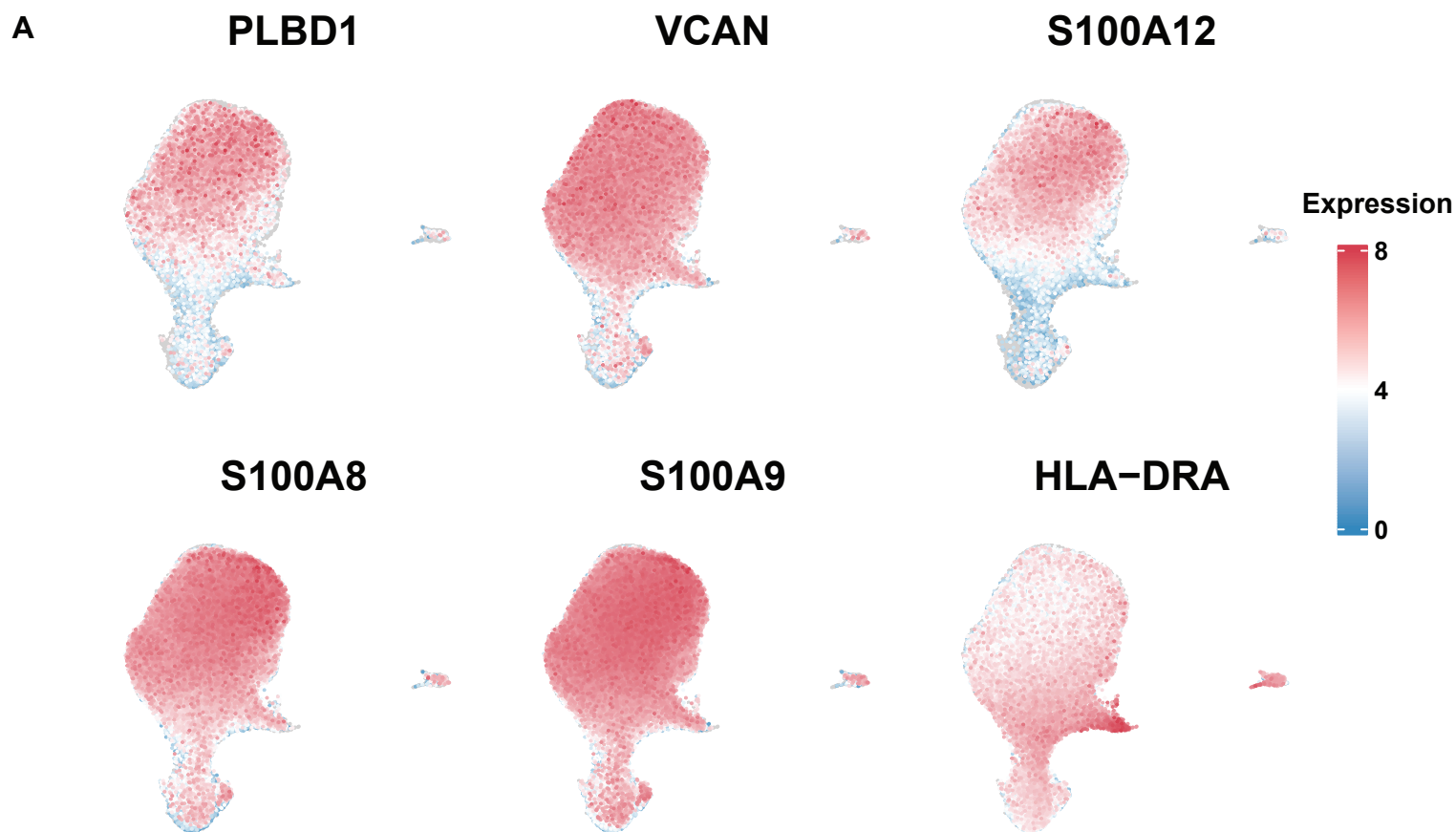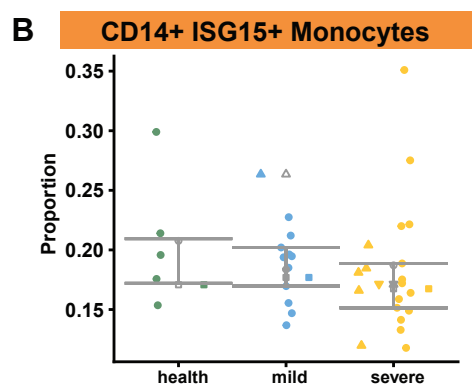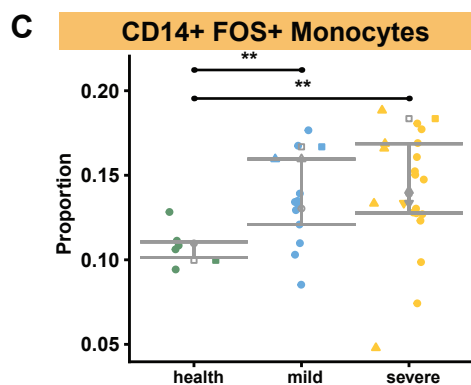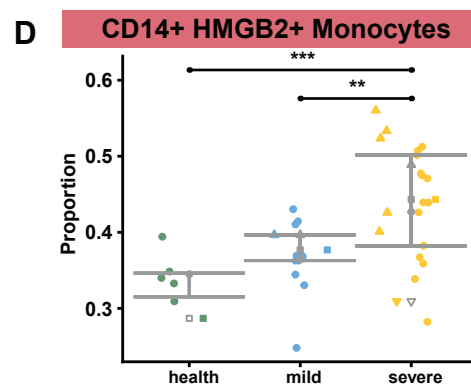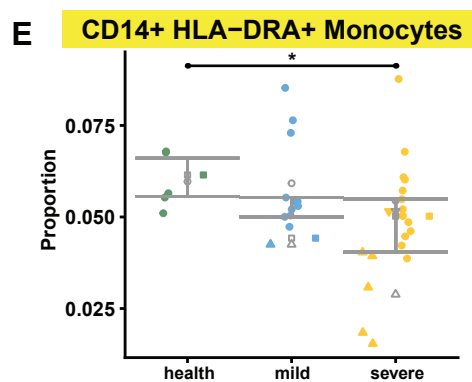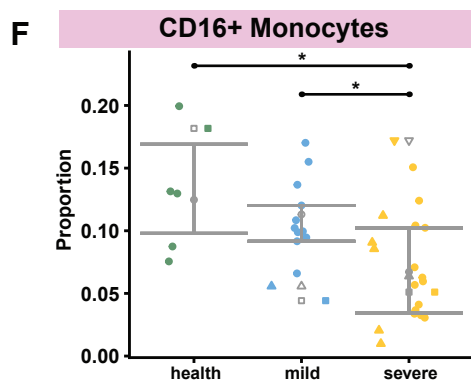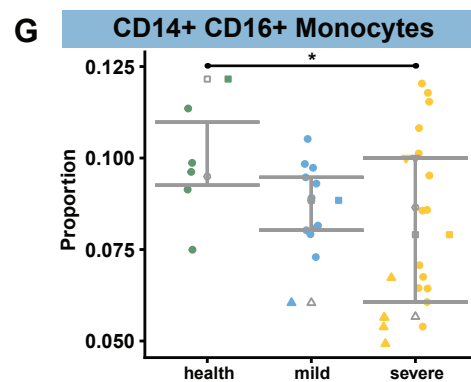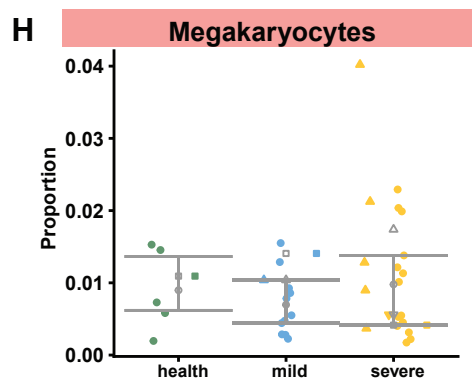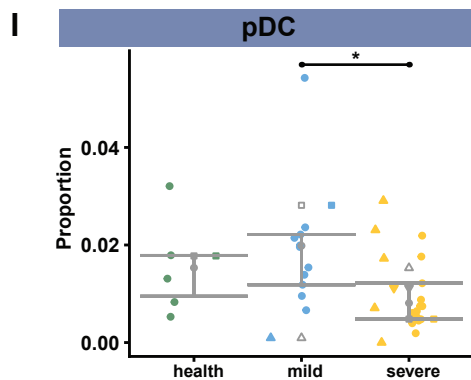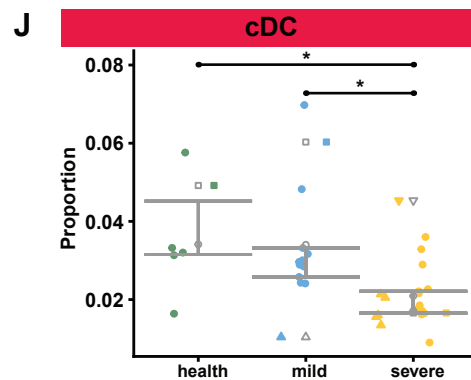

Supplement: Supplementary file 7 — Supporting Figure 7. Relative cell proportions of myeloid cells under varying disease severities. [file JMV-97-e70335-s029.pdf]

**A**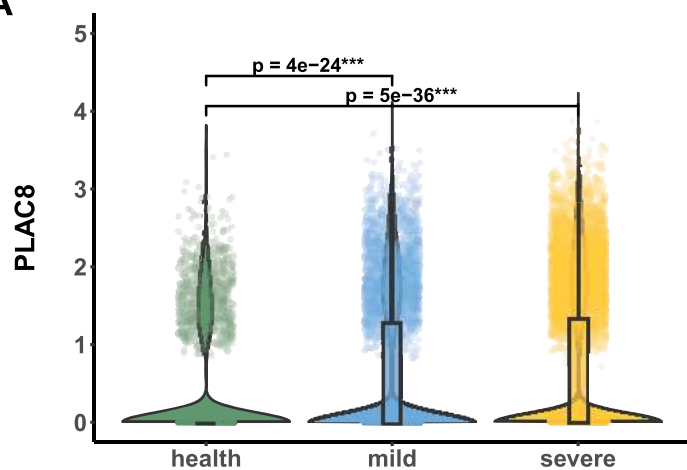**B**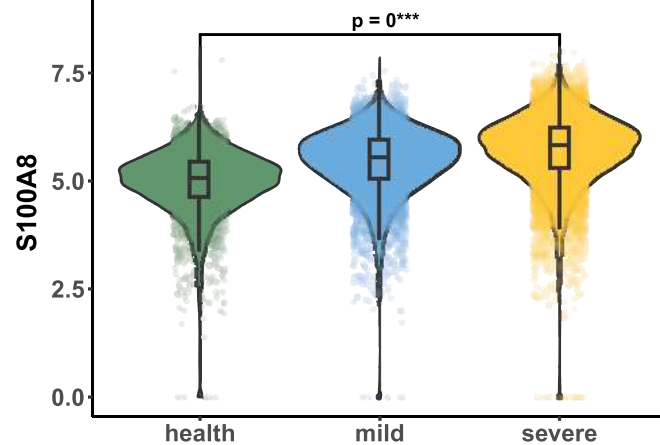**C**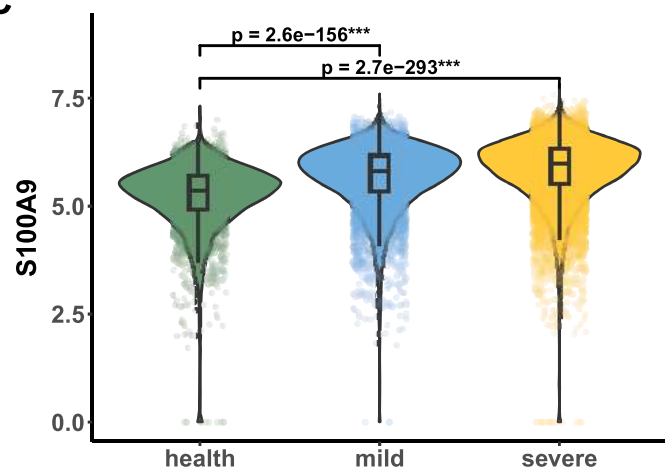**D**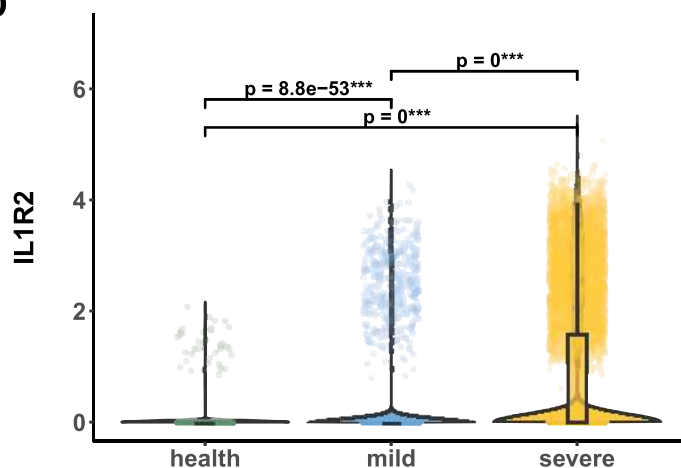**E**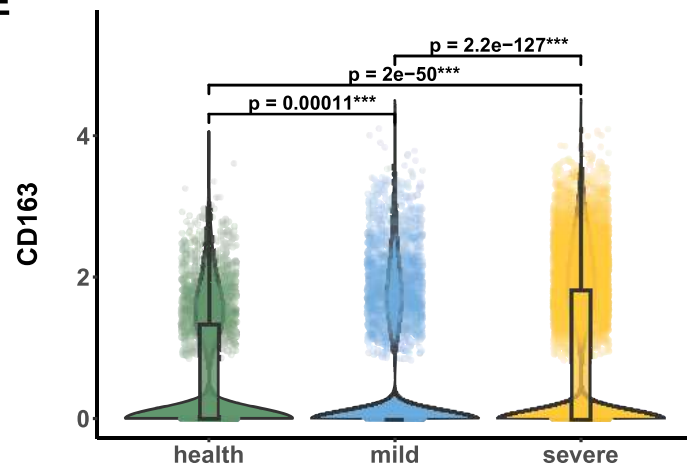**F**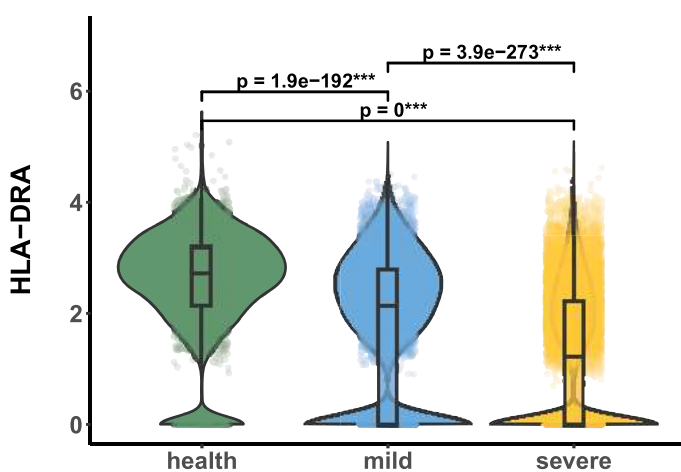**G**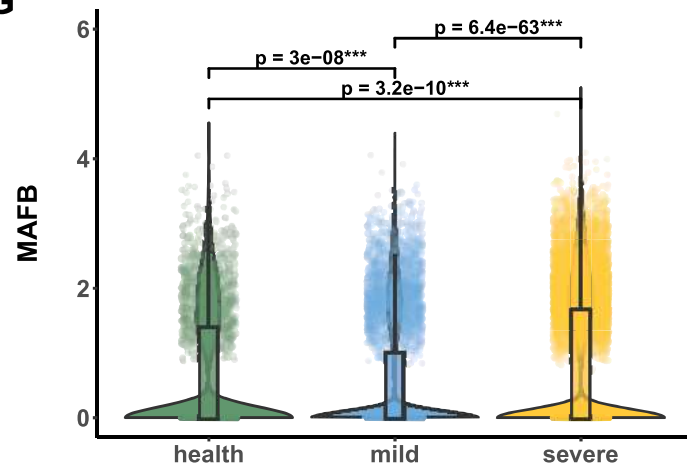

Supplement: Supplementary file 8 — Supporting Figure 8. Violin plots of MDSC‐like marker expression in CD14+ HMGB2+ monocytes across symptom severity groups. [file JMV-97-e70335-s026.pdf]

A

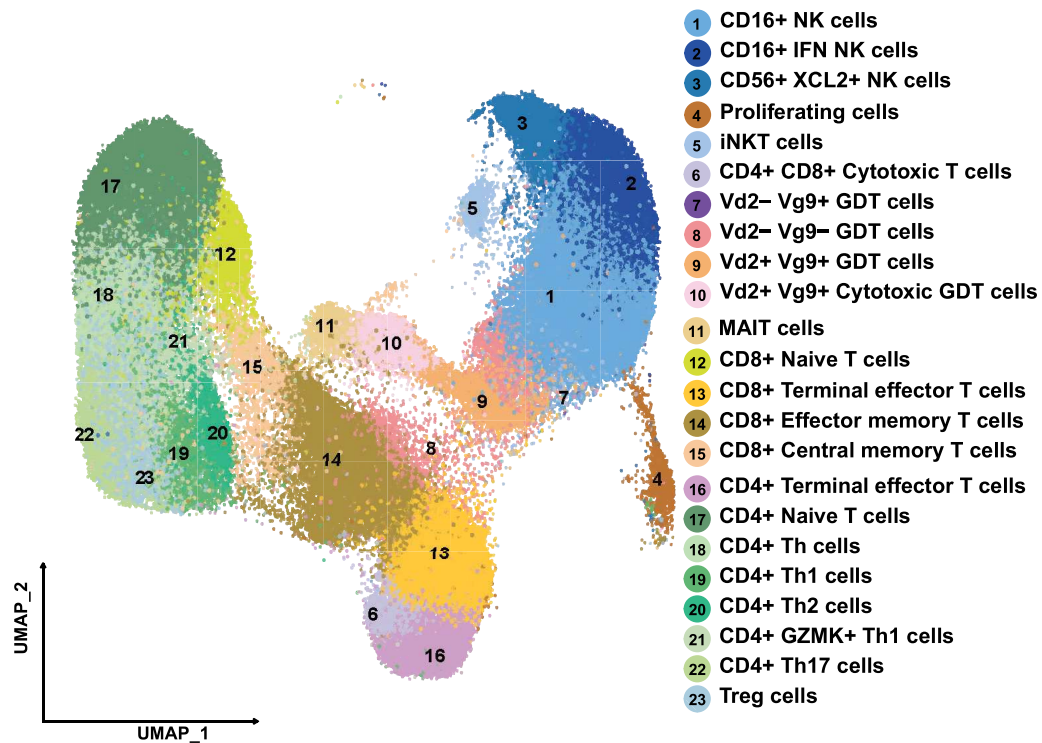

B

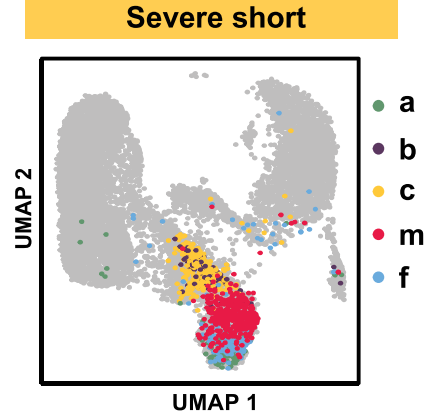

C

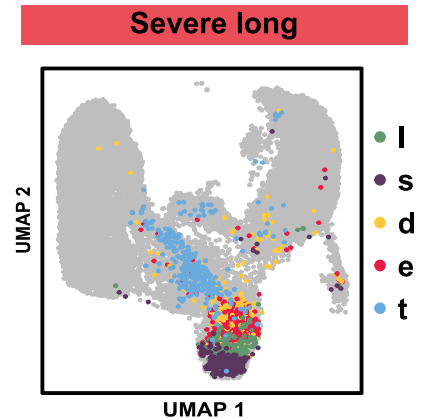

D

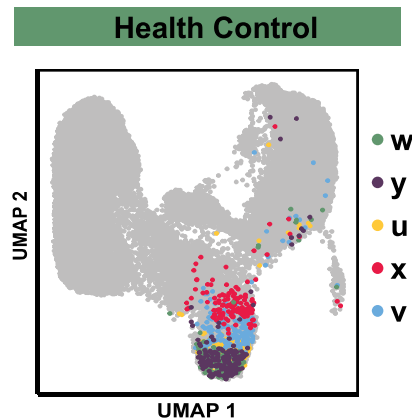

E

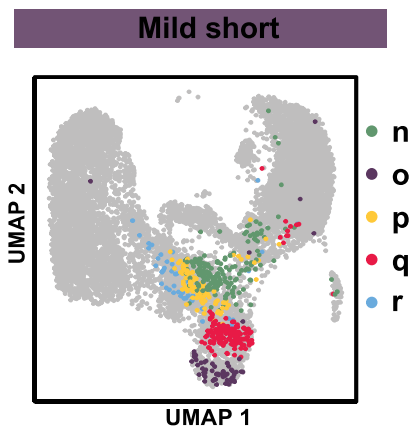

F

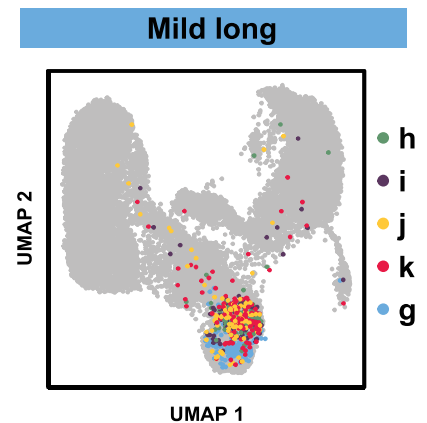

G

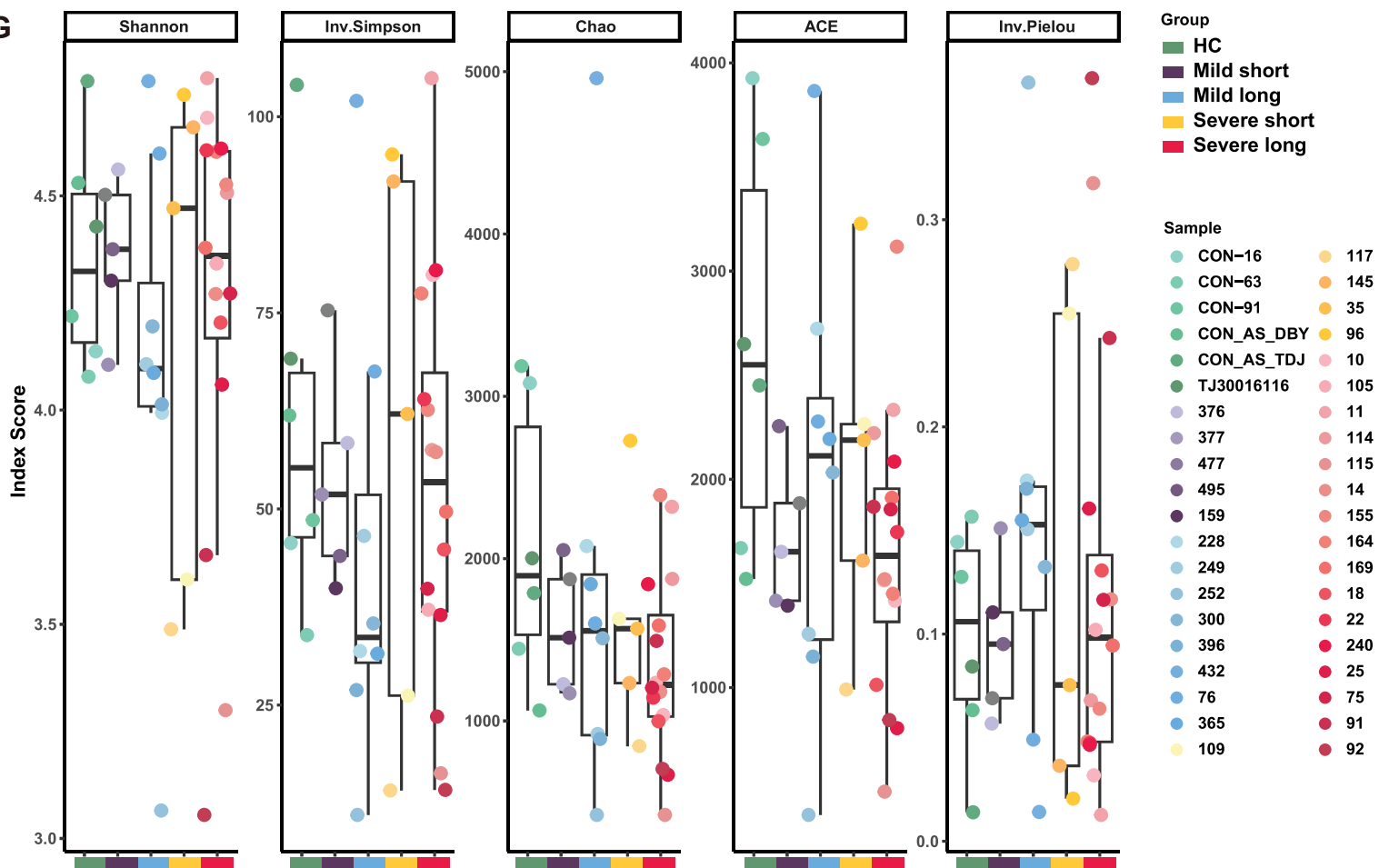

Supplement: Supplementary file 11 — Supporting Figure 11. UMAP visualization of T lymphocytes, NK cells, and TCR clonotype distributions. [file JMV-97-e70335-s013.pdf]

A

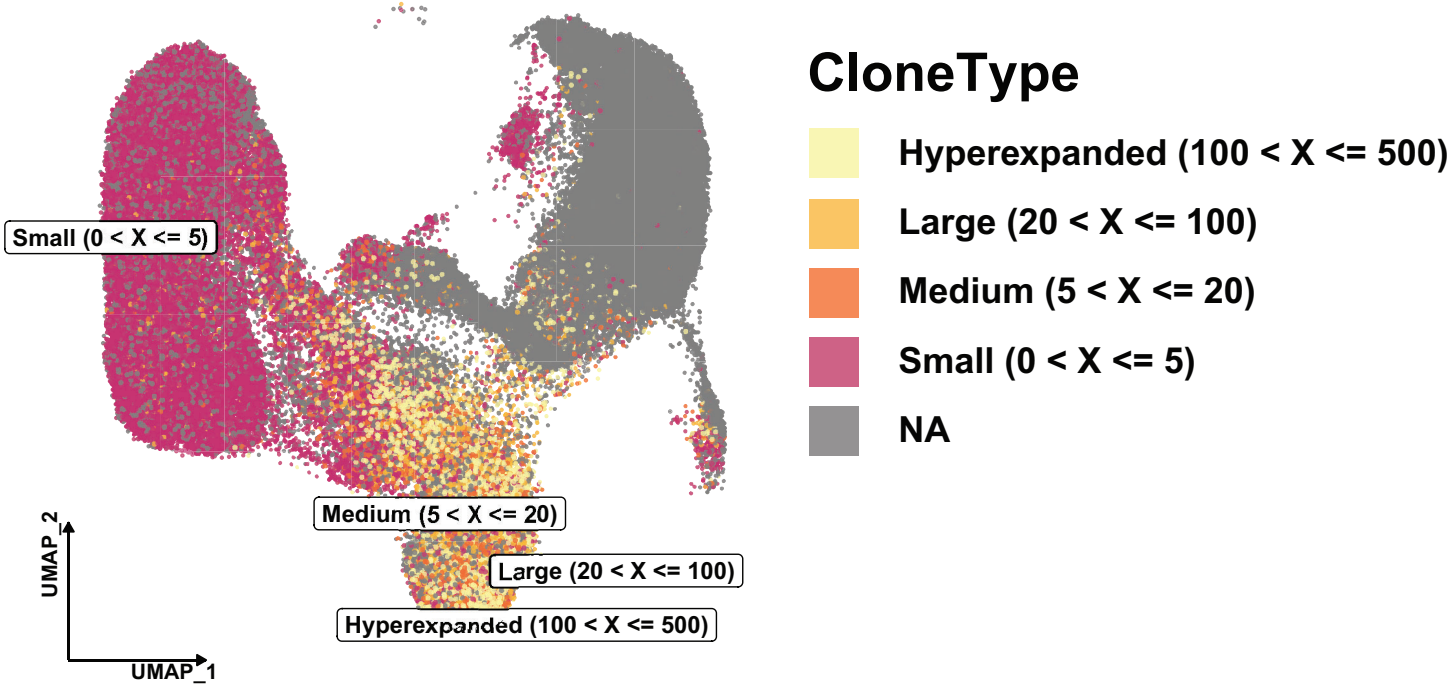

B

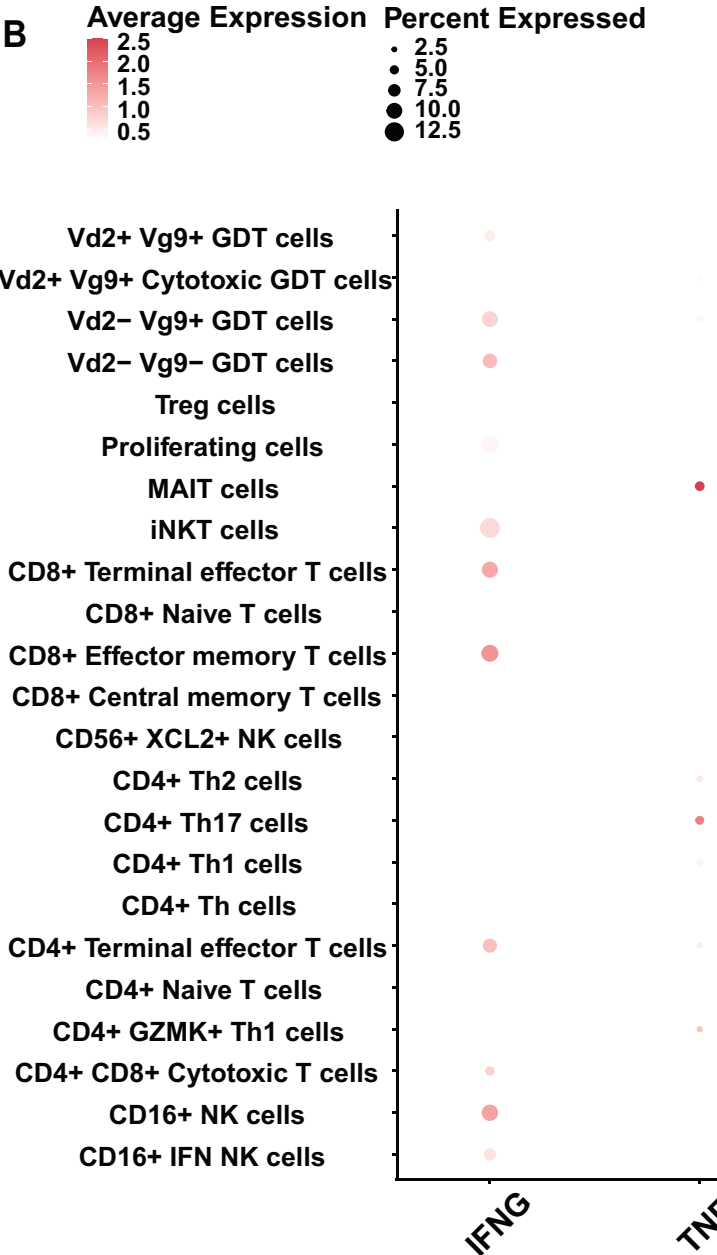

C

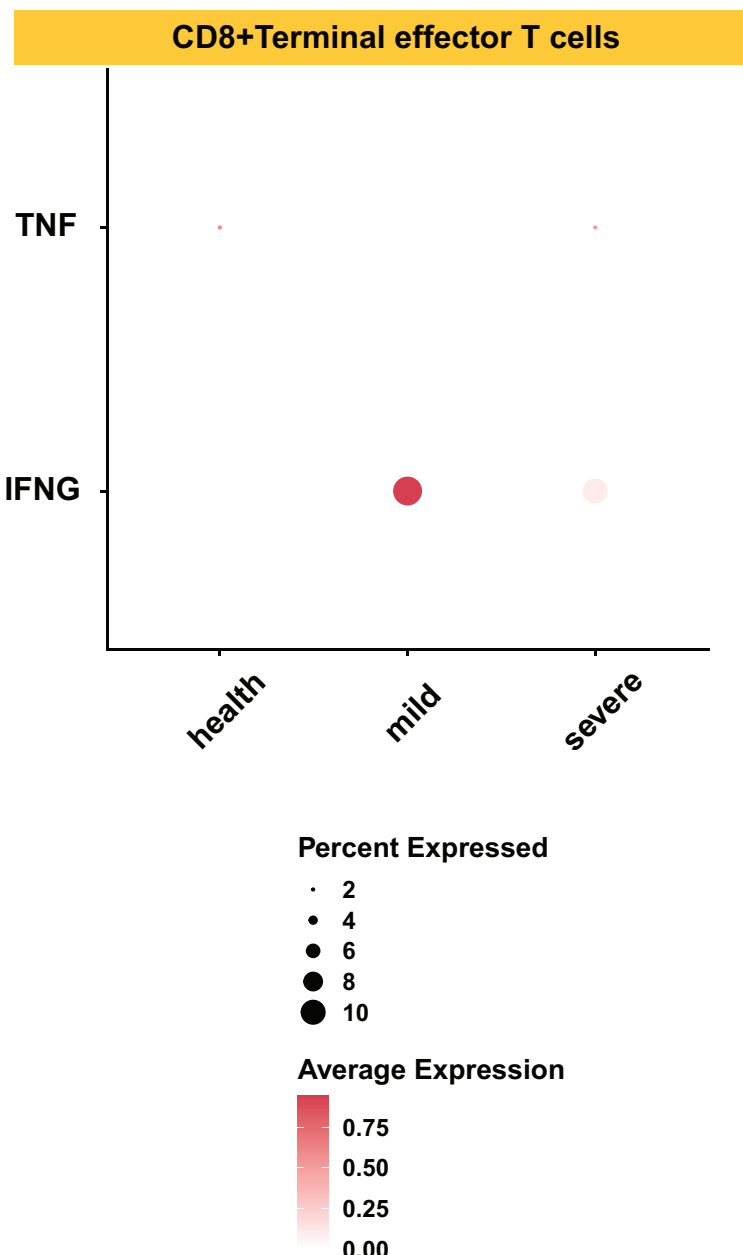

Supplement: Supplementary file 12 — Supporting Figure 12. Clone expanded distribution and gene expression dot plots of T lymphocytes and NK cells. [file JMV-97-e70335-s018.pdf]

A

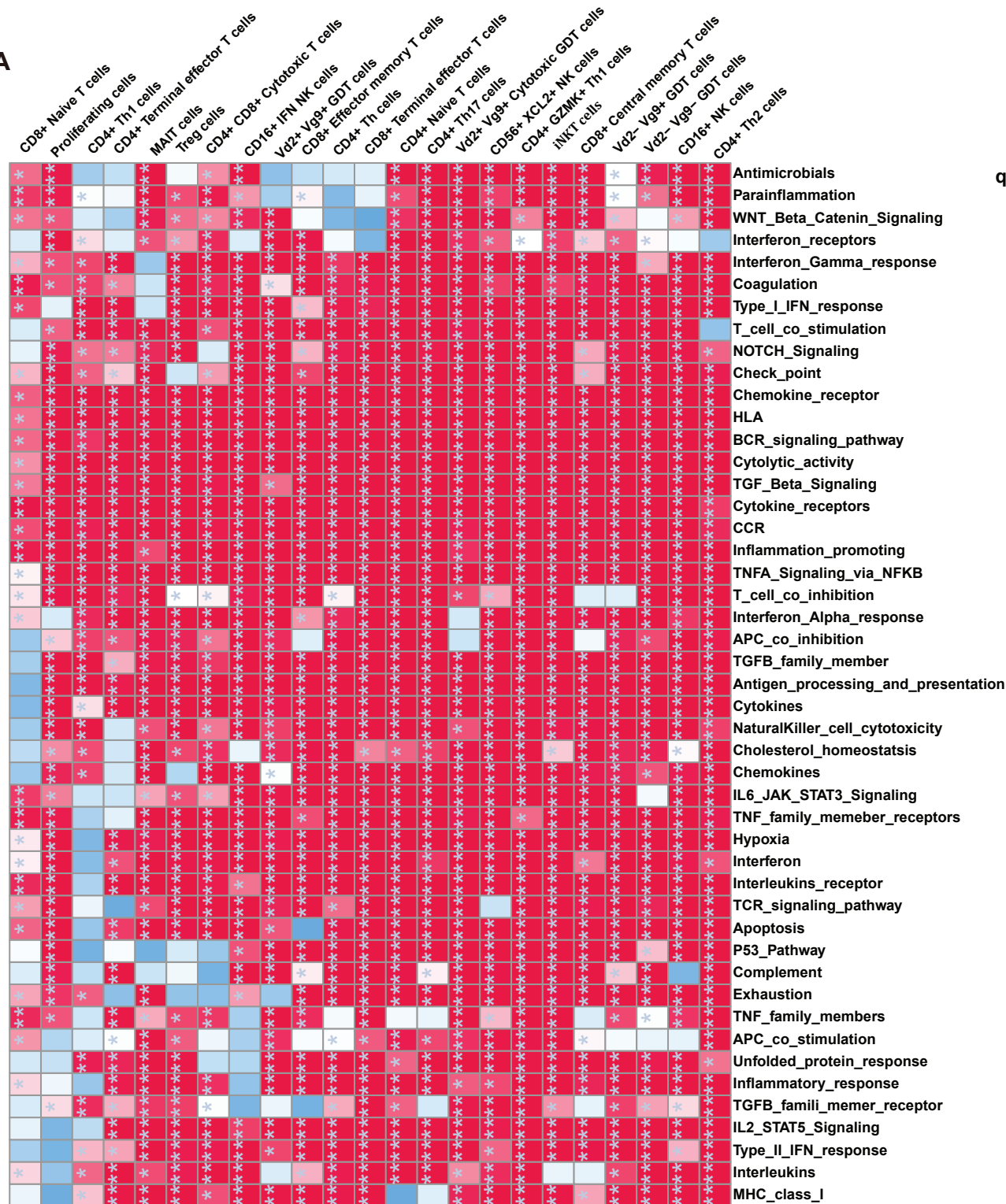

B

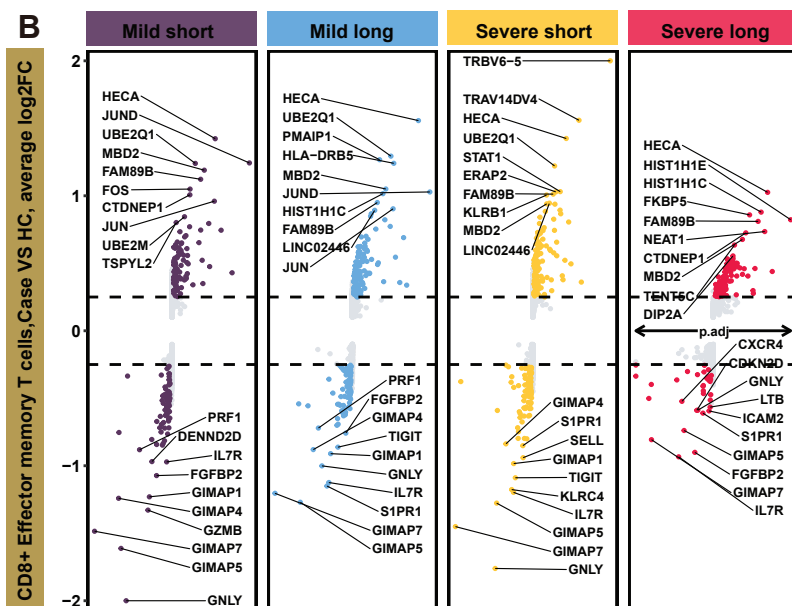

C

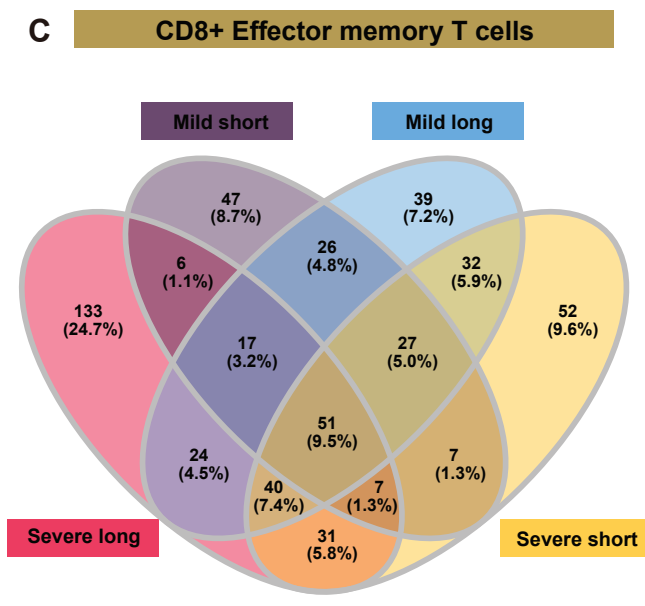

Supplement: Supplementary file 13 — Supporting Figure 13. Immunogenomic analysis and gene set scoring significance for T lymphocytes and NK cells. [file JMV-97-e70335-s004.pdf]

A

CD8+ Effector memory T cells

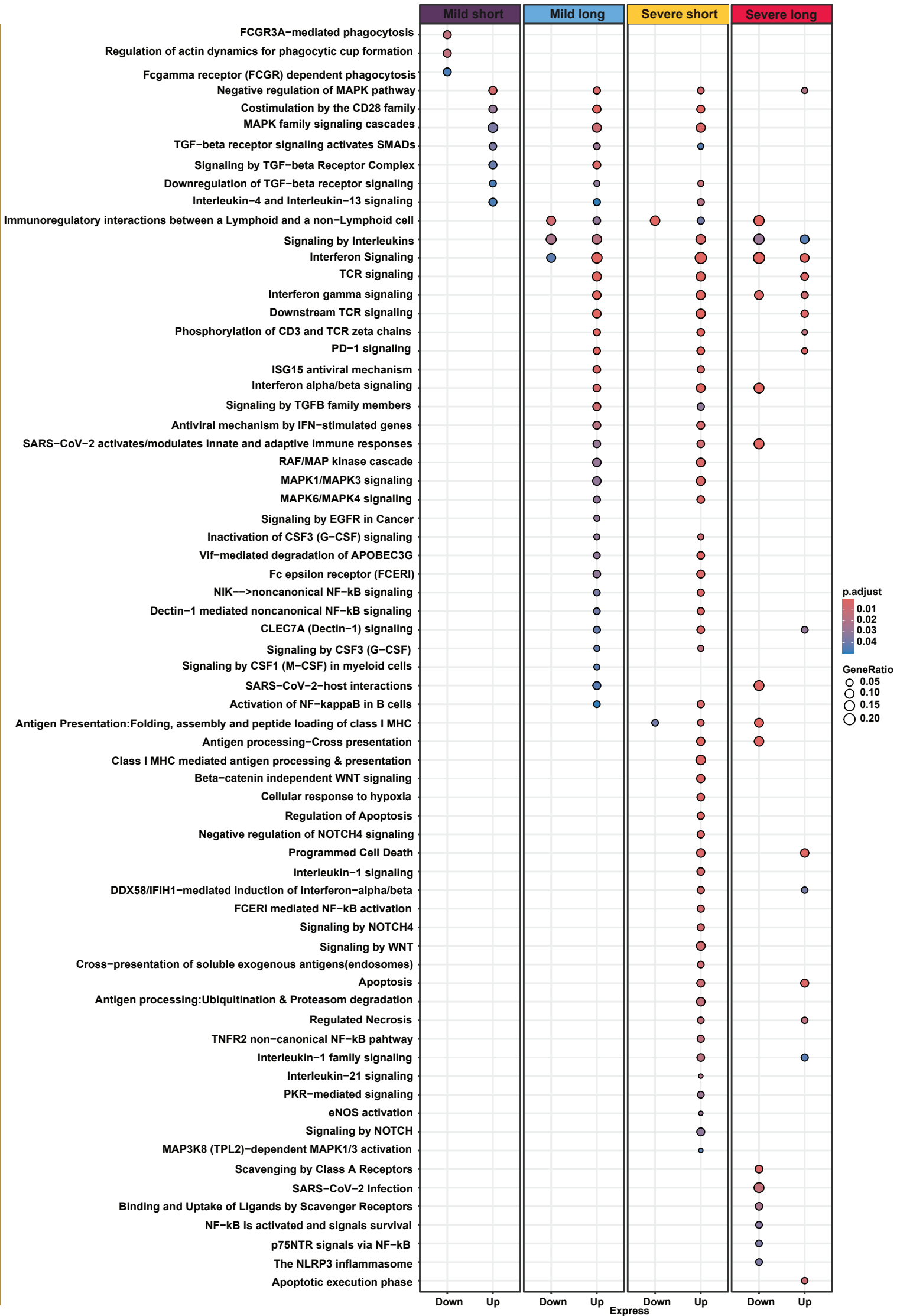

Supplement: Supplementary file 14 — Supporting Figure 14. Pathway enrichment visualization of differential genes in CD8+ effector memory T cells across different disease groups. [file JMV-97-e70335-s007.pdf]

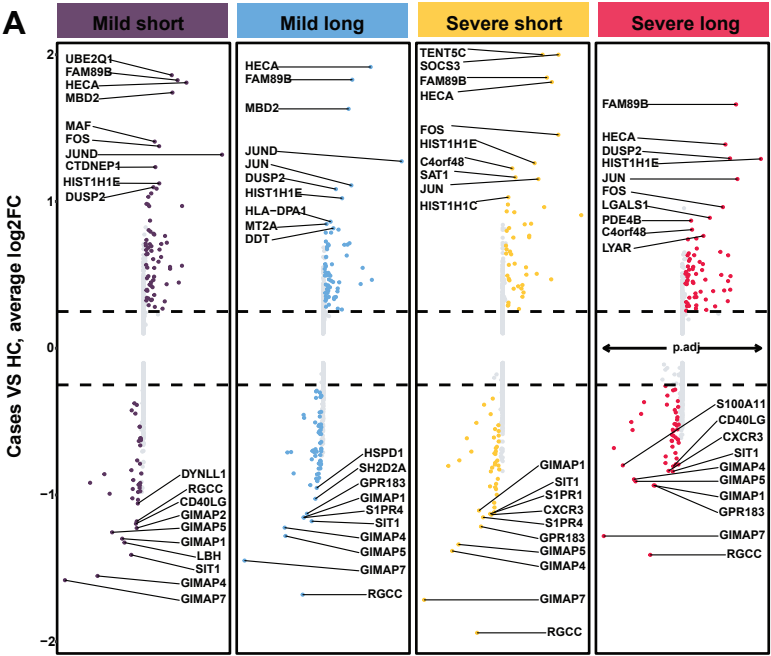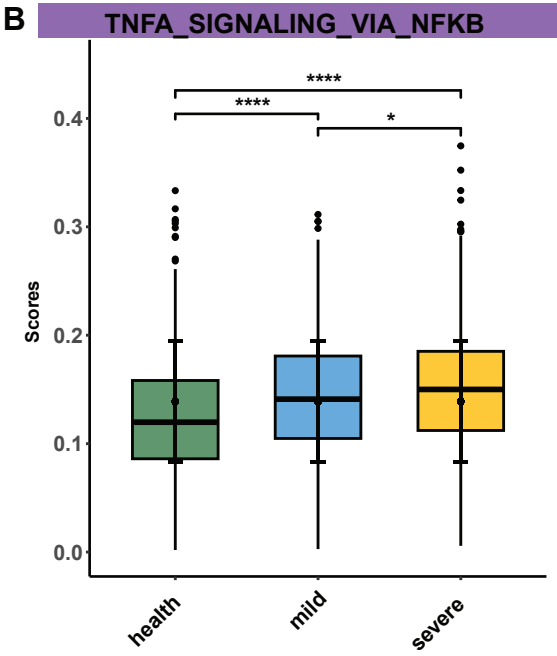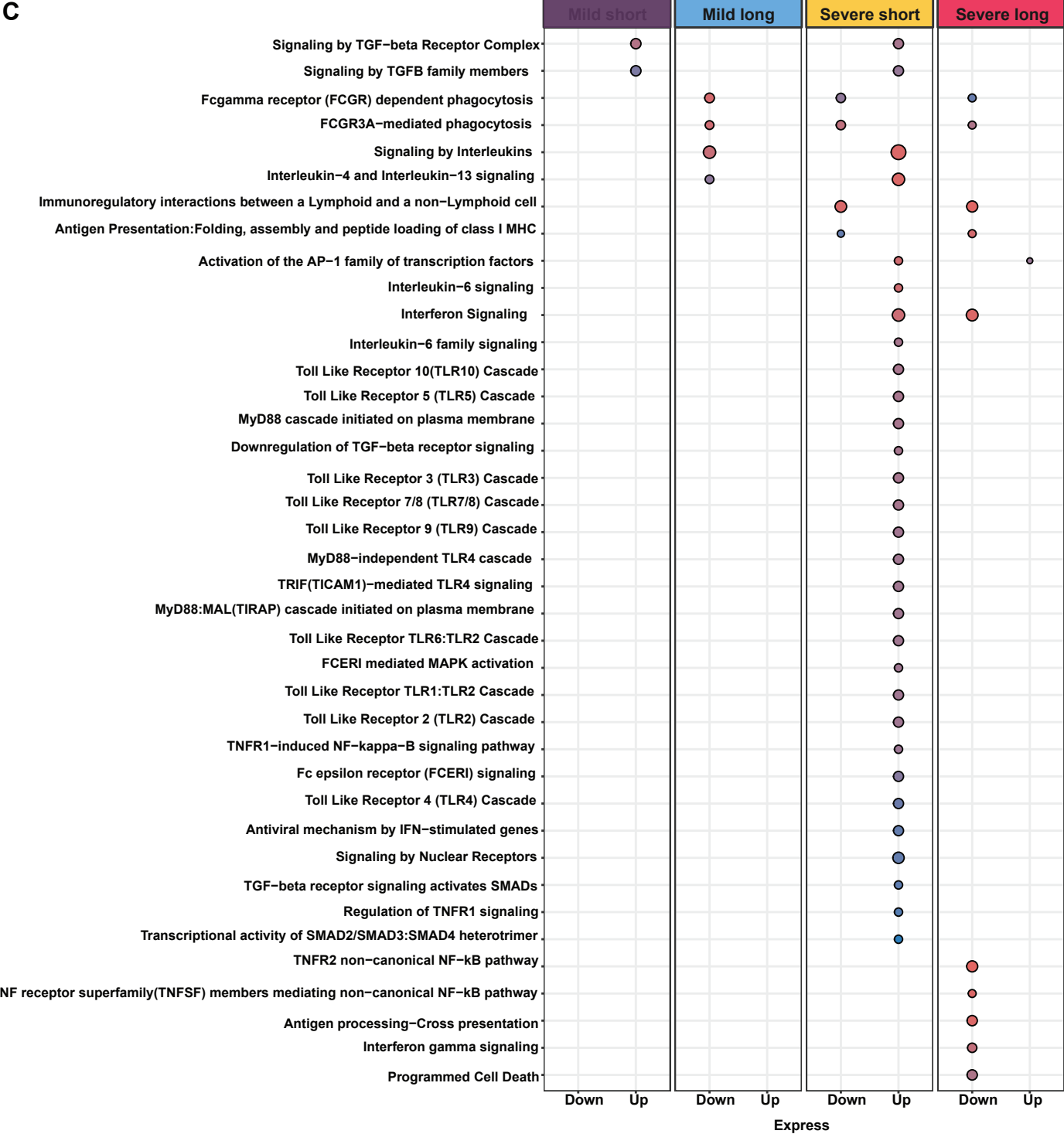

Supplement: Supplementary file 15 — Supporting Figure 15. Differential analysis of CD4+ GZMK+ Th1 cells across various groups. [file JMV-97-e70335-s008.pdf]

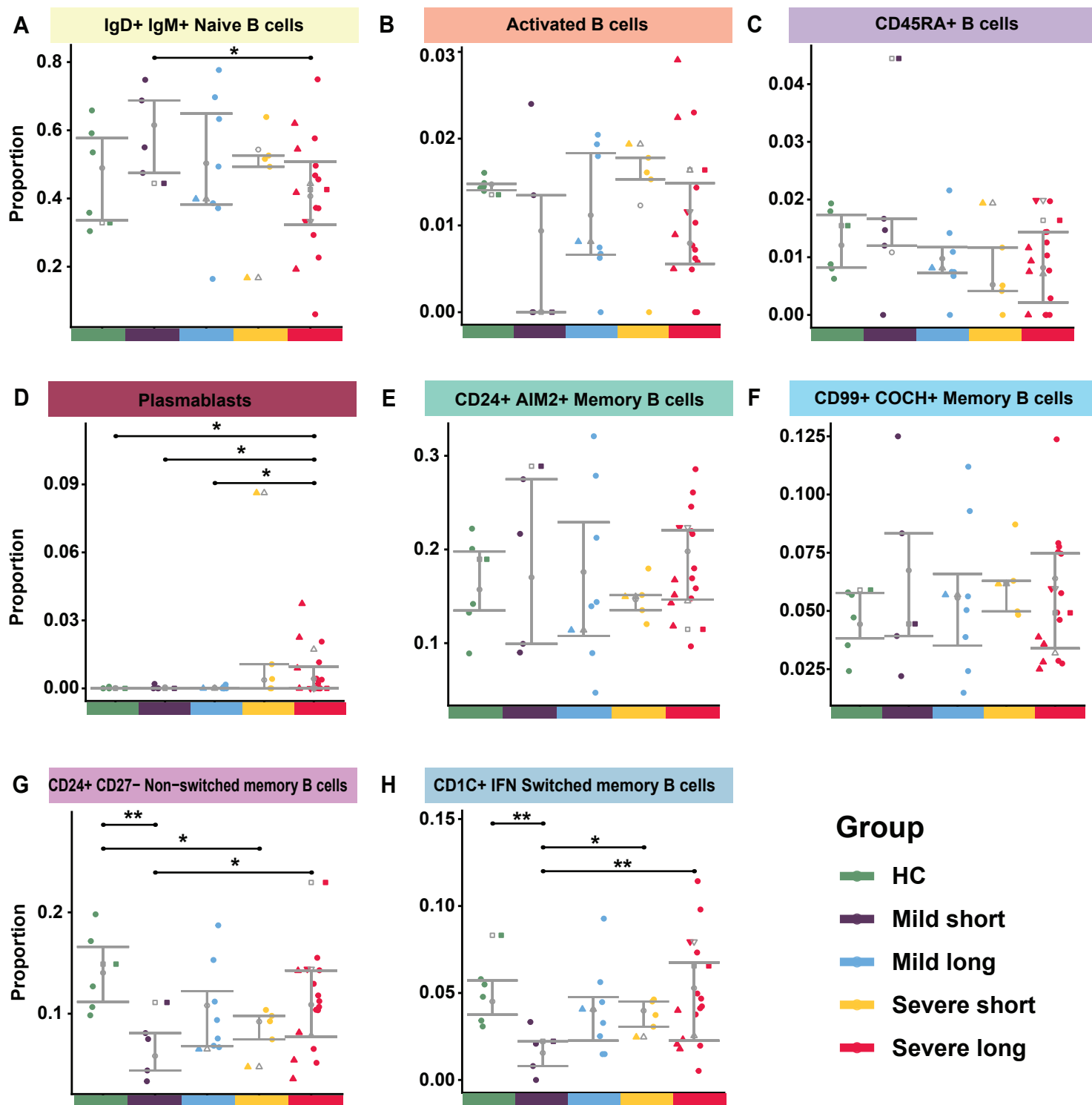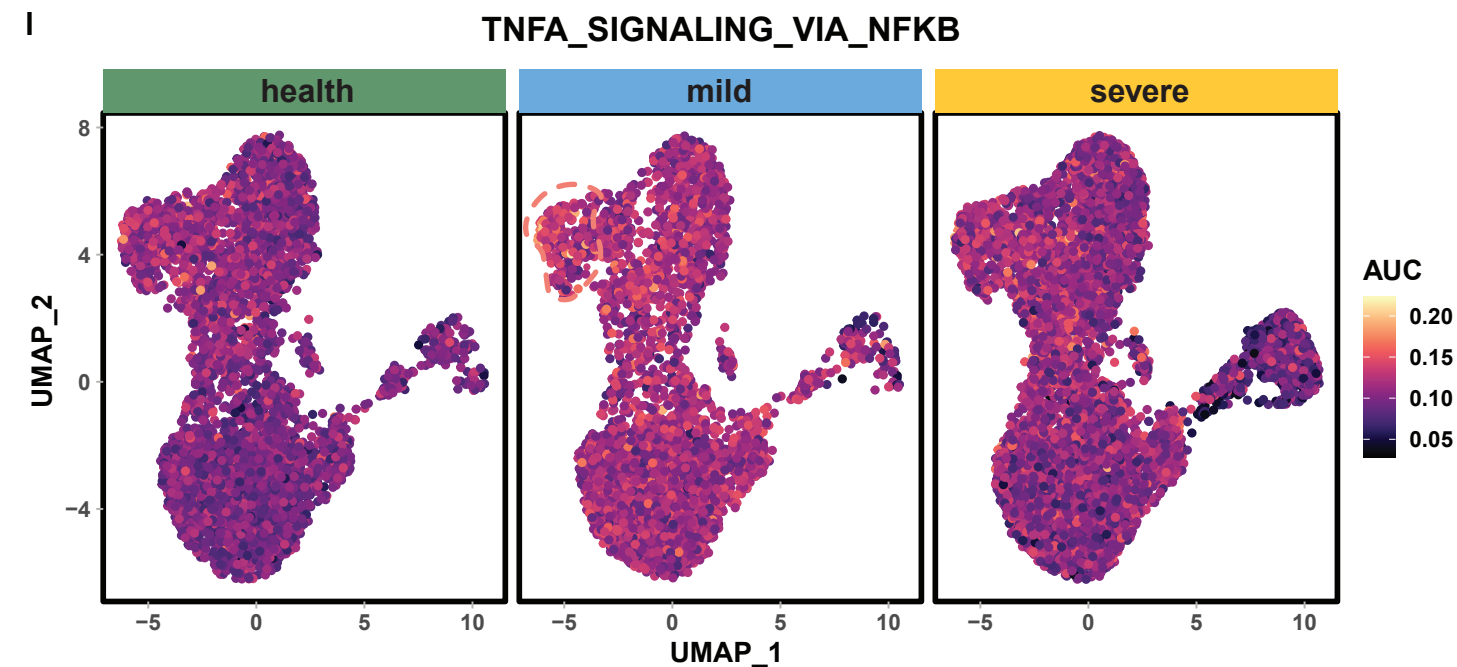

Supplement: Supplementary file 16 — Supporting Figure 16. Composition analysis and pathway enrichment scores of B cells. [file JMV-97-e70335-s021.pdf]

A Plasma cells

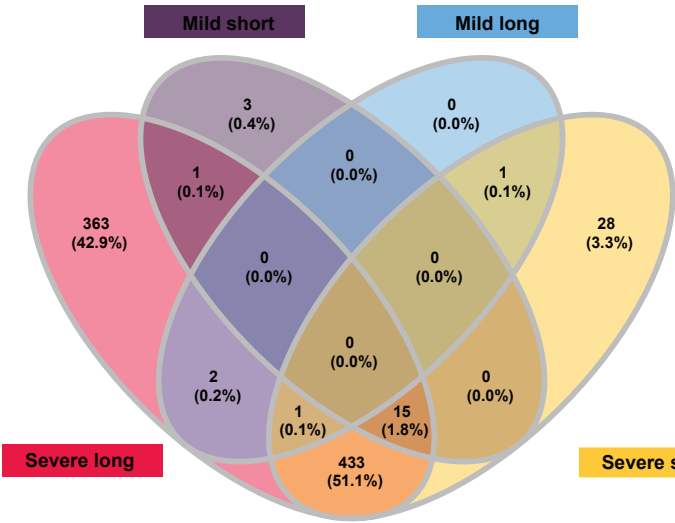

B CD19+ CD20+ CD11c+ Atypical memory B cells

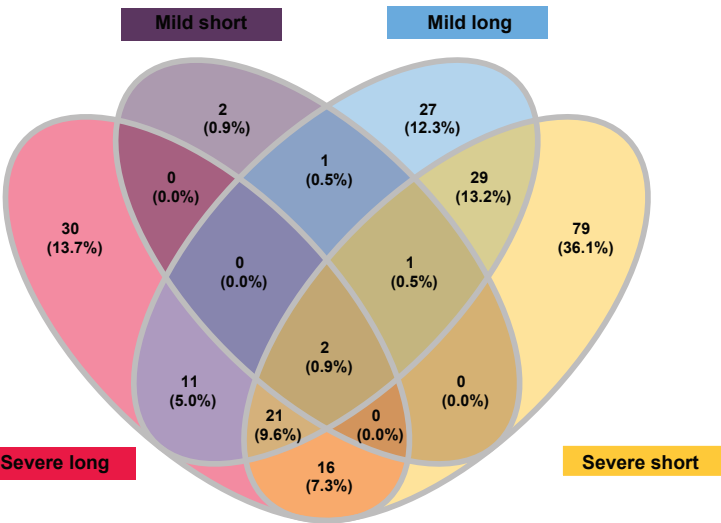

C

Dotplot of Enriched KEGG Pathways

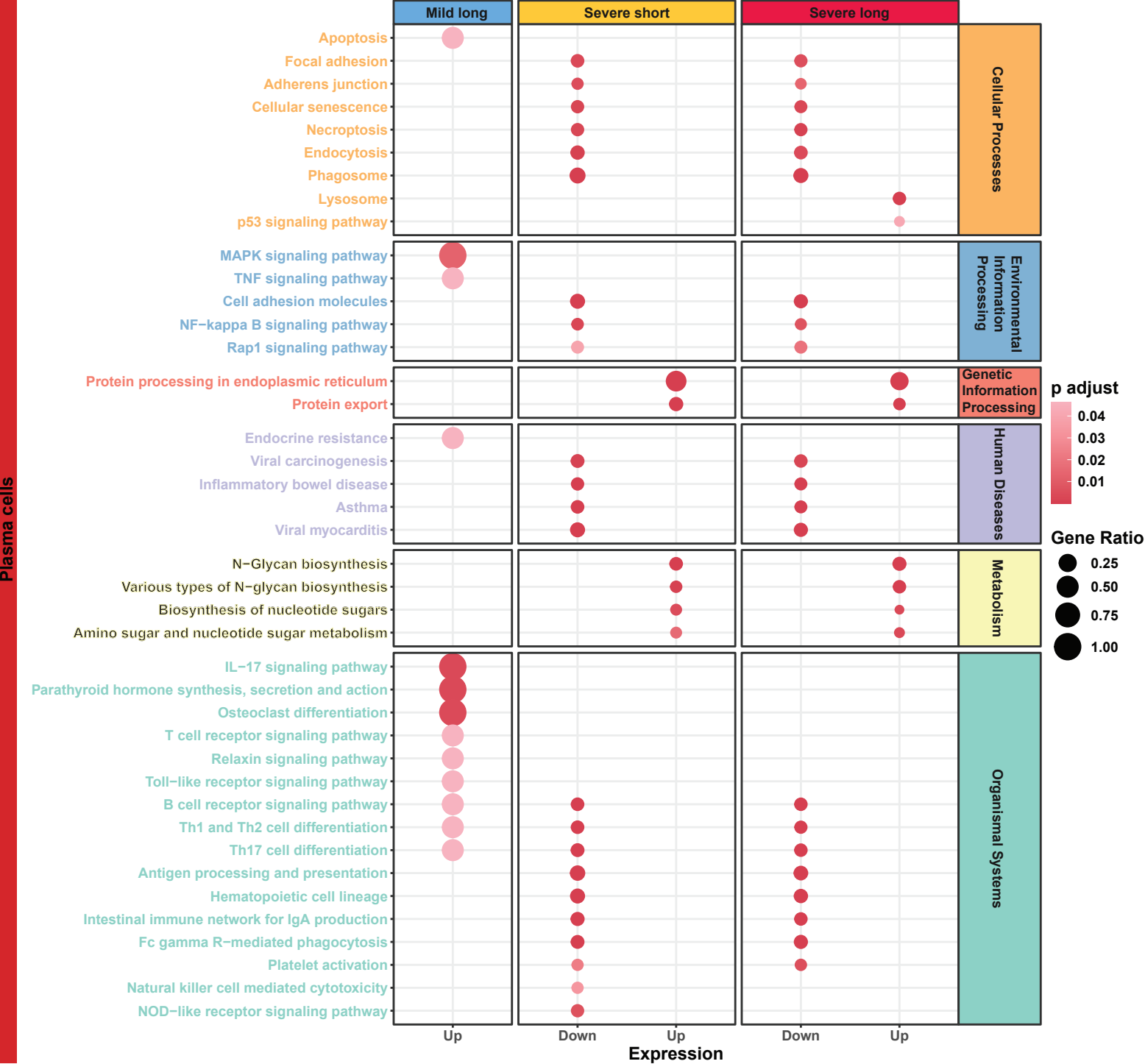

Supplement: Supplementary file 18 — Supporting Figure 18. Differential gene analysis of plasma cells and CD19+ CD20+ CD11c+ atypical memory B cells. [file JMV-97-e70335-s020.pdf]

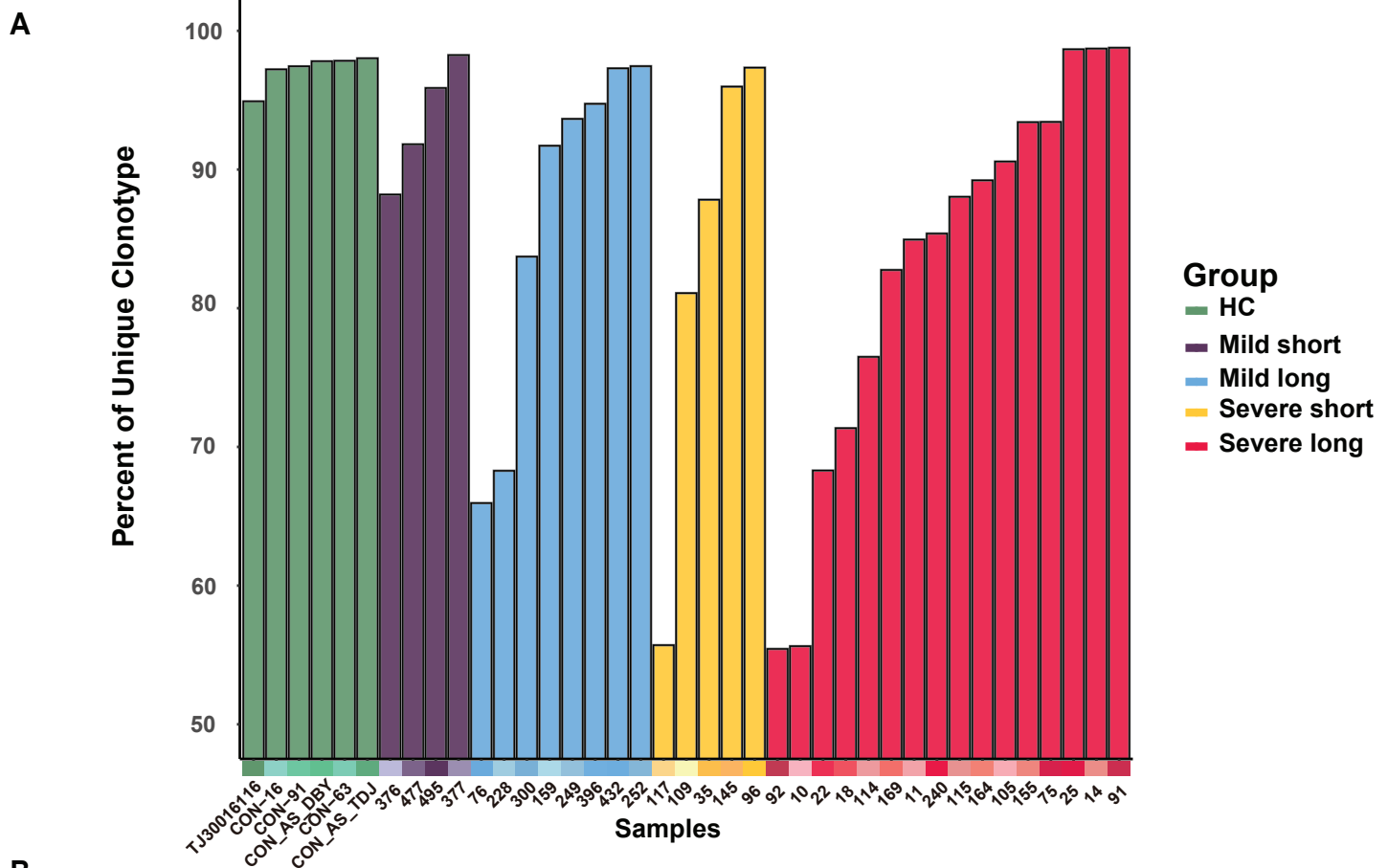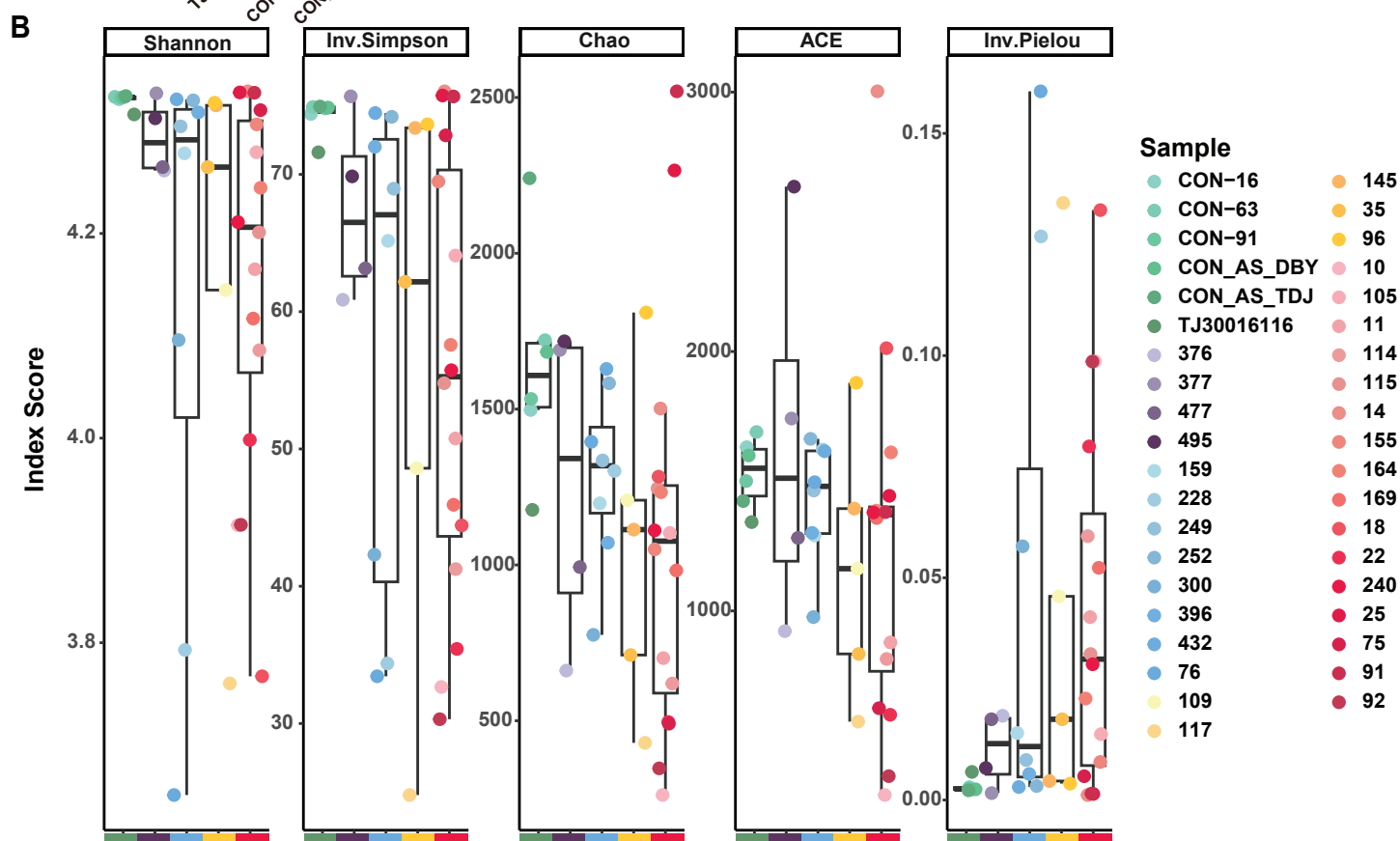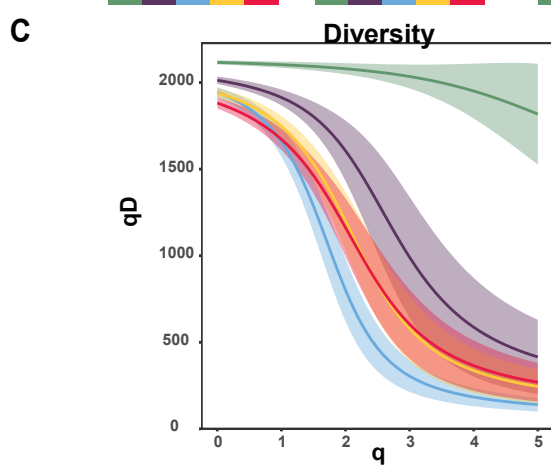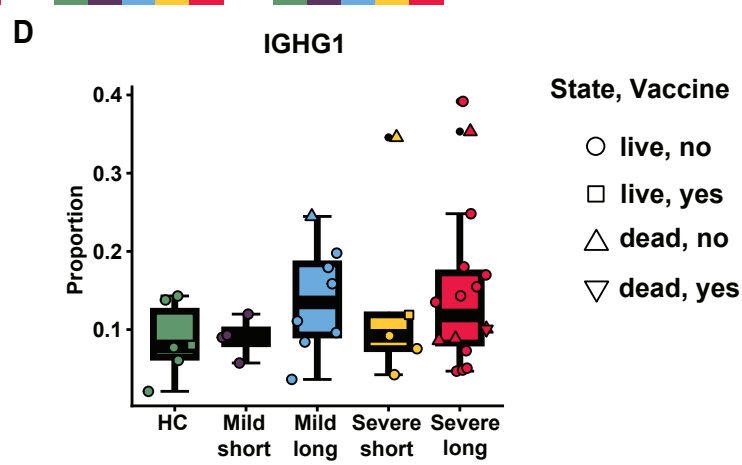

Supplement: Supplementary file 19 — Supporting Figure 19. BCR clonal status analysis and clonotype diversity assessment. [file JMV-97-e70335-s006.pdf]

A

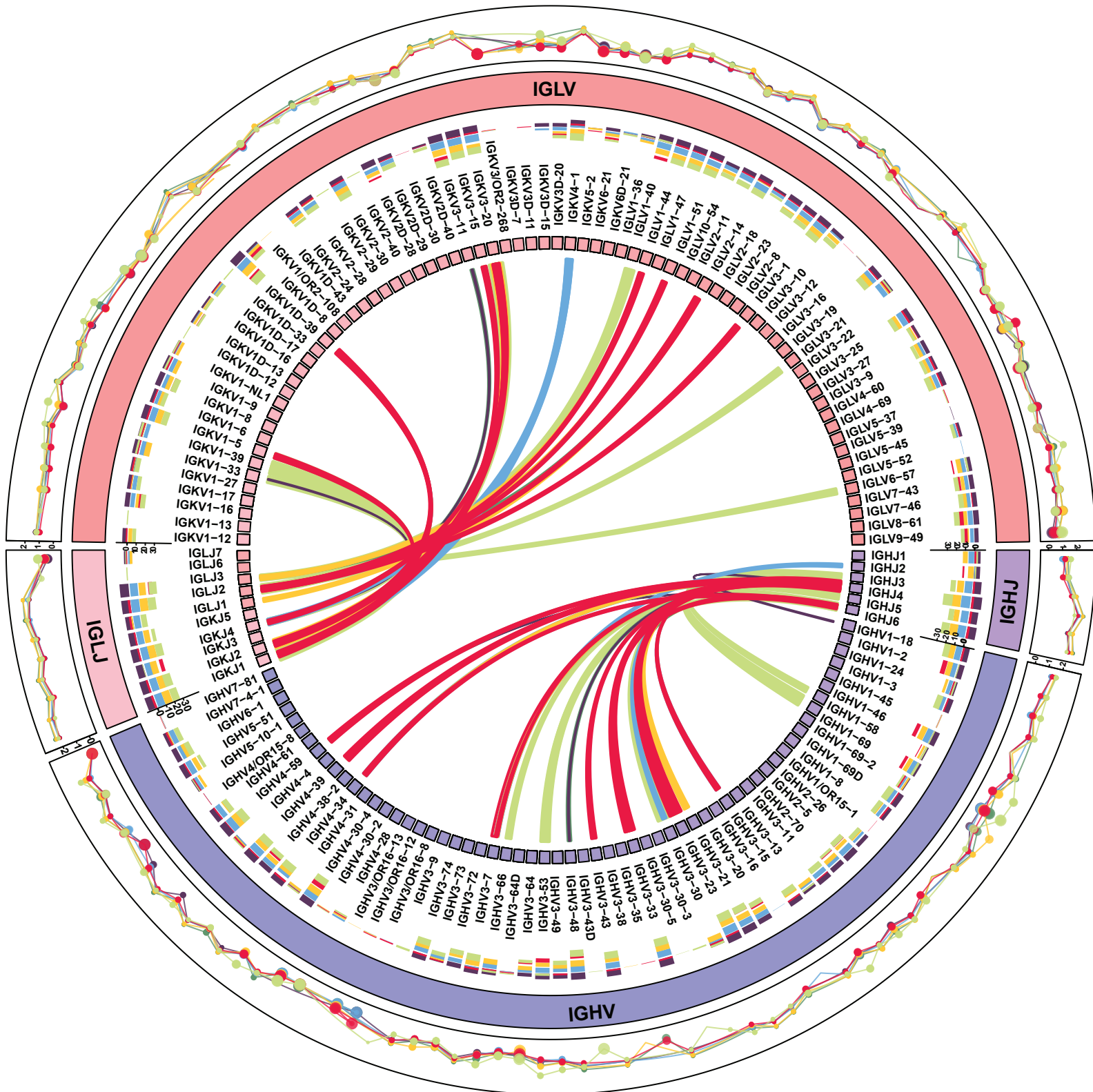

Group

- Published
- Mild short
- Mild long
- Severe short
- Severe long

 $\log_{10}(\text{sd mean})$ 

- 0.0
- 0.5
- 1.0
- 2.0

Pair index/ $10^2$ 

- 0.25
- 0.5
- 0.75
- 1.0

Supplement: Supplementary file 20 — Supporting Figure 20. BCR VJC gene usage frequency statistical analysis. [file JMV-97-e70335-s001.pdf]

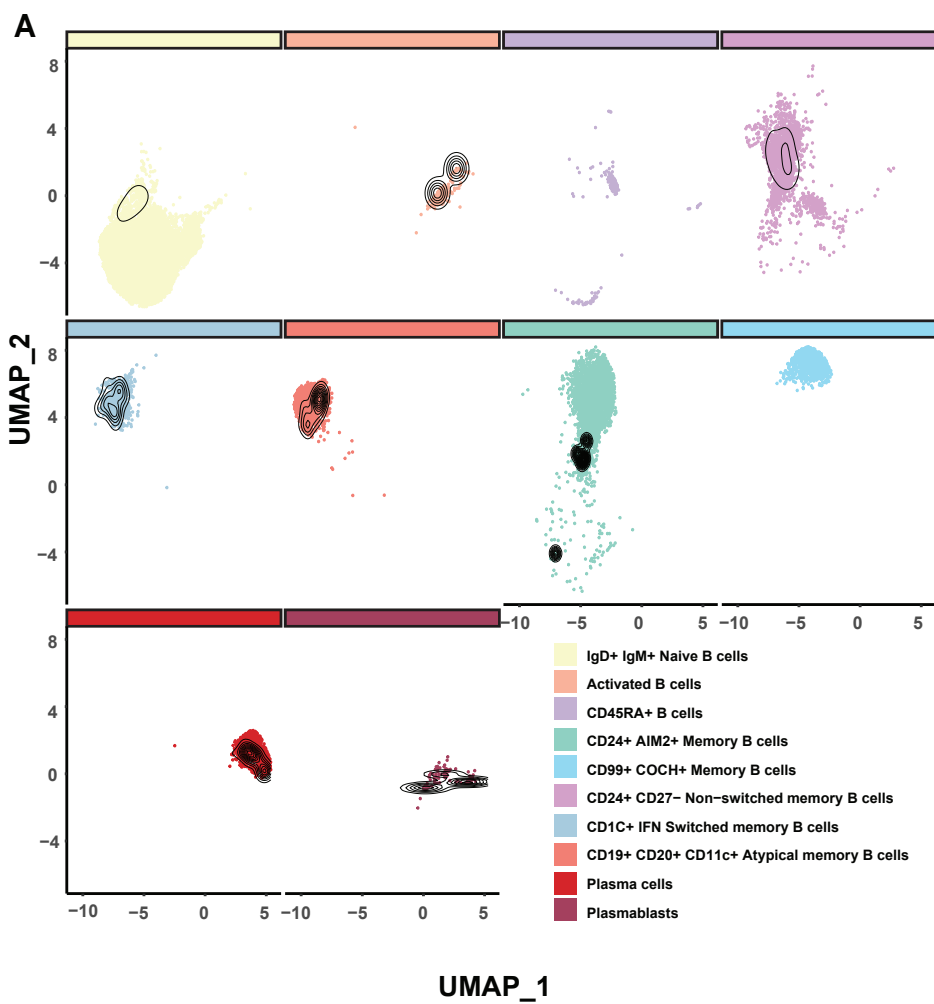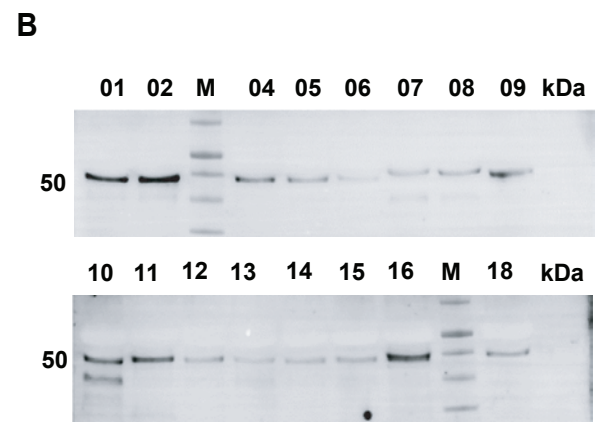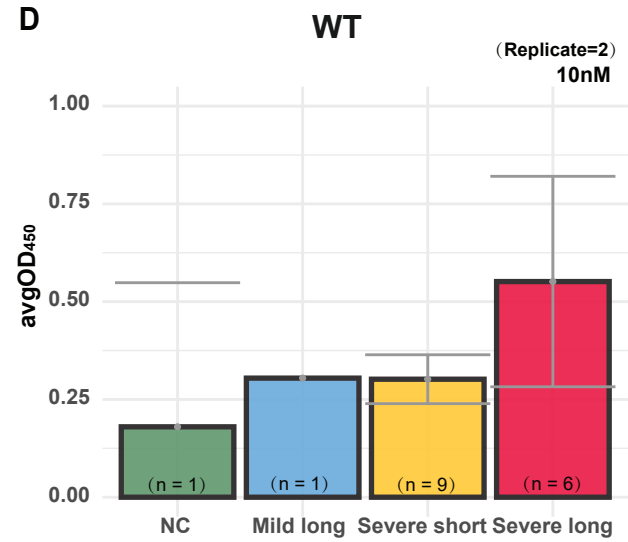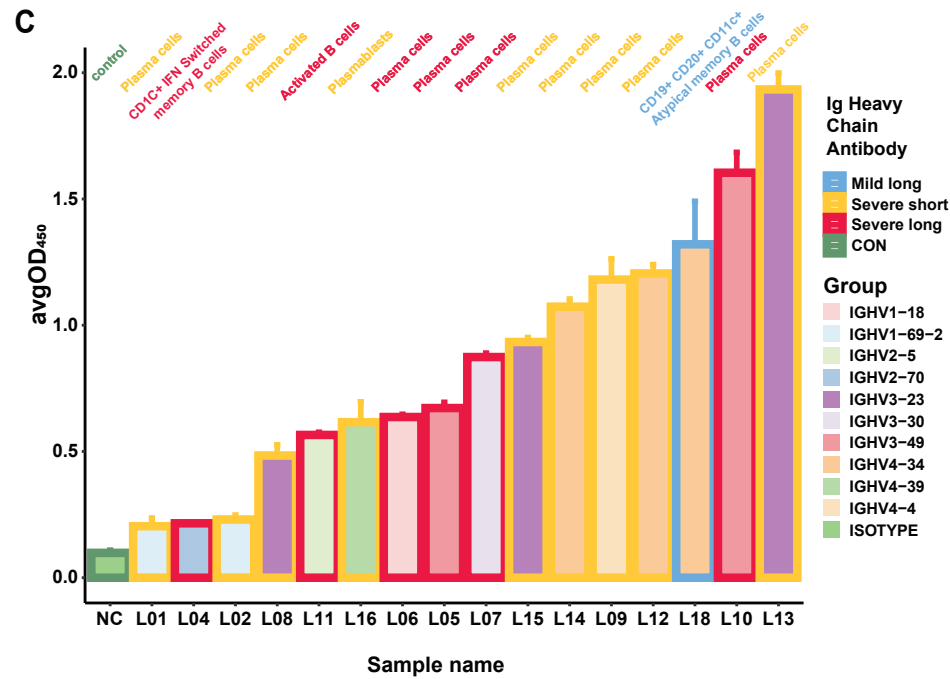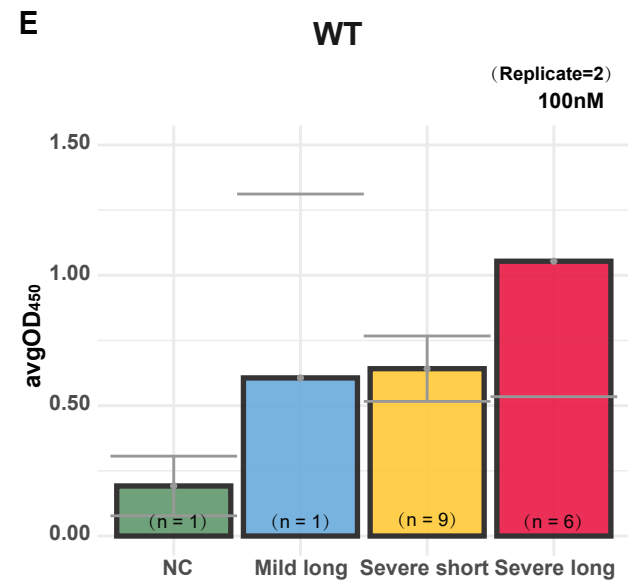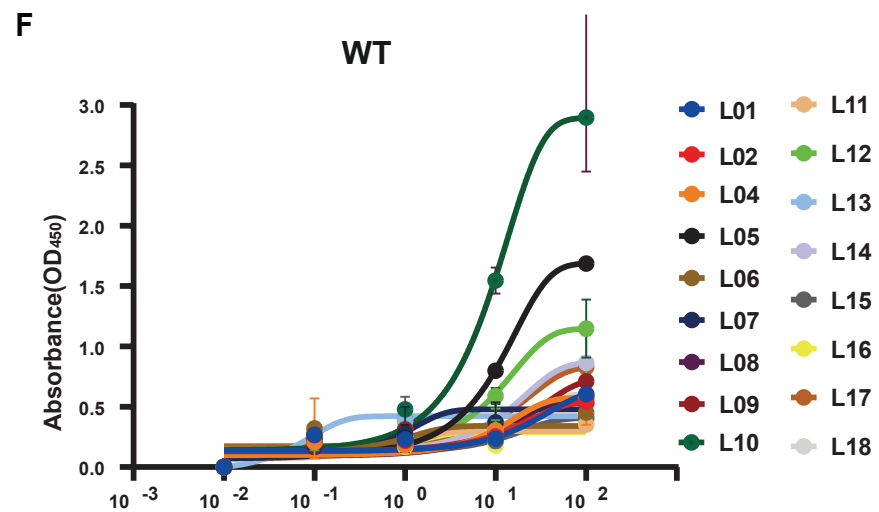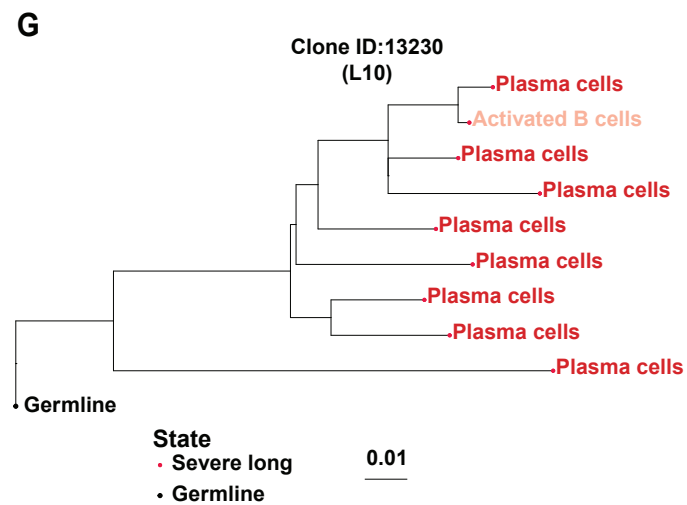

Supplement: Supplementary file 21 — Supporting Figure 21. In vitro determination of neutralizing antibody binding and antigen potency against the SARS‐CoV‐2 prototype strain. [file JMV-97-e70335-s009.pdf]

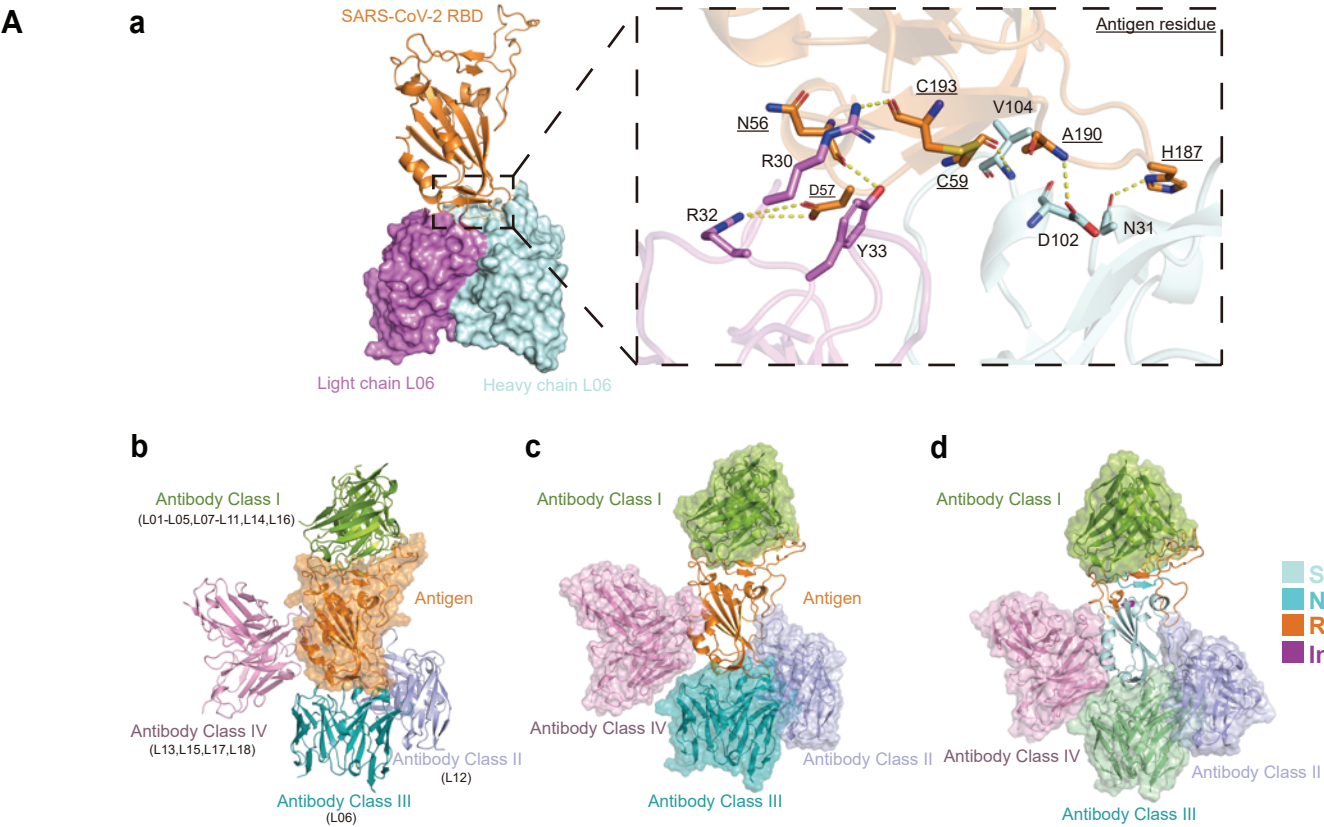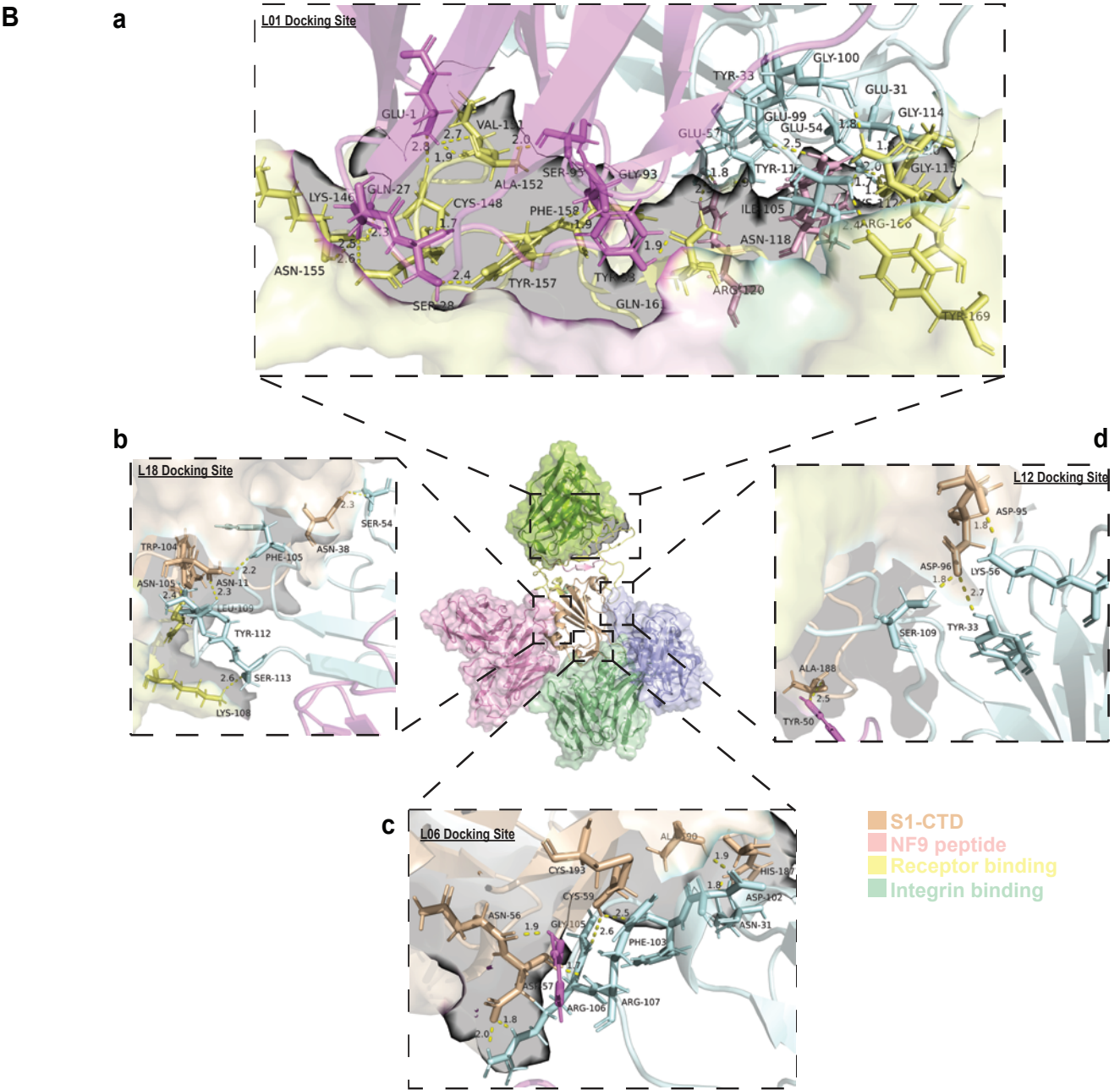

Supplement: Supplementary file 23 — Supporting Figure 23. Predicted commutative crystal structures of neutralizing antibodies with SARS‐CoV‐2 Omicron RBD using AlphaFold2. [file JMV-97-e70335-s023.pdf]
