# Supplementary material for: Myeloid‐Driven Immune Suppression Subverts Neutralizing Antibodies and T Cell Immunity in Severe COVID‐19
Source: J Med Virol. 2025 Apr 4;97(4):e70335. doi: 10.1002/jmv.70335 (PMC11969634; doi:10.1002/jmv.70335)
Supplement: Supplementary file 9 — Supporting Figure 9. Differential cell interactions among myeloid cell subpopulations across different groups. [file JMV-97-e70335-s024.pdf]

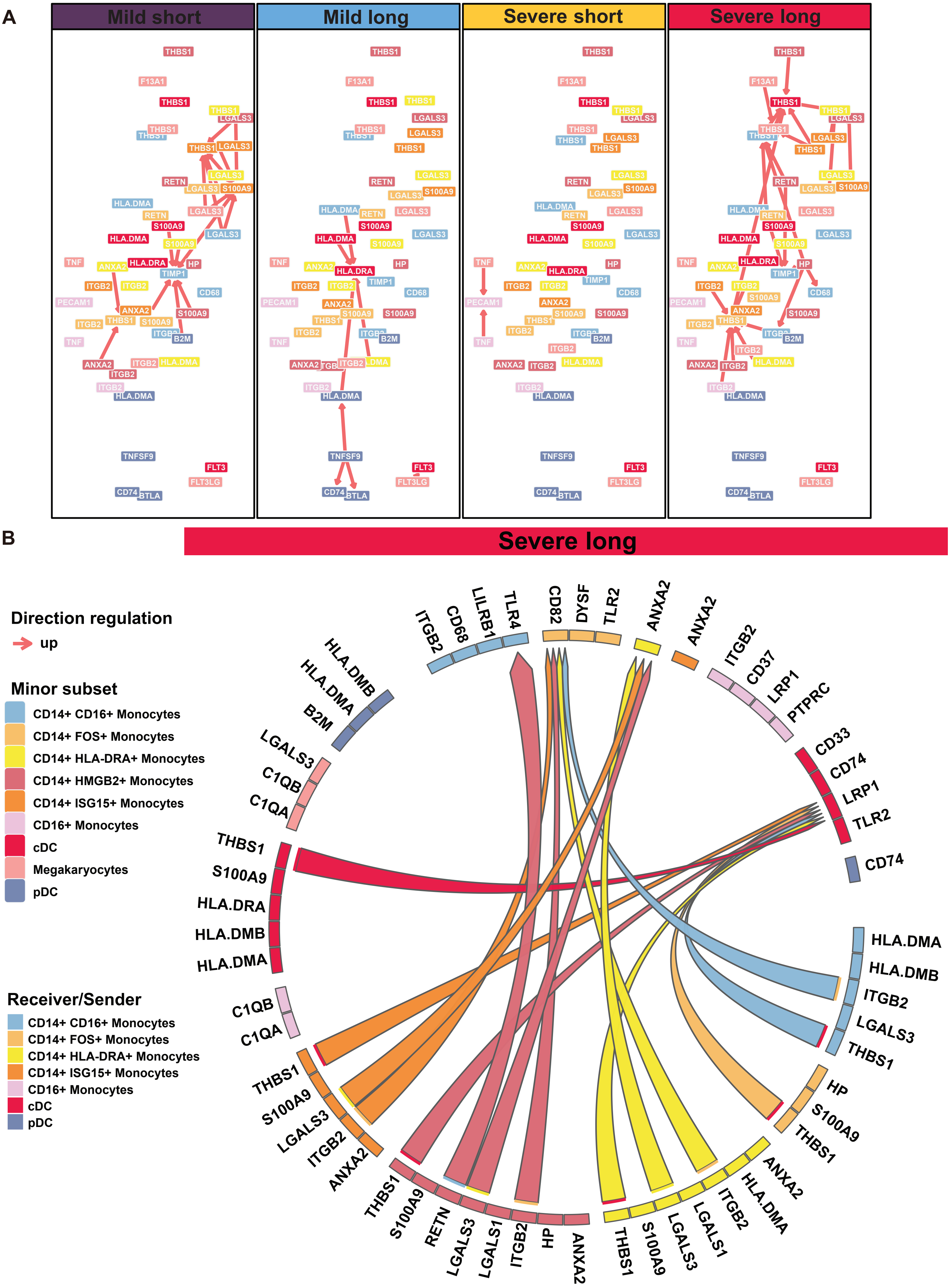
A
Mild short
Mild long
Severe short
Severe long
B
Severe long
Direction regulation
up
Minor subset
CD14+ CD16+ Monocytes
CD14+ FOS+ Monocytes
CD14+ HLA-DRA+ Monocytes
CD14+ HMGB2+ Monocytes
CD14+ ISG15+ Monocytes
CD16+ Monocytes
cDC
Megakaryocytes
pDC
Receiver/Sender
CD14+ CD16+ Monocytes
CD14+ FOS+ Monocytes
CD14+ HLA-DRA+ Monocytes
CD14+ ISG15+ Monocytes
CD16+ Monocytes
cDC
pDC
ITGB2
CD68
LILRB1
TLR4
CD82
DYSF
TLR2
ANXA2
ANXA2
ITGB2
CD37
LRP1
PTPRC
CD33
CD74
LRP1
TLR2
CD74
HLA.DMA
HLA.DMB
ITGB2
LGALS3
THBS1
HP
S100A9
THBS1
ANXA2
HLA.DMA
ITGB2
LGALS1
LGALS3
S100A9
THBS1
ANXA2
HP
ITGB2
LGALS1
LGALS3
RETN
S100A9
THBS1
ANXA2
ITGB2
LGALS3
S100A9
THBS1
C1QA
C1QB
HLA.DMA
HLA.DMB
HLA.DRA
S100A9
THBS1
C1QA
C1QB
LGALS3
B2M
HLA.DMA
HLA.DMB
