# Supplementary material for: Myeloid‐Driven Immune Suppression Subverts Neutralizing Antibodies and T Cell Immunity in Severe COVID‐19
Source: J Med Virol. 2025 Apr 4;97(4):e70335. doi: 10.1002/jmv.70335 (PMC11969634; doi:10.1002/jmv.70335)
Supplement: Supplementary file 17 — Supporting Figure 17. Differential gene set scoring and analysis of B cells subpopulations across disease severities. [file JMV-97-e70335-s017.pdf]

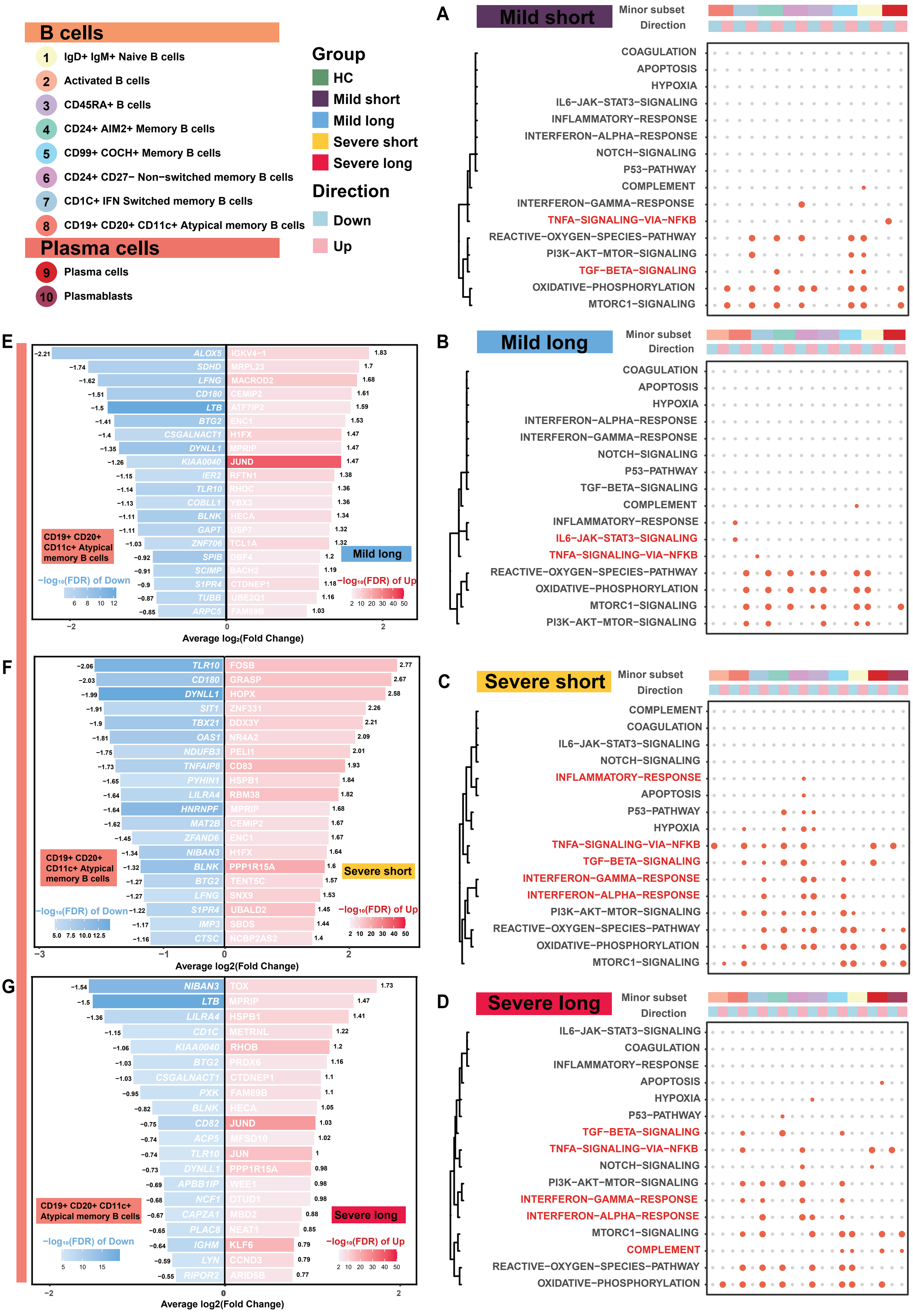

B cells
1 IgD+ IgM+ Naive B cells
2 Activated B cells
3 CD45RA+ B cells
4 CD24+ AIM2+ Memory B cells
5 CD99+ COCH+ Memory B cells
6 CD24+ CD27− Non−switched memory B cells
7 CD1C+ IFN Switched memory B cells
8 CD19+ CD20+ CD11c+ Atypical memory B cells
Plasma cells
9 Plasma cells
10 Plasmablasts
Group
HC
Mild short
Mild long
Severe short
Severe long
Direction
Down
Up
A
Mild short
Minor subset
Direction
COAGULATION
APOPTOSIS
HYPOXIA
IL6−JAK−STAT3−SIGNALING
INFLAMMATORY−RESPONSE
INTERFERON−ALPHA−RESPONSE
NOTCH−SIGNALING
P53−PATHWAY
COMPLEMENT
INTERFERON−GAMMA−RESPONSE
TNFA−SIGNALING−VIA−NFKB
REACTIVE−OXYGEN−SPECIES−PATHWAY
PI3K−AKT−MTOR−SIGNALING
TGF−BETA−SIGNALING
OXIDATIVE−PHOSPHORYLATION
MTORC1−SIGNALING
B
Mild long
Minor subset
Direction
COAGULATION
APOPTOSIS
HYPOXIA
INTERFERON−ALPHA−RESPONSE
INTERFERON−GAMMA−RESPONSE
NOTCH−SIGNALING
P53−PATHWAY
TGF−BETA−SIGNALING
COMPLEMENT
INFLAMMATORY−RESPONSE
IL6−JAK−STAT3−SIGNALING
TNFA−SIGNALING−VIA−NFKB
REACTIVE−OXYGEN−SPECIES−PATHWAY
OXIDATIVE−PHOSPHORYLATION
MTORC1−SIGNALING
PI3K−AKT−MTOR−SIGNALING
C
Severe short
Minor subset
Direction
COMPLEMENT
COAGULATION
IL6−JAK−STAT3−SIGNALING
NOTCH−SIGNALING
INFLAMMATORY−RESPONSE
APOPTOSIS
P53−PATHWAY
HYPOXIA
TNFA−SIGNALING−VIA−NFKB
TGF−BETA−SIGNALING
INTERFERON−GAMMA−RESPONSE
INTERFERON−ALPHA−RESPONSE
PI3K−AKT−MTOR−SIGNALING
REACTIVE−OXYGEN−SPECIES−PATHWAY
OXIDATIVE−PHOSPHORYLATION
MTORC1−SIGNALING
D
Severe long
Minor subset
Direction
IL6−JAK−STAT3−SIGNALING
COAGULATION
INFLAMMATORY−RESPONSE
APOPTOSIS
HYPOXIA
P53−PATHWAY
TGF−BETA−SIGNALING
TNFA−SIGNALING−VIA−NFKB
NOTCH−SIGNALING
PI3K−AKT−MTOR−SIGNALING
INTERFERON−GAMMA−RESPONSE
INTERFERON−ALPHA−RESPONSE
MTORC1−SIGNALING
COMPLEMENT
REACTIVE−OXYGEN−SPECIES−PATHWAY
OXIDATIVE−PHOSPHORYLATION
E
−2.21 ALOX5
−1.74 SDHD
−1.62 LFNG
−1.51 CD180
−1.5 LTB
−1.41 BTG2
−1.4 CSGALNACT1
−1.35 DYNLL1
−1.26 KIAA0040
−1.15 IER2
−1.14 TLR10
−1.13 COBLL1
−1.11 BLNK
−1.11 GAPT
−1.03 ZNF706
−0.92 SPIB
−0.91 SCIMP
−0.9 S1PR4
−0.87 TUBB
−0.85 ARPC5
IGKV4−1 1.83
MRPL23 1.7
MACROD2 1.68
CEMIP2 1.61
ATF7IP2 1.59
ENC1 1.53
H1FX 1.47
MPRIP 1.47
JUND 1.47
RFTN1 1.38
RHOC 1.36
YBX3 1.36
HECA 1.34
USP7 1.32
TCL1A 1.32
DBF4 1.2
BACH2 1.19
CTDNEP1 1.18
UBE2Q1 1.16
FAM89B 1.03
CD19+ CD20+ CD11c+ Atypical memory B cells
Mild long
−log10(FDR) of Down
6 8 10 12
−log10(FDR) of Up
2 10 20 30 40 50
−2 0 2
Average log2(Fold Change)
F
−2.06 TLR10
−2.03 CD180
−1.99 DYNLL1
−1.91 SIT1
−1.9 TBX21
−1.81 OAS1
−1.75 NDUFB3
−1.73 TNFAIP8
−1.65 PYHIN1
−1.64 LILRA4
−1.64 HNRNPF
−1.62 MAT2B
−1.45 ZFAND6
−1.34 NIBAN3
−1.32 BLNK
−1.27 BTG2
−1.27 LFNG
−1.22 S1PR4
−1.17 IMP3
−1.16 CTSC
FOSB 2.77
GRASP 2.67
HOPX 2.58
ZNF331 2.26
DDX3Y 2.21
NR4A2 2.09
PELI1 2.01
CD83 1.93
HSPB1 1.84
RBM38 1.82
MPRIP 1.68
CEMIP2 1.67
ENC1 1.67
H1FX 1.64
PPP1R15A 1.6
TENT5C 1.57
SNX9 1.53
UBALD2 1.45
SBDS 1.44
NCBP2AS2 1.4
CD19+ CD20+ CD11c+ Atypical memory B cells
Severe short
−log10(FDR) of Down
5.0 7.5 10.0 12.5
−log10(FDR) of Up
2 10 20 30 40 50
−3 −1 0 2
Average log2(Fold Change)
G
−1.54 NIBAN3
−1.5 LTB
−1.36 LILRA4
−1.15 CD1C
−1.06 KIAA0040
−1.03 BTG2
−1.03 CSGALNACT1
−0.95 PXK
−0.82 BLNK
−0.75 CD82
−0.74 ACP5
−0.74 TLR10
−0.73 DYNLL1
−0.69 APBB1IP
−0.68 NCF1
−0.67 CAPZA1
−0.65 PLAC8
−0.64 IGHM
−0.59 LYN
−0.55 RIPOR2
TOX 1.73
MPRIP 1.47
HSPB1 1.41
METRNL 1.22
RHOB 1.2
PRDX6 1.16
CTDNEP1 1.1
FAM89B 1.1
HECA 1.05
JUND 1.03
MFSD10 1.02
JUN 1
PPP1R15A 0.98
WEE1 0.98
OTUD1 0.98
MBD2 0.88
NEAT1 0.85
KLF6 0.79
CCND3 0.79
ARID5B 0.77
CD19+ CD20+ CD11c+ Atypical memory B cells
Severe long
−log10(FDR) of Down
5 10 15
−log10(FDR) of Up
2 10 20 30 40 50
−2 0 2
Average log2(Fold Change)
