# Supplementary material for: Myeloid‐Driven Immune Suppression Subverts Neutralizing Antibodies and T Cell Immunity in Severe COVID‐19
Source: J Med Virol. 2025 Apr 4;97(4):e70335. doi: 10.1002/jmv.70335 (PMC11969634; doi:10.1002/jmv.70335)
Supplement: Supplementary file 22 — Supporting Figure 22. Analysis of BCR heavy chain amino acid CDR3 sequence similarity and clonal germline evolution. [file JMV-97-e70335-s016.pdf]

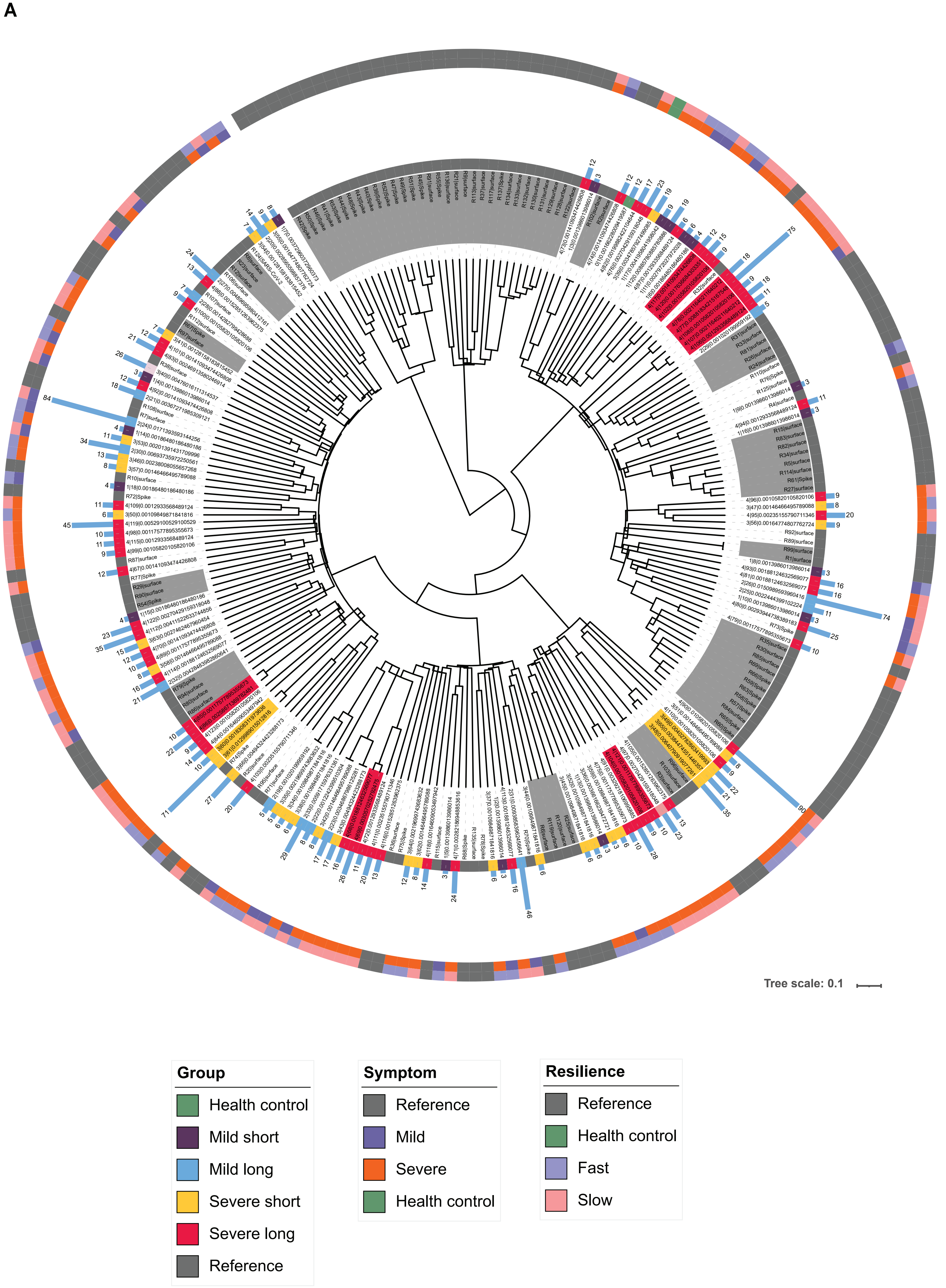
A
Tree scale: 0.1
Group
Health control
Mild short
Mild long
Severe short
Severe long
Reference
Symptom
Reference
Mild
Severe
Health control
Resilience
Reference
Health control
Fast
Slow
