# Supplementary material for: Myeloid‐Driven Immune Suppression Subverts Neutralizing Antibodies and T Cell Immunity in Severe COVID‐19
Source: J Med Virol. 2025 Apr 4;97(4):e70335. doi: 10.1002/jmv.70335 (PMC11969634; doi:10.1002/jmv.70335)
Supplement: Supplementary file 24 — Supporting Figure Captions 250210 revised. [file JMV-97-e70335-s028.docx]

**Supplementary Figure Captions**

**Supplementary Figure 1.** Quality control, batch effect correction, cell annotation, and major cell type proportions.

(A) Violin plot showing the distribution of the number of detected genes per sample. (B) Violin plot of gene counts per sample. (C) Distribution of mitochondrial gene content per sample. (D) Distribution of erythrocyte content per sample. (E) Batch effect correction visualization based on Harmony. The integration across groups shows consistent batch correction results. (F) Hierarchical circular plot of cell annotations, with circle size representing the number of cells in each category. Different coloured layers indicate annotation levels, including Cell type, Major subset, and Minor subset.

**Supplementary Figure 2.** Feature plots of canonical gene markers.

(A) UMAP Distribution of 21 Gene Expressions. *CST3*, *LYZ*, *CD14*, *FCGR3A*, *CD1C*, and *CLEC4C* are primarily markers for myeloid cells and dendritic cells. *GNLY*, *NKG7*, *CD3E*, *CD8A*, *CD40LG*, *CD4*, *IL7R*, *CCR7*, and *MKI67* serve as distinguishing markers for T cells and proliferative cells. *MS4A1*, *CD79A*, *IGHM*, *IGHG1*, *MZB1*, and *CD38* are predominantly markers for B cells and plasma cells. The color represents the level of gene expression.

**Supplementary Figure 3.** Dot plot of gene markers for NK cells and T lymphocytes.

(A) Dot size corresponds to the proportion of cells expressing a given gene within the cell subset, while color intensity reflects the average expression level of the gene. Distinct colors represent different cell types.

**Supplementary Figure 4.** Visualization of cell annotation marker genes.

(A) Dot plot of gene markers for myeloid cells. The color intensity of the dots represents expression levels, while the dot size indicates the proportion of cells within the cluster expressing the gene. Different colors correspond to different cell types. (B) Dot plot of gene markers for B cells and plasma cells. (C) Dot plot of gene markers for *CD4*+ T cells.

**Supplementary Figure 5.** Analysis of Cell Subpopulation Proportions under Different Clinical Indications.

(A) Box plots showing the proportion of proliferative cells across age groups. Different colours represent different groups, and the colored banners correspond to the cell types in Figure 1C. Different shapes indicate clinical statuses: circles represent unvaccinated survivors, squares represent vaccinated survivors, upward triangles represent unvaccinated deceased, and downward triangles represent vaccinated deceased. Statistical significance was determined using Student’s *t*-test (*p < 0.05, **p < 0.01, ***p < 0.001). (B-C) Box plots showing the proportion of *CD4*+ naïve T cells and iNKT cells between healthy and patient groups (HVSC). (D-E) Box plots comparing the proportion of *CD14+ FOS+* monocytes *and CD14+ HMGB2+* monocytes between healthy and patient groups. (F-G) Box plots showing the proportion of *CD14*+ *FOS*+ monocytes and *CD14*+ *HMGB2*+ monocytes in groups classified by recovery speed. (H) Box plots showing the proportion of *CD4*+ Th1 cells under clinical outcomes of survival or death. (I) Box plots showing the proportion of *CD4+* Th1 cells with and without vasoactive drug treatment. (J) Box plots showing the proportion of MAIT cells under clinical outcomes of survival or death. (K) Box plots showing the proportion of MAIT cells with and without vasoactive drug treatment. (L) Box plots showing the proportion of Treg cells with or without Paxlovid treatment. (M) Box plots showing the proportion of *CD4*+ *GZMK*+ Th1 cells across different symptom groups. (N) Box plots showing the proportion of *CD4*+ Th2 cells in groups classified by recovery speed. (O) Box plots showing the proportion of CD19+ CD20+ CD11c+ atypical memory B cells with or without steroid treatment. (P-Q) Box plots showing the proportion of plasma cells and pDCs across different symptom groups.

**Supplementary Figure 6.** Immune cell infiltration analysis and correlation analysis of ISGs gene set scores.

(A) Heatmap of immune infiltration correlation analysis, calculated using Spearman correlation. Colors indicate the strength of the correlation, and pie charts represent the proportion of related cells. (B) Heatmap of ISGs gene set score correlations. Fold change represents the average expression difference in each subpopulation between case groups and the healthy control group. Correlation analysis was performed using Pearson correlation.

**Supplementary Figure 7.** Relative cell proportions of myeloid cells under varying disease severities.

(A) Distribution map of expression levels for characteristic genes including *PLBD1*, *VCAN*, *S100A8/9/12* and *HLA-DRA* related to MNSCs, scaled. (B) Proportional statistics chart of *CD14*+ *ISG15*+ monocytes under varying symptoms. (C) Proportional statistics chart of *CD14*+ *FOS*+ monocytes under varying symptoms, analyzed using Student's *t*-test (*p < 0.05, **p < 0.01, ***p < 0.001). (D-E) Proportional statistics chart comparing *CD14*+ *HMGB2*+ monocytes and *CD14*+ *HLA-DRA*+ monocytes under varying symptoms. (F-G) Proportional statistics chart for CD16+ monocytes and *CD14*+ CD16+ monocytes under varying symptoms. (H-J) Proportional statistics chart for megakaryocytes, pDCs, and cDCs under varying symptoms.

**Supplementary Figure 8.** Violin plots of MDSC-like marker expression in *CD14*+ *HMGB2*+ monocytes across symptom severity groups.

(A–G) Expression distribution of selected genes in *CD14*+ *HMGB2*+ monocytes stratified by symptom severity. (A) *PLAC8*, (B) *S100A8*, (C) *S100A9*, (D) *IL1R2*, (E) *CD163*, (F) *HLA-DRA*, and (G) *MAFB*. Pairwise comparisons between groups were performed using Student’s *t*-test (*p < 0.05, **p < 0.01, ***p < 0.001).

**Supplementary Figure 9.** Differential cell interactions among myeloid cell subpopulations across different groups.

(A) Network diagram of the top 25 ligand-receptor pairs based on MultiNicheNet prioritization, with arrow directions indicating upregulated receptors. (B) Distribution map of the top 50 total ligand-receptor pairs, with different colors representing different cell types in severe long group. Arrows originate from ligands and point towards receptors.

**Supplementary Figure 10.** Differential ligand-receptor interactions across various clinical conditions.

(A) Visualization of the expression and activity of these interactions for the severe long group. Rows represent specific ligand-receptor pairs, with heatmaps showing ligand activity (grey-pink) and dot colours/sizes indicating expression levels, cell type specificity. Black dots indicate sufficient presence of sender and receiver cells. (B-C) Boxplots of cytokine concentration levels across different symptom groups in plasma. Displaying differences in the levels of cytokines IL-17A and I-TAC across different symptom groups. Statistical significance was assessed using Student's *t*-test. Concentrations are log10 normalized and expressed in ng/ml.

**Supplementary Figure 11.** UMAP visualization of T lymphocytes, NK cells, and TCR clonotype distributions.

(A) UMAP plot displaying the distribution of T lymphocytes and NK cells. (B-F) Distribution of the top 5 most abundant clonotypes in different groups: Severe short (B), Severe long (C), Healthy control (D), Mild short (E), and Mild long (F). (G) Diversity statistics of TCR across different groups, based on individual assessments. The analysis was performed using 100 iterations to assess diversity comprehensively.

**Supplementary Figure 12.** Clone expanded distribution and gene expression dot plots of T lymphocytes and NK cells.

(A) Clonotype distribution of T lymphocytes visualized on UMAP, with colors indicating the degree of clonal expansion: gold for hyperexpanded (100 < X ≤ 500), orange for large (20 < X ≤ 100), red-orange for medium (5 < X ≤ 20), maroon for small (0 < X ≤ 5), and gray for NA. (B) Dot plots showing gene expression of TNF and IFNγ across minor subsets of T lymphocytes and NK cells. Dot size represents the proportion of cells expressing the gene within the subset, and color intensity reflects the average expression level. (C) Dot plots of TNF and IFNγ expression in CD8+ terminal effector T cells across symptom severity groups.

**Supplementary Figure 13.** Immunogenomic analysis and gene set scoring significance for T lymphocytes and NK cells.

(A) Heatmap displaying the results of immune-related gene set scores for all T lymphocytes and NK cells. Statistical significance across different cell types within these gene sets was assessed using the Kruskal-Wallis test, with p-values adjusted to q-values. (*p < 0.05, **p < 0.01, ***p < 0.001). (B) Volcano plot of differential expression in CD8+ Effector memory T cells across various disease groups relative to healthy controls. The plot highlights the top and bottom 10 genes by mean expression. The y-axis represents the log2 normalized expression difference (Fold change), and the x-axis shows the adjusted p-values. (C) Venn diagram showing differential gene expression in CD8+ effector memory T cells across different disease groups compared to healthy controls.

**Supplementary Figure 14.** Pathway enrichment visualization of differential genes in CD8+ Effector Memory T cells across different disease groups.

(A) Bubble chart displaying pathway enrichment for upregulated and downregulated genes in CD8+ effector Memory T cells. Only pathways potentially relevant to immune involvement are shown. The size of each bubble represents the proportion of genes enriching each pathway, while the color intensity indicates the significance level of the p-values.

**Supplementary Figure 15.** Differential analysis of *CD4*+ *GZMK*+ Th1 cells across various groups.

(A) Volcano plot illustrating differential gene expression in *CD4*+ *GZMK*+ Th1 cells relative to healthy controls. Only the top 10 upregulated and top 10 downregulated genes in each group are labeled with gene symbols. The x-axis shows the adjusted p-values, while the y-axis represents the log2 normalized fold changes. (B) Differences in gene set scores related to TNF signaling via NFkB in *CD4*+ *GZMK*+ Th1 cells across different groups. Statistical significance was assessed using Student's *t*-test (*p < 0.05, **p < 0.01, ***p < 0.001). (C) Bubble chart displaying pathway enrichment for upregulated and downregulated genes in *CD4*+ *GZMK*+ Th1 cells. Only pathways potentially relevant to immune involvement are shown. The size of each bubble represents the proportion of genes enriching each pathway, while the color intensity indicates the significance level of the p-values.

**Supplementary Figure 16.** Composition Analysis and Pathway Enrichment Scores of B Cells.

(A-H) Box plots illustrating the proportions of various B cell subpopulations across different groups: IgD+ IgM+ Naïve B cells, Activated B cells, CD45RA+ B cells, Plasmablasts, CD24+ AIM2+ memory B cells, CD99+ COCH+ memory B cells, CD24+ CD27- Non-switched memory B cells, and CD1C+ IFN Switched memory B cells. Green represents healthy controls; purple, mild short; blue, mild long; yellow, severe short; and red, severe long. Different shapes indicate clinical statuses: circles for unvaccinated survivors, squares for vaccinated survivors, upward triangles for unvaccinated deceased, and downward triangles for vaccinated deceased. Statistical significance was assessed using Student's *t*-test (*p < 0.05, **p < 0.01, ***p < 0.001). (I) UMAP plot showing the AUC scores for the TNFa signaling via NFkB pathway across different disease groups, with color intensity indicating the level of pathway enrichment.

**Supplementary Figure 17.** Differential Gene Set Scoring and Analysis of B Cells Subpopulations Across Disease Severities.

(A-D) Bubble charts of gene set scores for different cell types within various case groups. (A) represents the mild short group, (B) the mild long group, (C) the severe short group, and (D) the severe long group. (E-G) Bar charts visualizing the top 10 upregulated and downregulated genes in CD19+ CD20+ CD11c+ Atypical memory B cells relative to healthy controls across different disease groups. Chart (E) corresponds to the mild long group, (F) to the severe short group, and (G) to the severe long group. Darker shades indicate higher -log10(FDR) values, with red denoting upregulated genes in more severe cases, and blue indicating downregulation. Due to a lack of significant differential genes in the mild short group compared to healthy controls, this group has been omitted from visualization.

**Supplementary Figure 18.** Differential Gene Analysis of Plasma Cells and CD19+ CD20+ CD11c+ Atypical Memory B Cells.

(A) Venn diagram illustrating the differential genes between plasma cells in different case groups and the healthy control group. (B) Venn diagram showing the differential genes between CD19+ CD20+ CD11c+ Atypical memory B cells in different case groups and the healthy control group. (C) Bubble chart for the functional pathway enrichment analysis of differential genes in plasma cells. The size of each bubble represents the proportion of called genes within that pathway relative to the total genes in the pathway. The color intensity indicates the significance of the p-values.

**Supplementary Figure 19.** BCR Clonal Status Analysis and Clonotype Diversity Assessment.

(A) Bar chart representing the statistics of unique clonotypes for different patients across various groups. Colors denote different groups: green for healthy controls; purple for mild short; blue for mild long; yellow for severe short; and red for severe long. (B) Diversity statistics of BCR across different groups, based on individual assessments. The analysis was conducted using 100 iterations to ensure a thorough assessment of diversity. (C) Diversity assessment of clonotypes across different groups, using diversity indices ranging from q=0 to q=5 with a step increment of 0.1 and a 95% confidence interval to measure diversity comprehensively. (D) Box plots showing the *IGHG1* gene usage frequency of BCR heavy chain constant region genes IGHC across different groups. Statistical significance was assessed using Student's *t*-test (*p < 0.05, **p < 0.01, ***p < 0.001). Different shapes indicate clinical statuses: circles for unvaccinated survivors, squares for vaccinated survivors, upward triangles for unvaccinated deceased, and downward triangles for vaccinated deceased.

**Supplementary Figure 20.** BCR VJC Gene Usage Frequency Statistical Analysis.

(A) Circular visualization of BCR light and heavy chain V-J gene usage frequencies and pairing numbers. Different colors represent different groups. The outermost ring shows the standard deviation and mean of specific genes in groups, with point size representing the inverse of the standard deviation of the log10 normalized mean values—larger points indicate smaller standard deviations. The Y-axis of this ring represents the log10 normalized mean values. The middle ring displays bar charts of gene usage frequency differences, calculated by subtracting the gene usage frequency of the healthy group from other disease and public dataset groups, with the Y-axis showing the log10 normalized frequencies. The inner circle illustrates the top 20 V-J gene pairing situations in each group with lines, where the pairing score is calculated using entropy weighting of the pairing number and its rank within all BCRs, normalized for preference strength. Line width indicates the magnitude of the pairing scores. Color fill varies by group: light green for the published dataset, purple for mild short, blue for mild long, yellow for severe short, and red for severe long.

**Supplementary Figure 21.** In Vitro Determination of Neutralizing Antibody Binding and Antigen Potency against the SARS-CoV-2 Prototype Strain.

(A) Overlay map of BCR distribution across different B cell subpopulations. Similar to a topographic map, denser circles indicate a higher degree of clonal expansion. Different colors represent different B cell subpopulations. (B) Western blot results showing binding of in vitro expressed antibodies from HEK293 cell lines to the SARS-CoV-2 prototype strain. (C) Bar chart of optical density measuring the binding intensity of in vitro expressed antibodies from HEK293 cell lines to the SARS-CoV-2 prototype strain. Different fill colors represent antibodies' heavy chain V genes, while different border colors indicate different groups. (D) Grouped bar chart showing the absorbance from in vitro antibody binding wild type ELISA at 10nM prototype strain antigen concentration. The y-axis represents the average optical density from duplicate technical replicates. (E) Grouped bar chart showing the absorbance from in vitro antibody binding wild type ELISA at 100nM prototype strain antigen concentration. The y-axis represents the average optical density from duplicate technical replicates. (F) Fitting curve plot for antibody-antigen interaction across different concentration gradients of the prototype strain antigen. Each point denotes the average optical density from duplicate technical replicates, with different colors representing different antibodies. (G) Evolutionary analysis of clone germlines for L10 based on the heavy chain genes. The length of the lines represents the evolutionary distance from the germline. Different colours represent different cells, and each endpoint dot represents a BCR.

**Supplementary Figure 22.** Analysis of BCR Heavy Chain Amino Acid CDR3 Sequence Similarity and Clonal Germline Evolution.

(A) Similarity analysis of BCR heavy chain amino acid CDR3 regions. Different colors represent different groups. The tree scale indicates the specific distance. 'R' denotes the reference sequence, with the target protein specified after "|". The numbers after disease groups are arbitrary and carry no further significance.

**Supplementary Figure 23.** Predicted Commutative Crystal Structures of Neutralizing Antibodies with SARS-CoV-2 Omicron RBD Using AlphaFold2.

(A) Illustrative diagrams of the complex structures between different antibodies and the SARS-CoV-2 RBD. (a) Displays the binding mode and hydrogen bond locations for the L06 antibody with the RBD. (b) Shows the antigen RBD structure with surface mapping. (c) Presents the structures of various antibodies with surface mapping. (d) Details the domain distribution within the antigen RBD when interfaced with surface structures. Light aqua represents the S1 protein C-terminal domain (S1-CTD). Indigo denotes the NF9 peptide, an immunodominant HLA epitope recognized by CD8+ T cells, consisting of a 9 amino acid sequence, NYNYLYRLF. Orange highlights the receptor-binding domain, and purple marks the integrin binding sequence locations. (B) Molecular docking hydrogen bond diagrams for four different binding modes of antibodies. Brown represents the S1 CTD domain, yellow indicates the receptor-binding domain, light green denotes the integrin binding sites, pink highlights the NF9 peptide, magenta represents the heavy chain, and light aqua signifies the light chain. (a) Predicted docking site structure for the L01 antibody. (b) Predicted docking site structure for the L18 antibody. (c) Predicted docking site structure for the L06 antibody. (d) Predicted docking site structure for the L12 antibody.
