# Supplementary material for: Myeloid‐Driven Immune Suppression Subverts Neutralizing Antibodies and T Cell Immunity in Severe COVID‐19
Source: J Med Virol. 2025 Apr 4;97(4):e70335. doi: 10.1002/jmv.70335 (PMC11969634; doi:10.1002/jmv.70335)
Supplement: Supplementary file 25 — Supporting Materials & Methods Revised. [file JMV-97-e70335-s012.docx]

**Supplementary materials and methods**

## Study design and participants

This study involved 34 COVID-19 patients and 6 healthy donors from Zhongshan Hospital, Fudan University. All participants were Shanghai residents with an average age of 79 years and had laboratory-confirmed SARS-CoV-2 infections by RT-PCR between March and June 2022. Virus clearance was determined by two consecutive negative pharyngeal swab tests taken over 24 hours apart, with patients showing positive RT-PCR results for more than 10 days classified as having prolonged RNA shedding. The diagnosis and clinical severity were classified according to the ninth version of the Chinese Clinical Guidance for COVID-19 Pneumonia Diagnosis and Treatment, categorizing cases as mild, moderate, severe, or critical^1^.Patients were group as following: (1) Mild short: patients with asymptomatic, mild, moderate diagnosis and viral shedding time ≤ 10 days; (2) Mild long: patients with asymptomatic, mild, moderate diagnosis and viral shedding time > 10 days; (3) Severe short: patients with severe and critical diagnosis and viral shedding time ≤ 10 days; (4) Severe long: patients with severe and critical diagnosis and viral shedding time > 10 days. The exclusion criteria were patients (1) with tumours, (2) with rheumatic system diseases, (3) patients who have used immunosuppressants, (4) patients who have undergone stem cell or solid organ transplantation, (5) patients who have undergone biological therapy, (6) and patients who have been vaccinated within 1 year with vaccines other than the COVID-19 vaccine. All participants voluntarily donated blood samples. And all the patients voluntarily donated their blood samples.

The demographic and baseline information (age, gender, and residency district), vaccination, comorbidities, clinical manifestations, prognosis, laboratory examinations and treatments were available to study investigators. The study was approved by the Ethical Committee of Zhongshan Hospital, Fudan University, Shanghai, China (B2022-244R).

## Peripheral blood mononuclear cells (PBMCs) isolation and preparation for scRNA sequencing

Peripheral blood was collected in EDTA anticoagulated tubes. After diluted approximately 1:1 with sterile 1X phosphate-buffered saline (PBS, Thermo Fisher Scientific), blood was slowly centrifuged at 800 g for 25 minutes at room temperature after treating by Ficoll-Paque PLUS (Cytiva). The mononuclear cell layer was transferred and washed 3 times with PBS. After counting, cells were resuspended in CELLSAVING (NCM biotech) of 2×10^6^/ml, cryopreserved in Cryotubes (Corning) and then stored at -80℃. Samples were stored in liquid nitrogen. Cell viability and cell concentration were determined using a Countstar Automated Cell Counter. Cell viability of PBMCs was greater than 85% and the cell concentration was adjusted to 500–1200 cells/µL. PBMCs were loaded 16,000 cells/chip position using the 10× Chromium Next GEM Single Cell 5’ Kit v2. The following barcoding, cDNA synthesis and library construction were followed by standard manufacturer’s instructions. The qualified libraries were applied to the Illumina NovaSeq6000 platform for PE150 sequencing.

## Single-cell RNA-seq data processing

Single-cell transcriptome sequencing data were aligned to the GRCh38 human reference genome using Cell Ranger (v6.0.2) with Martian Runtime platform (v4.0.4). The data, after a basic cell count check, included an estimated median of 6,860 cells per sample, with a median genes number of approximately 1357 per cell. The dataset included cells with at least 500 counts and 200 detected genes, excluding those in the top 2.5% for both counts and gene numbers, while ensuring mitochondrial UMI counts were under 10% and erythrocyte proportions less than 1%. Quality control metrics for individual samples are detailed in **Supplementary Table 1**, with violin plots for each sample after QC were included in the **Supplementary Materials**.

## Unsupervised clustering analysis and batch effect correction

We integrated 40 objects using Seurat^2^, scaling and centering features with the NormalizeData function, and selected 30 dimensions via RunPCA. UMAP dimensionality reduction was applied to visualize cell distribution using the first 30 PCA dimensions. To correct batch effects, the Harmony algorithm was used, treating each sample as a variable. Clustering was performed using SNN modularity optimization with varying resolutions, and overall sample quality was consistent, except for a few samples (No.11, No.376, No.396) with low cell numbers or richness.

## Cell subset and annotations

With a view to potential the effect of cell cycles on annotation, we estimated the scores of cell cycles and lastly the evaluation indicated that no obvious cell cycle effect. Refer to COMBAT Consortium^3^, we classified six basic immune cell types including T cells, B cells, Myeloid cells, NK cells, Plasma cells and HSPC (Hematopoietic stem and progenitor cell). At the major subset level, T cells were subdivided into CD8+ naïve T cells, CD4+ naïve T cells, γδ-T cells (GDT), CD4+ cytotoxic T cells, CD4+ T cells, CD8+ T cells, regulatory T cells (Treg), and invariant NKT cells (iNKT). Similarly, NK cells were categorized into Natural Killer cells (NK) and cytotoxic NKT cells. Meanwhile, myeloid cells were divided into conventional DCs (cDC), plasmacytoid DCs (pDC), CD10+ Monocytes, CD16+ Monocytes, and megakaryocytes.

## Correlation analysis between clinical Information and cell proportions

Given that some clinical conditions involve multiple groups, we employed the non-parametric Kruskal-Wallis test to assess the significance of various cell types under different clinical statements, resulting in a significance heatmap. Additionally, box plots were used to visualize some of the significant results, where comparisons between groups were made using Student's *t*-test (**p* < 0.05, ***p* < 0.01, ****p* < 0.001, *****p* < 0.0001).

## Comparison for cell proportion

We calculated the proportion of different cell populations for each subject by dividing the number of cells in a given type by the total cell count. To assess differences in cell type proportions across patient phenotypes, a Student’s *t*-test was conducted between groups, with results visualized in a boxplot. The boxplot included different dot shapes to indicate subject state and vaccination status, along with significance levels marked by *p*-values (**p* < 0.05, ***p* < 0.01, ****p* < 0.001, *****p* < 0.0001).

## Immune repertoire V(D)J analysis

We used Cell Ranger (v.6.0.2) to assemble and quantify BCR/TCR sequences following the standard vdj protocol against the GRCh38 reference human genome. Assembled contigs were filtered out if they had low confidence, were non-full length, non-productive, or had a barcode not associated with a cell. TR or IG chains were identified based on criteria requiring at least 10,000 total reads, with mapped reads making up at least 5.0% of the total and 3.0x more than the other chain. Sample No.365 was removed from the BCR analysis because its mapped reads to IG did not meet the 5.0% threshold. Filtered contigs were imported into R, where we combined BCR/TCR data by removing chains without values and selecting the highest-expressing chains using the scRepertoire^4^. Additionally, alpha diversity curve assessments and germline inference were performed using the alakazam package^5^.

## Sequence comparison analysis

We evaluated clonotype frequency groups in Seurat objects based on BCR/TCR analysis, generating sequence documents for clonotypes with a frequency over 1. Complementarity Determining Region 3 (CDR3) of either BCR (heavy, light chains) or TCR (TCRα, TCRβ chains) were extracted from filtered contigs and categorized. Reference alignment sequences were downloaded from the IEDB database, focusing on SARS-CoV-2 sequences with positive assay results, while excluding those from T cell and MHC assays. Sequencing comparison analysis was performed with five guide-tree iterations and five HMM iterations, using Clustal Omega^6^.

## Identification of differential expressed genes and pathway enrichment analysis

Differential expressed genes were identified using the FindMarkers function for each cell type, comparing the case group to the healthy group, focusing only on positive markers with a minimum cell fraction of 0.15 in either population and an average log-fold difference greater than 0.25. Enriched Gene Ontology (GO) terms and Kyoto Encyclopedia of Genes and Genomes (KEGG) pathways were identified for different cell types with default parameters using the clusterProfiler R package^7^.

## Annotation correlation analysis

We selected the top 20 genes for each cell type based on p-value significance, fold change in expression levels, and expression proportions within and across cell types. Using this gene set, we performed immune cell infiltration analysis with the ssgsea method from the GSVA tool, setting 'mx.diff=FALSE' and retaining default parameters. Pearson's correlation coefficients were calculated on transposed matrix, followed by visualization using a pie chart heatmap.

## Production of monoclonal antibodies (mAb)

DNA products of paired heavy chains and light chains of individual BCR were synthesized and cloned to human IgG1 heavy-chain and IgK/L light-chain vectors to construct mAb heavy or light chain recombinant plasmids respectively. Equal amounts of IgG1 heavy-chain and IgK/L plasmids were transfected into CHO cells by using transfection reagent according to the manufacture’s procedure (Cat. A29133, Thermo Fisher). The culture medium was harvested by centrifugation. The supernatant containing secreted IgG antibodies were verified using western blot. Antibodies were further purified using a Protein A kit (GE Health Science) and subjected for ELISA assay.

## ELISA assay analysis

Enzyme-Linked ImmunoSorbent Assay (ELISA) was used to assess recombinant antibody-antigen interactions. The ELISA was performed as previously described^8^. In brief, Serail dilution of individual mAbs were added to S timer protein-coated and blocked 96-well microtiter plates at the required assay dilution. HRP conjugated goat anti-human IgG antibody, tetramethylbenzidine (TMB) substrate were added before measurement of optical density at 450 nm.

To compare results across each mAbs, a magnitude-breadth plot was provided to report absolute antibody concentrations across a panel of antigens. Magnitude-breadth curves assess the proportion of antibody concentrations (nM). Nonlinear fitting of five parameters (including EC50, HillSlope, S, Bottom, and Top) was performed using five different antigen concentrations (0.01, 0.1, 1, 10, 100 nM) in GraphPad Prism. The curve is characterized by Bottom and Top plateaus, with EC50 representing the half-maximal concentration, HillSlope indicating the slope factor, and S as the symmetry parameter.

## Structure docking prediction analysis

Eighteen antibody sequences were selected to ensure comprehensive coverage of B cell subpopulations for virus-antibody interaction modelling. Structural data for the SARS-CoV-2 Omicron BA.4/5 strain protein was obtained from the Protein Data Bank (PDB) with the accession code 7zxu. The protein structure was predicted using AlphaFold2^9^, with output in PDB format and default settings. The predicted structures were then visualized and processed using PyMOL software.

## Cytokine analysis

Following the completion of cytokine detection using the MSD instrument, the cytokine data were collected and their validity was ascertained. Subsequently, the data were organized into groups and imported into the R statistical platform. The ggplot2 package was utilized to visualize the data. Significance testing between groups was performed using Student's *t*-test.

## Cell interaction prediction analysis

Cell communication analysis and group comparisons across four disease groups were performed using MultiNicheNet^10^. Initially, each case group was compared to the healthy group to identify differential objects. These differentials were then used to compare long positive and rapidly recovering patients, highlighting differences related to virus clearance time, and to distinguish between mild and severe cases. During cell interaction object construction, we set thresholds for cell count (≥25), gene count (≥1), expression fraction (≥0.05), and log2-normalized differential expression (≥0.25). In the final complex contrast, we applied a more lenient log2-normalized fold change threshold of 0.125 and set p-values to 1 as they were not meaningful in this context.

## References

1. National Health Commission of People’s Republic of China & National Administration of Traditional Chinese Medicine. Diagnosis and treatment protocol for COVID‐19 patients (Tentative 10th Version). *Health Care Science*. Published online 2023. doi:10.1002/hcs2.36

2. Hao Y, Hao S, Andersen-Nissen E, et al. Integrated analysis of multimodal single-cell data. *Cell*. 2021;184(13):3573-3587.e29. doi:10.1016/j.cell.2021.04.048

3. Ahern DJ, Ai Z, Ainsworth M, et al. A blood atlas of COVID-19 defines hallmarks of disease severity and specificity. *Cell*. 2022;185(5):916-938.e58. doi:10.1016/j.cell.2022.01.012

4. Borcherding N, Bormann NL. scRepertoire: An R-based toolkit for single-cell immune receptor analysis. Published online January 27, 2020. doi:10.12688/f1000research.22139.1

5. Gupta NT, Vander Heiden JA, Uduman M, Gadala-Maria D, Yaari G, Kleinstein SH. Change-O: a toolkit for analyzing large-scale B cell immunoglobulin repertoire sequencing data. *Bioinformatics*. 2015;31(20):3356-3358. doi:10.1093/bioinformatics/btv359

6. Madeira F, Pearce M, Tivey ARN, et al. Search and sequence analysis tools services from EMBL-EBI in 2022. *Nucleic Acids Res*. 2022;50(W1):W276-W279. doi:10.1093/nar/gkac240

7. Wu T, Hu E, Xu S, et al. clusterProfiler 4.0: A universal enrichment tool for interpreting omics data. *Innovation*. 2021;2(3). doi:10.1016/j.xinn.2021.100101

8. Liu L, Wang P, Nair MS, et al. Potent neutralizing antibodies against multiple epitopes on SARS-CoV-2 spike. *Nature*. 2020;584(7821):450-456. doi:10.1038/s41586-020-2571-7

9. Protein complex prediction with AlphaFold-Multimer | bioRxiv. Accessed March 4, 2024. https://www.biorxiv.org/content/10.1101/2021.10.04.463034v1

10. Browaeys R, Gilis J, Sang-Aram C, et al. MultiNicheNet: a flexible framework for differential cell-cell communication analysis from multi-sample multi-condition single-cell transcriptomics data. Published online June 10, 2023:2023.06.13.544751. doi:10.1101/2023.06.13.544751
