# Supplementary material for: Myeloid‐Driven Immune Suppression Subverts Neutralizing Antibodies and T Cell Immunity in Severe COVID‐19
Source: J Med Virol. 2025 Apr 4;97(4):e70335. doi: 10.1002/jmv.70335 (PMC11969634; doi:10.1002/jmv.70335)
Supplement: Supplementary file 29 — Supporting Table Captions 241125. [file JMV-97-e70335-s011.docx]

**Supplementary Table Captions**

**Supplementary Table 1.** Overview of Basic Information on Clinical Samples. Sheet named “Supplementary_Table_1A” provides details on clinical sample information, sheet named “Supplementary_Table_1B” and “Supplementary_Table_1C” presents statistics on sample quality, and sheet named “Supplementary_Table_1D” includes cell count statistics.

**Supplementary Table 2.** Top 5 most abundant clonotypes information on each group.

**Supplementary Table 3.** Sequence information of expressed monoclonal antibodies in vitro.
