# Supplementary material for: Myeloid‐Driven Immune Suppression Subverts Neutralizing Antibodies and T Cell Immunity in Severe COVID‐19
Source: J Med Virol. 2025 Apr 4;97(4):e70335. doi: 10.1002/jmv.70335 (PMC11969634; doi:10.1002/jmv.70335)
Supplement: Supplementary file 4 — Supporting Figure 4. Visualization of cell annotation marker genes. [file JMV-97-e70335-s022.pdf]

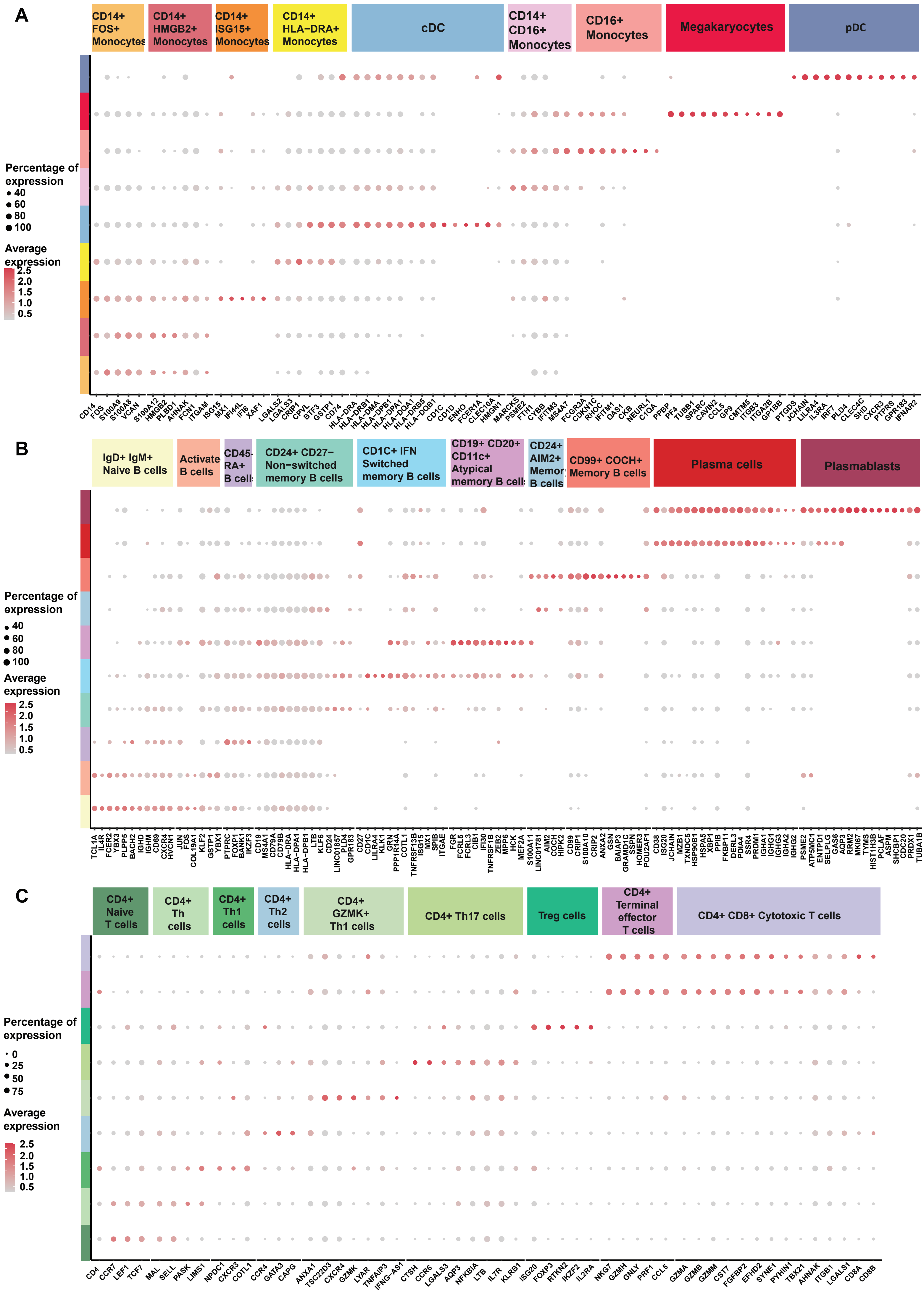
A
CD14+ FOS+ Monocytes
CD14+ HMGB2+ Monocytes
CD14+ ISG15+ Monocytes
CD14+ HLA−DRA+ Monocytes
cDC
CD14+ CD16+ Monocytes
CD16+ Monocytes
Megakaryocytes
pDC
Percentage of expression
40
60
80
100
Average expression
2.5
2.0
1.5
1.0
0.5
CD14 FOS S100A9 S100A8 VCAN S100A12 HMGB2 PLBD1 AHNAK FCN1 ITGAM ISG15 MX1 IFI44L IFI6 XAF1 LGALS2 LGALS3 CRIP1 CPVL BTF3 GSTP1 CD74 HLA−DRA HLA−DRB1 HLA−DMA HLA−DPB1 HLA−DPA1 HLA−DQA1 HLA−DRB5 HLA−DQB1 CD1C CD1D ENHO FCER1A CLEC10A HMGN1 MARCKS PSME2 FTH1 CYBB IFITM3 MS4A7 FCGR3A CDKN1C RHOC IFITM1 OAS1 CKB NEURL1 C1QA PPBP PF4 TUBB1 SPARC CAVIN2 CCL5 GP9 CMTM5 ITGB3 ITGA2B GP1BB PTGDS JCHAIN LILRA4 IL3RA IRF7 PLD4 CLEC4C SHD CXCR3 PTPRS GPR183 IFNAR2
B
IgD+ IgM+ Naive B cells
Activated B cells
CD45RA+ B cells
CD24+ CD27− Non−switched memory B cells
CD1C+ IFN Switched memory B cells
CD19+ CD20+ CD11c+ Atypical memory B cells
CD24+ AIM2+ Memory B cells
CD99+ COCH+ Memory B cells
Plasma cells
Plasmablasts
Percentage of expression
40
60
80
100
Average expression
2.5
2.0
1.5
1.0
0.5
C
CD4+ Naive T cells
CD4+ Th cells
CD4+ Th1 cells
CD4+ Th2 cells
CD4+ GZMK+ Th1 cells
CD4+ Th17 cells
Treg cells
CD4+ Terminal effector T cells
CD4+ CD8+ Cytotoxic T cells
Percentage of expression
0
25
50
75
Average expression
2.5
2.0
1.5
1.0
0.5
CD4 CCR7 LEF1 TCF7 MAL SELL PASK LIMS1 NPDC1 CXCR3 COTL1 CCR4 GATA3 CAPG ANXA1 TSC22D3 CXCR4 GZMK LYAR TNFAIP3 IFNG−AS1 CTSH CCR6 LGALS3 AQP3 NFKBIA LTB IL7R KLRB1 ISG20 FOXP3 RTKN2 IKZF2 IL2RA NKG7 GZMH GNLY PRF1 CCL5 GZMA GZMB GZMM CST7 FGFBP2 EFHD2 SYNE1 PYHIN1 TBX21 AHNAK ITGB1 LGALS1 CD8A CD8B
