# Supplementary material for: Myeloid‐Driven Immune Suppression Subverts Neutralizing Antibodies and T Cell Immunity in Severe COVID‐19
Source: J Med Virol. 2025 Apr 4;97(4):e70335. doi: 10.1002/jmv.70335 (PMC11969634; doi:10.1002/jmv.70335)
Supplement: Supplementary file 10 — Supporting Figure 10. Differential ligand‐receptor interactions across various clinical conditions. [file JMV-97-e70335-s005.pdf]

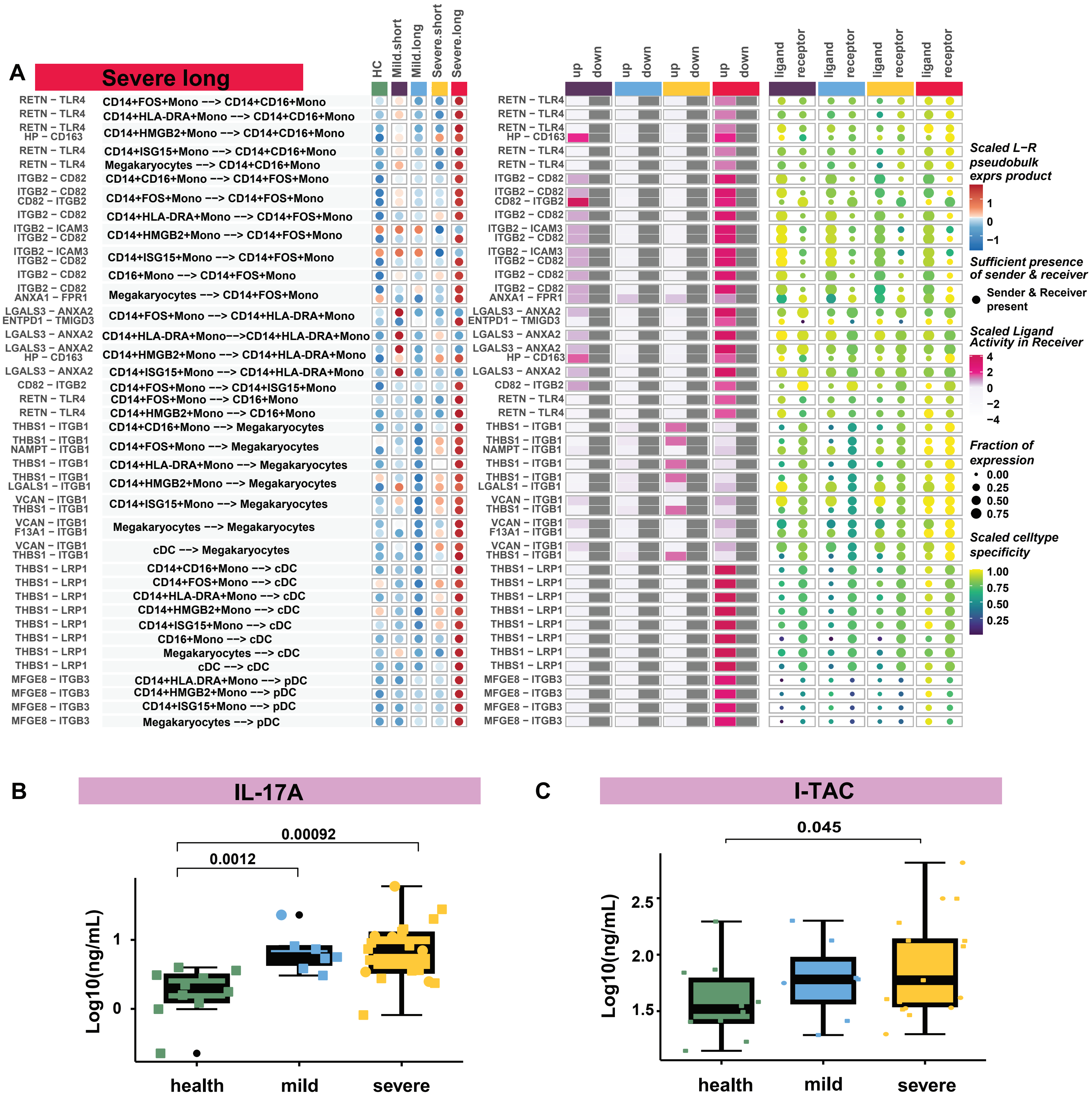
A
Severe long
HC
Mild.short
Mild.long
Severe.short
Severe.long
up
down
ligand
receptor
RETN − TLR4 CD14+FOS+Mono --> CD14+CD16+Mono
RETN − TLR4 CD14+HLA-DRA+Mono --> CD14+CD16+Mono
RETN − TLR4 HP − CD163 CD14+HMGB2+Mono --> CD14+CD16+Mono
RETN − TLR4 CD14+ISG15+Mono --> CD14+CD16+Mono
RETN − TLR4 Megakaryocytes --> CD14+CD16+Mono
ITGB2 − CD82 CD14+CD16+Mono --> CD14+FOS+Mono
ITGB2 − CD82 CD82 − ITGB2 CD14+FOS+Mono --> CD14+FOS+Mono
ITGB2 − CD82 CD14+HLA-DRA+Mono --> CD14+FOS+Mono
ITGB2 − ICAM3 ITGB2 − CD82 CD14+HMGB2+Mono --> CD14+FOS+Mono
ITGB2 − ICAM3 ITGB2 − CD82 CD14+ISG15+Mono --> CD14+FOS+Mono
ITGB2 − CD82 CD16+Mono --> CD14+FOS+Mono
ITGB2 − CD82 ANXA1 − FPR1 Megakaryocytes --> CD14+FOS+Mono
LGALS3 − ANXA2 ENTPD1 − TMIGD3 CD14+FOS+Mono --> CD14+HLA-DRA+Mono
LGALS3 − ANXA2 CD14+HLA-DRA+Mono-->CD14+HLA-DRA+Mono
LGALS3 − ANXA2 HP − CD163 CD14+HMGB2+Mono --> CD14+HLA-DRA+Mono
LGALS3 − ANXA2 CD14+ISG15+Mono --> CD14+HLA-DRA+Mono
CD82 − ITGB2 CD14+FOS+Mono --> CD14+ISG15+Mono
RETN − TLR4 CD14+FOS+Mono --> CD16+Mono
RETN − TLR4 CD14+HMGB2+Mono --> CD16+Mono
THBS1 − ITGB1 CD14+CD16+Mono --> Megakaryocytes
THBS1 − ITGB1 NAMPT − ITGB1 CD14+FOS+Mono --> Megakaryocytes
THBS1 − ITGB1 CD14+HLA-DRA+Mono --> Megakaryocytes
THBS1 − ITGB1 LGALS1 − ITGB1 CD14+HMGB2+Mono --> Megakaryocytes
VCAN − ITGB1 THBS1 − ITGB1 CD14+ISG15+Mono --> Megakaryocytes
VCAN − ITGB1 F13A1 − ITGB1 Megakaryocytes --> Megakaryocytes
VCAN − ITGB1 THBS1 − ITGB1 cDC --> Megakaryocytes
THBS1 − LRP1 CD14+CD16+Mono --> cDC
THBS1 − LRP1 CD14+FOS+Mono --> cDC
THBS1 − LRP1 CD14+HLA-DRA+Mono --> cDC
THBS1 − LRP1 CD14+HMGB2+Mono --> cDC
THBS1 − LRP1 CD14+ISG15+Mono --> cDC
THBS1 − LRP1 CD16+Mono --> cDC
THBS1 − LRP1 Megakaryocytes --> cDC
THBS1 − LRP1 cDC --> cDC
MFGE8 − ITGB3 CD14+HLA.DRA+Mono --> pDC
MFGE8 − ITGB3 CD14+HMGB2+Mono --> pDC
MFGE8 − ITGB3 CD14+ISG15+Mono --> pDC
MFGE8 − ITGB3 Megakaryocytes --> pDC
Scaled L−R pseudobulk exprs product
1
0
−1
Sufficient presence of sender & receiver
Sender & Receiver present
Scaled Ligand Activity in Receiver
4
2
0
−2
−4
Fraction of expression
0.00
0.25
0.50
0.75
Scaled celltype specificity
1.00
0.75
0.50
0.25
B
IL-17A
0.00092
0.0012
Log10(ng/mL)
1
0
health
mild
severe
C
I-TAC
0.045
Log10(ng/mL)
2.5
2.0
1.5
health
mild
severe
